# QSAR analysis on a large and diverse set of potent phosphoinositide 3-kinase gamma (PI3Kγ) inhibitors using MLR and ANN methods

**DOI:** 10.1038/s41598-022-09843-0

**Published:** 2022-04-12

**Authors:** Fereydoun Sadeghi, Abbas Afkhami, Tayyebeh Madrakian, Raouf Ghavami

**Affiliations:** 1 Faculty of Chemistry, Bu-Ali Sina University, Hamedan, Iran; 2 D-8 International University, Hamedan, Iran; 3 Autophagy Research Center, Shiraz University of Medical Sciences, Shiraz, Iran; 4 Chemometrics Laboratory, Chemistry Department, Faculty of Science, University of Kurdistan, Sanandaj, Iran

**Keywords:** Computational models, Computational platforms and environments, Computational biology and bioinformatics

## Abstract

Phosphorylation of PI3Kγ as a member of lipid kinases-enzymes, plays a crucial role in regulating immune cells through the generation of intracellular signals. Deregulation of this pathway is involved in several tumors. In this research, diverse sets of potent and selective isoform-specific PI3Kγ inhibitors whose drug-likeness was confirmed based on Lipinski’s rule of five were used in the modeling process. Genetic algorithm (GA)-based multivariate analysis was employed on the half-maximal inhibitory concentration (IC_50_) of them. In this way, multiple linear regression (MLR) and artificial neural network (ANN) algorithm, were used to QSAR models construction on 245 compounds with a wide range of pIC_50_ (5.23–9.32). The stability and robustness of the models have been evaluated by external and internal validation methods (R^2^ 0.623–0.642, RMSE 0.464–0.473, F 40.114, Q^2^_LOO_ 0.600, and R^2^_y-random_ 0.011). External verification using a wide variety of structures out of the training and test sets show that ANN is superior to MLR. The descriptors entered into the model are in good agreement with the X-ray structures of target-ligand complexes; so the model is interpretable. Finally, Williams plot-based analysis was applied to simultaneously compare the inhibitory activity and structural similarity of training, test and validation sets.

## Introduction

Phosphatidylinositol 3-kinases (PI3Ks) are a group of plasma membrane-associated lipid kinases-enzymes that their phosphorylation plays a critical regulatory role in the cellular processes^1,2^. In The cellular regulatory mechanism, kinases and phosphatases catalyze activation and deactivation processes via phosphorylation and dephosphorylation of PI3Ks, respectively^3^. In response to various external stimuli such as oncogenes, growth factors, hormones, and environmental variations, PI3Ks are phosphorylated through conversions of phosphatidylinositol (4,5)-bisphosphate (PIP2) to phosphatidylinositol (3,4,5)-trisphosphate (PIP3)^4,5^. PIP3 serves as a docking site of effector proteins such as protein kinase B (PKB/Akt) that act as the second messenger molecule in the cellular membranes^1^. This intracellular signaling pathway has an important role in regulating diverse cellular processes such as cell growth, differentiation, proliferation, survival, and migration^6–8^. Reversible phosphorylation of inositol lipids controls diverse functions in cells. Deregulation of this pathway occurs by various genetic and epigenetic mechanisms in a wide range of tumors^9–11^. PI3Ks are divided into classes I, II, and III based on the differences in their structures and specific substrates^12,13^. According to the regulator proteins and signaling pathways, class I PI3Ks are further subdivided into class IA and class IB. Class IA PI3Ks contains three enzyme isoforms, PI3Kα, PI3Kβ, and PI3Kδ; while PI3Kγ is the only member of class IB and its corresponding signal primarily is generated by G-protein coupled receptors (GPCRs). PI3Kδ and PI3Kγ can generate intracellular signals to regulate immune cells. These two enzyme isoforms are being investigated for cancer treatment in the clinic^14–16^. PI3Kα and PI3Kβ are involved in the regulation of cell survival and metabolism^17–20^. Overall, PI3Kγ controls a critical switch between immune stimulation and suppression during inflammation and cancer^21^. The abnormal expression of PI3Kγ is the result of the mutation and deficiency of phosphatase and tensin homolog on chromosome ten (PTEN)^1,22^.

In competition with ATP, PI3Kγ inhibitors bind to the ATP cofactor binding site in the active form of kinases to block PI3Kγ activity through stabilizing inactive kinase conformations^23^. PI3Kγ has attracted attention as a potential drug target in treating advanced solid tumors, leukemia, inflammatory, and various autoimmune diseases. Over the past two decades, PI3Ks (especially PI3Kγ) inhibitors have been attracting extensive interest and more than 600 medicinal chemistry-based publications and patents to date show the importance of these compounds^24^. Enormous efforts have been dedicated to the development of highly efficient, safer, potent, and selective isoform PI3Kγ inhibitors. Gangadhara et al. discovered a class of PI3Kγ inhibitors. They proposed that the cyclopropyl ethyl moiety of these inhibitors induces a significant conformational change in both the kinase and helical domains of PI3Kγ which results in blocking the ATP–binding site^25^. Other research groups also discovered a series of potent and selective PI3Kγ inhibitors, some of which are: azaisoindolinones^26^, benzothiazoles^27^, 7-substituted triazolopyridine (CZC24758)^28^, IPI-549 (through optimization of isoquinolinone)^29^, and 7-azaindole isoindolinone^30^. Drew et al. designed potent PI3Kγ inhibitors based on the differences of IPI-549 and AZ2 in the binding modes interaction with ATP binding site of PI3Kγ^31^. SAR study on 6-aryl-2-amino-triazolopyridines was performed by Bell et al.^32^ Zhu et al. provide an overview to discuss the structure‐selectivity‐activity relationship of existing clinical PI3Kγ inhibitors^33^ and, ultimately Taha et al. used ligand‐based modeling and virtual screening followed by in vitro analysis to discover nanomolar PI3Kγ inhibitors^34^.

Despite all these efforts, indeed selecting isoenzyme compounds is difficult; due to the high sequence homology among the PI3K isoforms. Therefore, the discovery and development of PI3Kγ-selective inhibitors are still quite challenging.

Cost and time consumption are disadvantages of the in vivo-in vitro assays during drug development. QSAR analysis in a primary screening through selecting and proposing the most potent drug candidates causes to prioritize the synthesis of effective drugs; subsequently, pharmaceutical research can be more efficient. Halder and Cordeiro reported the QSAR-Co tool for predicting the activity of inhibitor compounds against different isoforms of PI3Ks, under various experimental conditions^35^.

In this research, QSAR analysis was carried out on a large and diverse set of potent and selective isoform-specific PI3Kγ inhibitors using an artificial neural network and multivariate linear regression. The interpretability, clarity, and understandability of the models presented by MLR make it a good choice for modeling. At the same time, the complex relationship between the chemical structures of PI3Kγ inhibitors and their biological response is the best justification for using the ANN-based nonlinear method.

Classification as another aspect of QSAR modeling can also be mentioned that was developed on qualitative categorical responses^36^. In the simplest case, chemical compounds are classified into two categories active/inactive based on their biological activity. The mapping function based on the output variables is employed to predict the class or category for a given observation. In the case of two classes, binary classification is applied. Due to the high sequence homology among the PI3K isoforms, here we used regression-based QSAR models for a quantitative study.

Most of the compounds used in this research have been recently synthesized or evaluated experimentally. Also, to increase the application domain of the models, these compounds were investigated in a wide range of pIC_50_ (− log IC_50_). The selectivity of these compounds for PI3Kγ over the other PI3K isoforms is confirmed by X-ray crystallography. To take into account safety profiles related to absorption, distribution, metabolism, elimination, and toxicity (ADMET) during the prediction of activity, Speck-Planche and Cordeiro introduced the multitasking model for quantitative structure biological effect relationships (mtk-QSBER)^37–39^. In the present work, Lipinski’s rule of five was used to check the drug-likeness of compounds^40^. Moreover, to further assess the models, external verification was performed using another group of PI3Kγ inhibitors with high structural diversity and a wide range of activity.

## Materials and methods

### Data sets

In this study, 245 compounds of PI3Kγ inhibitors collected from published literature^18,24,28–32,41^ were used for QSAR modeling. It is worth mentioning that, after removing duplicate molecules from the above references, a data set consisting of 256 molecules was collected. Then 11 compounds were removed from the data set, including seven molecules that were too different structurally for investigation in the application domain of the models and four molecules whose pIC_50_ values were out of the considered range significantly. Thus, the final data set was reduced to 245 molecules. All minimum inhibitory concentration (IC_50_) values of molecules were converted into the corresponding pIC_50_. The structure of these molecules and their corresponding values of PI3Kγ inhibitory activity (pIC_50_) are presented in Supplementary Table S1. Also, simplified molecular input line entry specification (SMILES) strings of molecules are provided in Supplementary Table S2.

### Drug-likeness assessment

For assessment of drug-likeness of a molecule, Lipinski’s rule of five was employed^40^. Based on the distribution of molecular properties (molecular weight, H-bond donors, H-bond acceptors, and logP) among several thousand drugs of USAN (United States Adopted Name) data set, the percents of drugs that are predicted to have poor absorption or permeation are specified in Table 1. In this Table, the ClogP parameter is calculated based on the substructure (atomic group) contribution. In comparison with other estimation methods, ClogP has a better agreement with experimental results. This parameter as a criterion of lipophilicity affects the permeability, accumulation, absorption, bioavailability, and drug cytotoxicity.

**Table 1:** Assessment of the drug-likeness (solubility and permeability of a molecule) based on Lipinski’s rule of five.

properties	Percent of USAN^a^ data set out of (cutoff) Lipinski’s rule of five	Percent cutoff compounds in the present study (%)
MW^b^	More than 500 daltons (11%) to (22%) in the entire data set	14.3
	More than 600 daltons (8%)	1.63
nHDon^c^	More than 5 (8%)	0.82
nHAcc^d^	More than 10 (12%)	10.61
CLogP	Greater than 5 (10%)	3.67
TPSA(NO)^e^	Greater than 140 Ǻ^2^	9.32
RBN^f^	More than 10	0.82
nAT^g^	More than 70	2.04

^a^United States Adopted Name.

^b^Molecular weight.

^c^H-bond donors (Total NH and OH).

^d^H-bond acceptors (The sum of nitrogen and oxygen atoms).

^e^Polar surface area (only nitrogen and oxygen atoms considered).

^f^Rotatable bonds number.

^g^Number of atoms.

For 245 compounds involved in the modeling process, the aforementioned properties were calculated using Dragon 5.5 software package^42^, except the ClogP that the Data warrior software^43^ was used to calculate it. Calculated parameters for these 245 compounds are presented in Supplementary Table S3. The result of checking them by Lipinski’s rule of five confirmed their favorable drug-likeness as shown in Table 1.

### Descriptors calculation and feature selection

In the first stage of the molecular modeling, SMILES strings of the structures were saved in SDF (Structure Data File) format; then, Open Babel software^44^ was applied to convert them into the HyperChem HIN format. Following the modeling process in the HyperChem 8 software package^45^, the molecular mechanics force field (MM^+^) procedure was used to pre-optimization of 3D structures to lower energy levels. Then, the Semi-empirical methods including PM3 and AM1, which belong to quantum chemistry methods, were used to optimize the structures geometrically and electronically, respectively. Root mean square gradient equal to 0.001 kcal Å^−1^ mol^−1^ was determined as the critical value of optimization. The most stable optimized conformer of each structure was selected and saved. Subsequently, Dragon 5.5 software package^42^ was used to compute 2D autocorrelation descriptors (a total of 96 of such descriptors) and, using the former optimized geometrics, three-dimensional (3D) descriptors including Randic molecular profiles, geometrical, RDF, 3D-MoRSE, WHIM, and GETAWAY categories (a total of 41, 74, 150, 160, 99 and 197 of such descriptors, respectively).

Among 22 different classes of descriptors computable by the Dragon software, 3D and 2D autocorrelation descriptors most probably have a successful performance in 2D-QSAR modeling based on the results of our previous studies on the inhibitory activity of anti-cancer drug candidates^46,47^. More details about these descriptors and the superior features that make them most appropriate for modeling are provided at the end of the manuscript. To avoid overfitting, during the QSAR model development, objective feature selection was used to reduce the redundant and unnecessary information. In this way, descriptors that are zero and constant for all molecules were discarded from the descriptors pool. Additionally, as a rule highly correlated (R > 0.90), from each pair of descriptors that have a correlation coefficient greater than 0.9, only one remains in the descriptors pool and the other one is eliminated. Following the feature selection, based on the above described, the number of descriptors is reduced from 817 to 290 numbers, up to this stage. Subsequently, subjective feature selection involves the genetic algorithm tool in selecting the most relevant set of descriptors that were not collinear^48^.

This algorithm is based on the theoretical principles of Darwin's theory of evolution and is highly welcomed to multivariate analysis. GA runs based on the following steps:

Initially, many subsets of descriptors are randomly generated that serve as chromosomes, where the descriptors included in each subset play the role of the gene. Then, for each subset of descriptors (each chromosome), the MLR model was developed separately. Based on the goodness prediction of inhibitory activity, the chromosomes are evaluated. The correlation coefficient (Q^2^) value plays the role of the fitness function which is calculated based on employing the leave-one-out cross-validation (LOO-CV) method on each chromosome separately **(**LOO-CV is described in the next part of this research). Each subset of descriptors located on a chromosome is encoded with a string of binary 1 and 0 values. Based on the modeling results, if the descriptor corresponding to each gene is effective in predicting the inhibitory activity, its value is equal to 1, otherwise, it is taken to be 0. This function leads to the expulsion of the worst subsets. Then two types of modification are operated randomly including crossover through the replacement of the corresponding sections of the two parent chromosomes from two points (Duble) and mutation that is operated through randomly changing a position of a parent chromosome to change its value. In this way, the child chromosomes are extracted, and according to what was previously described, their fitness is computed. The best children replace the worst parent to improve the primary population. This process is subsequently repeated until the most relevant set of descriptors with the highest convergence are selected or criteria defined to stop the algorithm are achieved. In this work using MATLAB software^49^, GA was run based on the optimal parameters presented in Table 2. By selecting the most suitable descriptors, the number of them reduced from 290 to 56 cases.

**Table 2:** Parameters of the genetic algorithm.

Cross validation	Random
Number of subsets	4
Window width	2
% Initial terms	20
Max generation	100
% at Convergence	70
Mutation rate	0.003
Cross-over	Double

### Dataset splitting

One of the most common methods in QSAR model evaluation is external validation that is performed through dividing the whole data set into the training and test sets by a ratio of 4:1. It is highly critical that both groups must be a reliable representatives of the entire dataset in terms of molecular structure, biological activity, and physicochemical property.

Among the various methods of data splitting, DUPLEX^50^ and Kennard–Stone^51^ algorithms are more welcomed; because, they perform data splitting according to the aforementioned conditions, which are introduced below.

### The DUPLEX algorithm based on the PCA

Considering the large number of structural descriptors, this approach causes the total space of structures to be counted for data splitting and helps to uniform distribution (homogenity) of data set into the training and test. Based on this algorithm, first, principal component analysis (PCA) was performed on the entire data set including 290 relevant descriptors. Then a new activity was calculated through establishing a principal component regression (PCR) between original experimental inhibitory activity and PCs. Subsequently, the results were provided to the DUPLEX algorithm to splitting the data set based on the following process:

In the beginning, the two most distant (i.e. most dissimilar) objects are removed from the dataset and placed into the training set. From the remaining points, the next pair which are farthest apart are picked up and placed into the test set. Among the remaining points, two points are moved to the training set with the greatest distance from each other, again. Then, from the remaining objects, the one which is furthest away from those previously selected as the training set, is moved to the test set. The process is repeated until each set contains a certain number of molecules. By employing this method, the uniform distribution of data between the training and test sets was guaranteed not only in the properties but also in the structures.

### Kennard–Stone algorithm

In a similar approach to DUPLEX**,** Kennard–Stone algorithm ensures that each point of the test set is close to at least one point of the training set. This algorithm uses the following equation to split a dataset into training and test set:

1 $$\mathrm{Objective \; function }= \sum_{\mathrm{i}=1}^{\mathrm{k}+1}\left\{{[\upmu (\mathrm{i})}_{\mathrm{train}}-{\upmu \left(\mathrm{i}\right)}_{\mathrm{test}}]+{[\upsigma (\mathrm{i})}_{\mathrm{train}}-{\upsigma \left(\mathrm{i}\right)}_{\mathrm{test}}]\right\},$$

k represents the number of inputs, while μ and σ are labels for mean and standard deviation of the input or output variable, respectively.

Euclidean distance *ED*_*x*_ (*p,q*) is employed by this algorithm to ensure the uniform distribution of the selected subset in the data space as below:

2 $${ED}_{x}\left(p, q\right)=\sqrt{\sum_{j=1}^{n}{[{x}_{p}\left(j\right)-{x}_{q}(j)]}^{2}} \mathrm{p},\mathrm{ q}\in \left[1, M\right].$$

Based on the several reports, in the data splitting process, the superiority and high quality of the DUPLEX algorithm over other methods have been confirmed^52–54^ so in this research, the modeling process was performed using the training set obtained from DUPLEX.

In our research following data splitting by DUPLEX algorithm, Kennard-stone algorithm, and Random data splitting by Minitab software^55^, GA-MLR models were established on the training set and then generalized to the test and validation sets. More details of data splitting and model validation using these methods are presented in the section “QSAR modeling results”.

### Statistical factors and methods used in the model evaluation and validation

Since the external and internal validation of the model is an essential step in QSAR analysis, several statistical parameters were employed to assess the performance of the models, which are briefly described in Table 3, and equations used in calculation them have been presented. Williams plot-based analysis is explained later (Determination of the application domain of the model).

**Table 3:** Model performance parameters and their related equations.

Statistical parameters	Brief definition	Equations^a^
Correlation coefficient	R was used to investigate the correlation between the descriptors entered in the models	$\mathrm{R}=\frac{\sum \left({x}_{\mathrm{i}}-\overline{x }\right)\left({\mathrm{y}}_{\mathrm{i}}-\overline{\mathrm{y} }\right)}{\sqrt{\sum {\left({x}_{\mathrm{i}}-\overline{x }\right)}^{2}\sum {\left({\mathrm{y}}_{\mathrm{i}}-\overline{\mathrm{y} }\right)}^{2}}}$ − 1 ≤ R ≤ 1
The square correlation coefficient of multiple linearities (R^2^)	R^2^ is used to indicate the goodness of fit	${R}^{2}=1-\frac{\sum {\left({y}_{i}-{\widehat{y}}_{i}\right)}^{2}}{\sum {\left({y}_{i}-{\overline{y} }_{i}\right)}^{2}}$
Adjusted R squared (R^2^_adj_)	R^2^_adj_ is measured based on descriptors that really help in explaining the dependent variable	${R}_{adj}^{2}=1-\left(1-{R}^{2}\right)\left[\left(\frac{n-1}{n-p-1}\right)\right]$
Fisher's test (F)	F used to calculate the variance established between groups to the variance within groups. The larger value for F ratio indicates that the model ability is better to predict pIC_50_ in the training set	$F=\frac{\frac{\sum_{i}{\left({\widehat{y}}_{i}-{\overline{y} }_{i}\right)}^{2}}{p}}{\frac{{\sum }_{i}{\left({y}_{i}-{\widehat{y}}_{i}\right)}^{2}}{n-p-1}}$
Root mean square error of prediction (RMSEP)	RMSEP based on the difference between predicted and observed values of pIC_50_ for the test set represents the model's prediction ability	$RMSEP=\sqrt{\frac{{\sum }_{i=1}^{n}{\left({\widehat{y}}_{i}-{y}_{i}\right)}^{2}}{n}}$
The square correlation coefficient for leave-one-out cross-validation (Q^2^_LOO_)	Q^2^_LOO_ is calculated based on the predicted values of pIC_50_, during perform LOO-CV	Predicted values of pIC_50_ calculated from this method, are placed in the R squared equation
Prediction residual error sum of squares (PRESS)	PRESS is determined the difference between experimental and predicted values of pIC_50_ for the total data set during the LOO-CV processing	$\mathrm{PRESS}={\sum }_{i=1}^{n}{\left({y}_{i obs}-{y}_{i pred}\right)}^{2}$

^a^y_i_, ŷ_i_, and _i_ are experimental, predicted, and average values of pIC_50_ respectively; p: the number of descriptors in the model; n: the number of samples.

### Model development

The SPSS software^56^ establishes multivariate linear regression by receiving the data matrix consist of the most suitable 3D and 2D autocorrelations descriptors selected by GA and the corresponding inhibitory activity of each *x*-vectors. According to the stepwise procedure, the entry of the descriptors into the model continues until the R^2^ value is strengthened significantly and the root mean square error (RMSE) value is weakened by entering the new descriptor. Of course, simultaneously the value of the Fisher's test (F) parameter is controlled so that it accepts its optimum value. Very high and very low values of F lead to overfitting and underfitting errors, respectively. One of the valid criteria in monitoring the optimum value of F is the variance inflation factor (VIF) which shows the correlation between the descriptors (described in continue further); during the modeling process, its value must be kept less than 5. In addition, the coefficients of the descriptors should be acceptable values based on their standard deviation. The criterion for stopping the entry of descriptors into the model is that by entering the new descriptor, the statistical performance factors do not improve significantly.

The above approach prevents overfitting. The variance inflation factor (VIF) test, ensures that the modeling process is not accompanied with multicollinearity and is calculated as below:

3 $$\mathrm{VIF}=\frac{1}{1-{\mathrm{R}}_{\mathrm{j}}^{2}},$$

where R^2^_j_ is the square of the correlation coefficient between descriptors during the model development. VIF equal to 1 indicates that the j-th descriptor is not correlated to the remaining ones. To accept the model, the VIF value should be between 1 and 5, but in the case of VIF values higher than 10, there is significant multicollinearity; so the model must be corrected.

### Leave-one-out cross-validation (LOO-CV)

During the LOO-CV as one of the internal validation methods, each molecule is removed from the data matrix and the remaining molecules are employed to model development. Using the extracted model, the molecule that was kept out is predicted; this process is repeated for all molecules.

### Y-randomization test

To ensure that the developed model does not arise from chance, y-randomization is performed through the scrambled biological activity. This procedure is repeated twenty times randomly; then a new regression is established using the same parameters of the original model. Low values of R^2^_y-random_ and Q^2^_y-random_ in the new models with shuffled pIC_50_, confirm the efficiency and robustness of the main developed model.

### Description of the artificial neural network theory, briefly

Artificial neural networks algorithms inspired by biological neural networks in the human brain^57^. The application of ANN is based on this hypothesis that a given training data to construct a model, can learn and generalize from previously seen examples. During the learning, the algorithm extracts the rules and relationships governing the experimental data through their processing. The extracted information is inducted into the network. ANN, as a nonlinear modeling technique, has been used extensively in QSAR analysis and constructed from an input layer, hidden layer(s), and an output layer. The number of input neurons to the network is equal to descriptors used in the linear model development. The weight parameter determines the effect of the input layer on the output layer, which is adjusted during training with the feed-forward back-propagation approach. The trial and error procedure on the training set is employed to optimize the size of the hidden layers. The criterion for this assessment is the average square error (MSE) which acts as a performance function. In the present study, the Bayesian regularization algorithm was used to train with a feed-forward approach and sigmoid as a hidden layer transfer function was employed. Experimental pIC_50_ acts as one output layer. A large number of hidden layers causes the developed model to have an overfitting problem; however, a too-small number of hidden layers leads to fault tolerance and weakens the generalization capability of the net. In the current study, the implementation of the above approach resulted in the 10-3-1 network architecture. Separate validation of the model was performed by one-tenth of the training set selected randomly. In this way, the performance of the ANN was monitored; through evaluation predicted values of the validation set during the training of the network. The training is stopped when the results for the validation set are not significantly improved.

## Results

### QSAR modeling results

The DUPLEX algorithm was employed to dividing total 245 PI3Kγ inhibitors into the training (196 molecules) and test sets (49 molecules). The MLR model was developed using a relevant set of descriptors selected by GA and was evaluated by the test set

4 $$\begin{aligned} {\text{pIC}}_{{{5}0}} & = { 9}.{67}0 \, \left( { \pm 0.{935}} \right) \, - \, 0.{891 }\left( { \pm 0.{158}} \right){\text{Mor12p}} + \, 0.{246 }\left( { \pm 0.0{46}} \right){\text{RDF}}0{1}0{\text{e}} - \, 0.{6}0{4 }\left( { \pm 0.{115}} \right){\text{Mor14u}} \\ & \quad - \, 0.{54}0 \, \left( { \pm 0.{11}0} \right){\text{Mor15m}} - { 1}.{727 }\left( { \pm 0.{3}0{2}} \right){\text{GATS6p}} - \, 0.{732 }\left( { \pm 0.{183}} \right){\text{Mor19m}} + \, 0.0{38 }\left( { \pm 0.00{8}} \right){\text{Te}} \\ & \quad - { 12}.{244 }\left( { \pm {4}.{271}} \right){\text{G2v}} - 0.0{39 }\left( { \pm 0.0{14}} \right){\text{Mor}}0{\text{2v}} + \, 0.{718 }\left( { \pm 0.{293}} \right){\text{GATS4p,}} \\ \end{aligned}$$

$${\text{n}}_{{{\text{training}}}} = { 196},{\text{ R}}^{{2}} = \, 0.{623},{\text{ R}}^{{2}}_{{{\text{adj}}}} = \, 0.{6}0{2},{\text{ RMSE }} = \, 0.{473},{\text{ F }} = { 3}0.{546,}$$

$${\text{n}}_{{{\text{test}}}} = { 49},{\text{ R}}^{{2}} = \, 0.{662},{\text{ RMSEP }} = \, 0.{451}{\text{.}}$$

R^2^ and RMSE values calculated for training and test sets using the DUPLEX algorithm, Kennard–Stone algorithm, and random data splitting are provided in Table 4.

**Table 4:** Calculated R^2^ and RMSE parameters for training and test sets separately, following the data splitting process by three methods.

Method	DUPLEX algorithm		Kennard–Stone algorithm		Random data splitting by Minitab software	
	Training set	Test set	Training set	Test set	Training set	Test set
R^2^	0.623	0.662	0.610	0.635	0.631	0.634
RMSE	0.473	0.451	0.476	0.492	0.489	0.476

**Figure 1:**
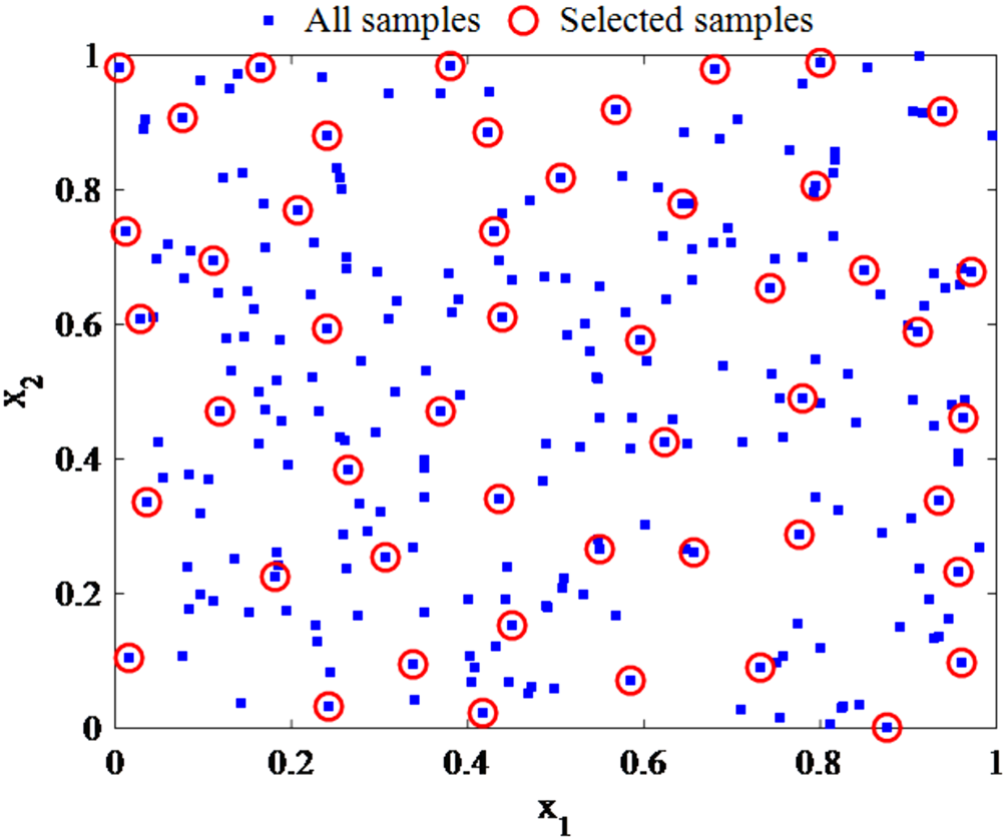
Plot of the data splitting pattern using Kennard–Stone algorithm on 245 compounds.

Figure 1 is plotted based on the data splitting pattern by Kennard–Stone algorithm on 245 compounds used in this study.

As a general rule, a QSAR model is considered to be predictive if calculated values of R^2^, Q^2^, and R^2^_pred_ are higher than 0.6, 0.6, and 0.5, respectively^58,59^; therefore, robustness and stability of the GA-MLR model are confirmed based on the obtained statistical performance. The values of 3D and 2D autocorrelations descriptors appearing in model 1 (Eq. 4) are presented in Supplementary Table S4. These descriptors are briefly introduced in Supplementary Table S5. Based on model 1 (Eq. 4), the prominent role of 3D-MoRSE descriptors in combination with 2D autocorrelations can be further evaluated. Minimal multicollinearity between the selected descriptors is confirmed by the VIF index with values less than 3.893 (Table 5); therefore, an informative and optimal GA-MLR model has been built. Based on model 1 (Eq. 4) the predicted values of pIC_50_ for training and test sets are provided in Table 6. Using the same descriptors selected by the GA-MLR model, ANN was also established on 196 compounds as a training set and was validated by the remaining 49 compounds as the test set. The prediction performance confirms the stability and efficiency of ANN:

$${\text{n}}_{{{\text{training}}}} = { 196},{\text{ R}}^{{2}} = \, 0.{642},{\text{ R}}^{{2}}_{{{\text{adj}}}} = \, 0.{61}0,{\text{ RMSE }} = \, 0.{464,}$$

$${\text{n}}_{{{\text{test}}}} = { 49},{\text{ R}}^{{2}} = \, 0.{615},{\text{ RMSEP }} = \, 0.{5}00.$$

**Table 5:** The correlation coefficient of descriptors and corresponding VIF values based on model 1 (Eq. 4).

	Mor12p	RDF010e	Mor14u	Mor15m	GATS6p	Mor19m	Te	G2v	Mor02v	GATS4p	VIF
Mor12p	1.000										2.210
RDF010e	0.142	1.000									3.455
Mor14u	0.097	− 0.155	1.000								1.332
Mor15m	− 0.227	− 0.269	0.053	1.000							1.828
GATS6p	− 0.219	0.057	− 0.147	0.102	1.000						1.388
Mor19m	0.418	0.179	− 0.016	− 0.019	0.298	1.000					1.957
Te	0.384	− 0.193	− 0.172	0.020	− 0.192	− 0.041	1.000				2.885
G2v	− 0.084	0.168	− 0.024	0.170	0.052	0.081	− 0.100	1.000			1.559
Mor02v	− 0.277	− 0.439	− 0.014	− 0.176	0.109	− 0.174	− 0.432	0.197	1.000		3.893
GATS4p	0.184	− 0.083	0.176	− 0.056	− 0.130	− 0.205	0.110	− 0.074	− 0.069	1.000	1.215

**Table 6:** Experimental pIC_50_ values for 245 PI3Kγ inhibitors used as training and test sets and corresponding predicted values for them based on the MLR and ANN methods.

Compound	Activity (pIC_50_)			References
	Exp	Pred		
		MLR	ANN	
1	7.05	8.07	7.82	^31^
2	6.32	7.89	7.95	^31^
3	7.30	7.26	7.19	^31^
**4**^**a**^	7.10	7.68	7.72	^31^
5	6.54	7.47	7.40	^31^
6	7.12	7.26	7.49	^31^
7	6.37	7.79	7.48	^31^
8	8.26	7.90	7.81	^31^
9	7.77	8.09	7.97	^31^
10	7.82	7.55	7.49	^31^
11	7.22	7.92	7.46	^31^
12	8.36	7.95	7.84	^31^
**13**	8.54	7.87	7.79	^31^
14	6.37	7.59	7.89	^31^
15	8.04	7.57	7.32	^31^
16	8.14	7.90	8.05	^31^
**17**	7.85	7.56	7.78	^31^
**18**	7.15	8.09	8.19	^31^
**19**	7.03	8.01	8.40	^31^
20	8.54	8.82	8.78	^31^
21	8.19	8.17	8.17	^31^
22	8.23	7.78	7.93	^31^
23	7.36	8.40	8.08	^31^
24	7.51	8.05	8.25	^31^
25	7.19	7.55	7.86	^31^
26	8.12	7.94	7.38	^31^
27	8.66	8.00	7.94	^31^
28	8.68	8.32	7.95	^31^
**29**	8.66	7.85	7.98	^31^
30	8.89	7.91	7.86	^31^
31	8.57	8.43	8.41	^31^
32	8.18	8.05	8.12	^31^
33	8.10	8.08	8.06	^31^
34	7.28	7.81	7.92	^31^
**35**	9.00	8.01	8.00	^31^
36	8.68	7.27	7.37	^31^
37	8.15	8.22	8.06	^30^
38	8.48	7.51	7.72	^30^
39	8.48	8.57	8.47	^30^
40	7.38	7.64	7.69	^30^
41	8.24	8.16	8.23	^30^
42	7.80	7.46	7.54	^30^
43	8.59	7.75	7.97	^30^
44	8.49	8.33	7.49	^30^
45	6.55	8.21	7.86	^30^
**46**	7.89	7.61	7.70	^30^
47	7.92	8.23	8.40	^30^
48	8.35	8.32	8.36	^30^
49	8.60	8.26	8.24	^30^
50	8.55	8.60	8.55	^30^
51	8.68	8.56	8.41	^30^
**52**	8.80	8.62	8.26	^30^
**53**	8.66	8.56	8.41	^30^
**54**	8.92	8.69	8.47	^30^
55	8.51	8.33	8.51	^30^
56	8.79	8.49	8.60	^30^
57	8.82	8.63	8.77	^30^
58	8.40	8.09	8.21	^30^
59	8.57	7.92	8.08	^30^
60	8.30	8.08	8.17	^30^
**61**	6.74	7.59	7.63	^30^
62	8.59	8.79	8.47	^30^
63	8.30	8.01	8.32	^30^
64	8.22	7.87	7.89	^30^
65	8.17	8.47	8.42	^30^
66	8.05	7.66	7.74	^30^
67	7.30	6.67	7.17	^30^
68	7.59	8.04	7.84	^30^
69	7.55	8.63	8.22	^30^
**70**	7.40	7.18	7.22	^30^
**71**	7.60	6.86	7.49	^30^
72	8.38	8.31	7.67	^30^
73	8.37	8.42	8.28	^30^
74	8.62	8.21	7.64	^30^
**75**	8.43	8.22	8.00	^30^
76	8.62	7.82	8.00	^30^
77	8.10	8.02	8.09	^30^
**78**	8.60	8.04	8.32	^30^
**79**	8.40	8.37	8.46	^30^
80	8.59	8.14	8.27	^30^
**81**	8.40	7.70	7.88	^30^
82	8.82	8.55	8.53	^30^
83	8.92	7.83	8.21	^30^
84	8.55	8.01	8.21	^30^
85	8.59	8.17	8.06	^30^
86	8.48	8.00	8.09	^30^
87	8.68	8.40	8.46	^30^
88	8.48	8.73	8.68	^30^
89	8.44	7.85	7.92	^30^
90	6.07	6.85	6.74	^24^
91	5.24	5.98	6.09	^24^
**92**	5.23	5.65	5.93	^24^
93	7.40	6.31	6.43	^24^
94	6.00	5.90	5.92	^24^
95	6.58	6.85	6.70	^24^
96	7.60	6.92	6.98	^24^
97	7.10	7.47	7.80	^24^
98	6.36	5.99	5.98	^24^
99	7.61	7.51	7.84	^24^
100	8.10	8.46	9.07	^24^
101	5.23	5.72	5.52	^24^
102	6.75	7.58	7.30	^24^
**103**	7.60	6.94	7.04	^24^
104	6.33	7.04	7.10	^24^
105	6.57	6.94	7.04	^24^
**106**	7.00	6.69	6.36	^24^
107	8.40	7.97	8.35	^24^
108	9.00	9.13	8.95	^24^
109	6.09	6.24	6.28	^24^
110	7.22	6.90	6.95	^24^
111	7.22	6.75	6.98	^24^
112	8.19	7.66	7.98	^24^
113	7.30	8.20	7.90	^24^
114	5.33	5.96	5.82	^24^
**115**	5.53	6.17	6.43	^24^
116	5.65	6.52	5.95	^24^
**117**	6.76	6.52	6.20	^24^
**118**	8.08	7.51	8.43	^24^
119	6.00	7.09	6.79	^24^
120	6.10	7.08	6.59	^24^
121	6.29	6.21	6.26	^24^
122	5.52	5.66	5.96	^24^
123	6.17	6.22	6.09	^24^
124	5.70	6.00	5.92	^24^
125	7.60	7.13	7.85	^24^
126	7.60	7.20	7.38	^24^
**127**	8.40	8.15	8.47	^24^
128	8.30	8.18	8.31	^24^
129	8.00	7.43	7.02	^24^
130	8.52	7.80	7.32	^24^
131	7.54	7.19	6.87	^24^
**132**	6.80	7.78	7.38	^24^
**133**	8.90	8.41	8.33	^24^
**134**	8.10	7.57	7.08	^24^
135	5.40	6.49	6.26	^32^
136	6.80	6.22	6.38	^32^
137	6.00	6.00	6.13	^32^
138	7.40	6.09	6.14	^32^
139	6.30	6.48	6.55	^32^
140	6.50	7.19	7.03	^32^
141	6.60	6.39	6.47	^32^
142	6.20	6.45	6.36	^32^
143	6.70	6.98	7.27	^32^
144	6.30	6.77	6.90	^32^
145	6.60	6.23	5.97	^32^
**146**	5.40	6.39	6.13	^32^
147	5.50	5.65	5.75	^32^
148	5.80	6.20	6.07	^32^
149	5.40	6.70	6.43	^32^
150	6.40	6.03	6.05	^32^
151	7.20	6.81	6.49	^32^
152	7.10	6.77	6.69	^32^
**153**	8.10	7.37	7.34	^32^
154	7.00	6.68	6.41	^32^
**155**	6.50	6.75	7.02	^32^
**156**	6.50	6.33	6.39	^32^
**157**	5.50	5.92	6.04	^32^
158	8.20	7.67	8.03	^32^
159	6.20	7.46	7.68	^32^
160	6.60	6.70	6.52	^32^
161	7.89	7.70	7.90	^32^
162	7.50	7.31	7.23	^32^
163	7.80	7.48	7.64	^32^
**164**	6.80	7.08	7.39	^32^
**165**	7.00	7.05	6.83	^32^
166	6.00	6.59	6.38	^32^
167	5.70	6.86	6.63	^32^
168	6.90	6.73	6.87	^32^
169	5.68	6.39	6.27	^18^
**170**	5.64	6.98	6.63	^18^
171	6.72	7.24	7.49	^18^
172	5.52	6.76	6.44	^18^
173	7.57	7.07	6.97	^18^
174	7.60	7.38	7.52	^18^
175	7.55	7.42	7.58	^18^
176	8.24	7.60	7.46	^18^
177	7.38	6.63	6.60	^18^
178	8.52	7.46	7.67	^18^
179	7.42	6.66	6.96	^18^
180	6.72	6.66	6.86	^18^
181	7.16	7.82	7.55	^18^
182	6.96	6.64	7.16	^18^
183	6.00	7.01	7.45	^18^
**184**	7.44	8.27	7.72	^18^
185	7.82	7.62	7.85	^18^
**186**	8.70	8.64	8.76	^18^
187	8.52	8.56	9.00	^18^
188	8.10	7.88	7.89	^18^
189	8.40	7.52	7.62	^18^
190	6.80	6.51	6.38	^18^
191	6.00	7.07	7.45	^18^
192	9.22	8.14	7.85	^18^
193	6.10	6.81	7.22	^18^
194	6.60	6.20	6.13	^18^
195	7.40	6.91	6.92	^29^
**196**	7.22	6.89	6.70	^29^
**197**	6.40	6.58	6.33	^29^
198	6.00	6.37	6.14	^29^
199	6.52	7.09	7.04	^29^
200	6.82	6.92	7.08	^29^
**201**	6.96	6.71	6.69	^29^
202	7.30	6.28	6.36	^29^
203	6.00	6.73	6.68	^29^
204	5.96	6.56	6.51	^29^
**205**	5.96	6.44	6.55	^29^
206	6.48	6.81	6.67	^29^
207	7.85	6.69	6.77	^29^
**208**	7.60	6.72	6.73	^29^
209	6.55	6.96	6.91	^29^
210	6.96	6.95	6.98	^29^
211	6.46	6.86	7.03	^29^
212	6.77	6.49	6.73	^29^
213	6.96	7.12	6.95	^29^
214	7.12	6.74	6.66	^29^
215	6.44	6.96	6.76	^29^
216	7.00	6.54	6.61	^29^
217	7.10	7.22	6.92	^41^
218	7.90	7.88	7.72	^41^
**219**	6.90	7.82	7.99	^41^
220	6.90	7.81	7.31	^41^
221	7.80	7.78	7.57	^41^
222	6.60	7.38	7.23	^41^
223	7.20	7.53	7.22	^41^
**224**	7.50	7.07	6.66	^41^
225	7.60	8.18	7.86	^41^
226	7.60	7.78	7.43	^41^
227	8.10	7.76	7.56	^41^
228	8.20	8.13	7.94	^41^
229	8.00	8.22	8.13	^41^
**230**	8.60	8.43	8.37	^41^
231	7.20	8.18	7.88	^41^
**232**	8.50	7.30	7.21	^41^
233	7.80	7.74	7.73	^41^
234	8.20	8.37	7.99	^41^
235	8.40	8.24	8.34	^41^
236	8.60	7.90	7.44	^41^
237	7.70	7.23	7.32	^28^
238	7.60	6.91	7.41	^28^
**239**	7.50	7.45	7.91	^28^
240	7.70	7.44	7.48	^28^
241	7.70	8.10	7.72	^28^
242	7.40	7.37	7.87	^28^
243	8.10	7.07	7.40	^28^
244	7.60	7.86	7.89	^28^
**245**	7.80	7.81	8.03	^28^

^a^Bold cases used as a test set.

The performance of ANN is relatively better than MLR, in the case of the training set (R^2^_train_ = 0.642 for ANN in comparison to R^2^_train_ = 0.623 for MLR); conversely, in the case of the test set, the MLR has the better prediction performance (R^2^_test_ = 0.662 for MLR in compare to ANN with R^2^_train_ = 0.615). The calculated values of pIC_50_ for training and test sets using of ANN technique can be seen in Table 6.

### Out-of-sample testing validation

We carried out the out-of-sample testing, as a validation method, to indicate the robustness and stability of the model and to show that the test set selected by the DUPLEX algorithm is representative. Using Minitab software, 49 molecules were selected randomly as a test set from the data set (245 molecules); then the QSAR model was established on the 196 remaining compounds. This model was employed to predict the inhibitory activity of the test set.

The above-mentioned process was repeated 10 times. The results were presented in Table 7; including R^2^ of training and test sets and VIF. These results are in good agreement with the accepted values for these parameters except for the fifth iteration (in this case the R^2^ value is slightly less than 0.5 for the test set). These results also confirm that the descriptors are relevant and model 1 (Eq. 4) is predictive. Also, the maximum value obtained for the VIF parameter at each time of out-of-sample testing validation is less than 5, so the established models are not involved with multicollinearity error. The test compounds that were selected randomly at each time of the aforementioned validation method are presented in Supplementary Table S6. In Table 8, the total 56 descriptors selected by GA are listed and descriptors with the highest frequency of iterations in the established models were bold.

**Table 7:** Out-of-sample testing validation results based on the random dataset splitting.

Iteration	GA-MLR models developed on the training set (196 molecules) which were selected randomly by Minitab	Number of descriptors included in the model	Max. VIF value	R^2^	
				Training set	Test set
1	− 4.584 + 0.205 × RDF010e + 1.196 × Mor32p − 0.924 × MATS7p + 3.122 × ATS1m − 0.044 × RDF035e + 4.589 × G3v + 0.935 × Mor18p + 0.801 × GATS4p − 1.015 × GATS2e	9	3.514	0.612	0.567
2	8.065–0.875 × Mor12p + 0.227 × RDF010e − 0.406 × Mor14u − 0.512 × Mor15m − 0.966 × MATS7p − 12.374 × G2v − 1.31 × MATS4p − 1.001 × Mor19m − 0.531 × Mor17p + 0.017 × Te	10	3.640	0.649	0.519
	8.213–0.829 × Mor12p + 0.225 × RDF010e − 0.378 × Mor14u − 0.529 × Mor15m − 0.852 × MATS7p − 12.482 × G2v − 1.238 × MATS4p − 1.162 × Mor19m − 0.504 × Mor17p + 0.022 × Te − 0.81 × GATS6p + 3.491 × G3v	12	3.720	0.671	0.573
3	4.73–1.174 × Mor14p + 0.075 × RDF040m − 1.179 × MATS7p + 4.615 × G3v + 0.188 × RDF010e + 1.588 × Mor18p + 0.044 × Tm − 0.618 × Mor17p − 0.392 × Mor15m + 0.019 × RDF070e	10	3.770	0.655	0.546
4	7.584–0.889 × Mor12p + 0.056 × RDF115p − 0.554 × Mor14u − 0.91 × Mor19m − 1.721 × GATS6p + 0.03 × Te − 0.416 × Mor15m + 0.222 × RDF010e + 0.782 × GATS4p − 0.038 × Mor02v	10	4.071	0.629	0.618
	9.338–0.723 × Mor12p − 0.454 × Mor14u − 1.089 × Mor19m − 1.62 × GATS6p + 0.039 × Te − 0.393 × Mor15m + 0.216 × RDF010e + 0.716 × GATS4p − 0.054 × Mor02v − 11.137 × G2v − 0.586 × Mor17p + 0.81 × MATS2e	12	4.256	0.659	0.663
5	11.308–0.617 × Mor12p + 0.087 × RDF030p − 1.182 × GATS6p − 0.842 × Mor19m − 0.84 × GATS2e + 1.105 × GATS4p + 1.611 × Mor32p − 11.84 × G2e − 0.939 × MATS7v − 10.216 × G2v	10	2.749	0.620	0.455
6	7.637–0.701 × Mor12p + 0.264 × RDF010e − 0.544 × Mor14u − 0.927 × Mor19m − 1.528 × GATS6p − 0.423 × Mor15m + 0.042 × Te − 0.582 × Mor17p − 0.045 × Mor02v	9	4.602	0.647	0.512
7	9.063–0.939 × Mor12p + 0.273 × RDF010e − 0.552 × Mor14u − 0.549 × Mor15m − 0.838 × MATS7p − 1.172 × MATS4p − 1.096 × Mor19m − 1.237 × GATS6v − 10.483 × G2v + 0.017 × Te	10	2.685	0.611	0.715
	9.673–0.867 × Mor12p + 0.33 × RDF010e − 0.573 × Mor14u − 0.486 × Mor15m − 0.605 × MATS7p − 1.056 × MATS4p − 1.081 × Mor19m − 1.432 × GATS6v − 11.274 × G2v + 0.03 × Te − 0.045 × Mor02v	11	4.249	0.627	0.659
8	7.677–0.79 × Mor12p − 0.93 × Mor19m − 0.973 × GATS6p + 1.183 × GATS4p − 1.242 × MATS7p − 10.824 × G2v + 1.735 × Mor32p + 0.026 × Tm − 0.419 × Mor14u + 0.143 × RDF010e	10	2.728	0.630	0.567
	8.293–0.753 × Mor12p − 0.909 × Mor19m − 1.255 × GATS6p + 1.171 × GATS4p − 0.949 × MATS7p − 11.963 × G2v + 1.19 × Mor32p + 0.028 × Tm − 0.461 × Mor14u + 0.192 × RDF010e − 0.382 × Mor15m	11	2.826	0.650	0.603
9	5.887–0.895 × Mor12p + 0.211 × RDF010e − 0.526 × Mor14u − 0.959 × MATS7p − 0.362 × Mor15m + 0.032 × Tm − 0.945 × GATS6p − 0.939 × Mor19m + 0.762 × GATS4p − 0.433 × Mor17p	10	3.298	0.635	0.597
	8.017–0.775 × Mor12p + 0.23 × RDF010e − 0.493 × Mor14u − 0.834 × MATS7p − 0.339 × Mor15m + 0.043 × Tm − 0.987 × GATS6p − 0.955 × Mor19m + 0.763 × GATS4p − 0.575 × Mor17p − 0.04 × Mor02v − 9.722 × G2v	12	4.175	0.658	0.642
10	− 4.785 + 0.293 × RDF010e − 1.202 × MATS7p − 0.445 × Mor15m + 1.155 × GATS4p + 3.047 × ATS1m + 1.385 × Mor18p − 0.744 × Mor14p + 5.133 × G3m − 1.147 × GATS2e − 0.026 × RDF035e	10	4.900	0.642	0.566

**Table 8:** The total 56 descriptors selected by GA carried out to out-of-sample testing validation.

Molecular descriptors	Descriptor category
ATS1m, MATS7v, MATS2e, MATS4p, **MATS7p** (6 times), MATS8p, GATS7m, GATS2v, GATS6v, GATS2e, GATS8e, **GATS4p** (5 times), **GATS6p** (7 times)	2D autocorrelations
RDF050u, RDF040m, RDF050m, RDF070v, **RDF010e** (9 times), RDF015e, RDF020e, RDF035e, RDF045e, RDF050e, RDF070e, RDF020p, RDF030p, RDF115p,	RDF descriptors
Mor12u, **Mor14u** (6 times), Mor17u, Mor26u, **Mor15m** (8 times), **Mor19m** (7 times), Mor23m, Mor30m, Mor32m, **Mor02v** (4 times), Mor19v, Mor03e, Mor02p, Mor03p, Mor10p, **Mor12p** (8 times), Mor14p, **Mor17p** (5 times), Mor18p, Mor19p, Mor32p,	3D-MoRSE descriptors
**G2v** (6 times), **G3v** (5 times), G2e, G2p, E3e, Tm, **Te** (5 times)	WHIM descriptors

The most frequent descriptors, also included in model 1 (Eq. 4), are in bold.

A notable point is that frequent descriptors in the models established based on this validation method are also included in model 1 (Eq. 4). Since model 1 (Eq. 4), is well confirmed by the recent validation, the methodology used for QSAR modeling can be considered valid; especially, in the case of feature selection by GA and data splitting by duplex algorithm. Statistical performance parameters represented in Table 7, also verify that model 1 (Eq. 4) is not involved with overfitting problem^60,61^.

Subsequently, the GA-MLR model was developed using whole 245 compounds (no splitting) for complementary evaluations of the model.

5 $$\begin{aligned} {\text{pIC}}_{{{5}0}} & = { 9}.{4}0{2 }\left( { \pm 0.{829}} \right) \, - \, 0.{933 }\left( { \pm 0.{141}} \right){\text{Mor12p}} + \, 0.{27}0 \, \left( { \pm 0.0{41}} \right){\text{RDF}}0{1}0{\text{e}} - \, 0.{589 }\left( { \pm 0.{1}0{3}} \right){\text{Mor14u}} \\ & \quad - \, 0.{536 }\left( { \pm 0.0{98}} \right){\text{Mor15m}} - { 1}.{728 }\left( { \pm 0.{269}} \right){\text{GATS6p}} - \, 0.{81}0 \, \left( { \pm 0.{167}} \right){\text{Mor19m}} \\ & \quad + \, 0.0{36}\left( { \pm 0.00{7}} \right){\text{Te}} + \, 0.{77}0 \, \left( { \pm 0.{264}} \right){\text{GATS4p}} - {11}.{635 }\left( { \pm {3}.{658}} \right){\text{G2v}} - 0.0{39 }\left( { \pm 0.0{13}} \right){\text{Mor}}0{\text{2v}} \\ \end{aligned}$$

$$\begin{aligned} & {\text{n }} = { 245},{\text{ R}}^{{2}} = \, 0.{632},{\text{ R}}^{{2}}_{{{\text{adj}}}} = \, 0.{616},{\text{ Q}}^{{2}}_{{{\text{LOO}}}} = \, 0.{6}00,{\text{ RMSE }} = \, 0.{476},{\text{ F }} = { 4}0.{114}, \, \\ & \quad {\text{RMSE}}_{{{\text{CV}}}} = \, 0.{623},{\text{R}}^{{2}}_{{{\text{y}} - {\text{random}}}} = \, 0.0{11},{\text{Q}}^{{2}}_{{{\text{y}} - {\text{random}}}} = \, 0.000{6},{\text{PRESS }} = { 94}.{677}{\text{.}} \\ \end{aligned}$$

### External verification of the QSAR modes

In order to, first, further evaluate the model robustness, second, to investigate the application domain of the models, and, third, to compare the effectiveness of the models in the face of novel structures, a diverse set of PI3Kγ inhibitors consisting of 45 compounds, out of the training and test sets^17,25,33,62–65^ (Supplementary Table S7), were used to external verification of the predictive QSAR models. SMILES strings of these molecules are presented in Supplementary Table S8. External verification was carried out based on the following process: first, by considering model 1 (Eq. 4), the corresponding descriptors were extracted for each structure of the validation set (Supplementary Table S9). Then, predicted pIC_50_ values of these compounds were calculated through the generalization of the MLR and ANN models to them (Table 9).

**Table 9:** The experimental pIC_50_ values of 45 PI3Kγ inhibitors used as a validation set and corresponding predicted values for them based on the MLR and ANN methods.

Compound	Activity (pIC_50_)			References
	Exp	Pred		
		MLR	ANN	
246	7.01	7.86	6.98	^62^
247	7.43	8.21	7.63	^62^
248	7.20	8.27	7.38	^62^
249	7.82	8.47	8.64	^62^
250	7.77	7.80	7.70	^62^
251	9.30	8.02	8.07	^62^
252	8.15	7.96	7.95	^62^
253	9.15	7.98	8.37	^62^
254	9.15	8.28	8.05	^62^
255	7.12	6.97	6.91	^62^
256	7.85	7.03	7.05	^62^
257	7.85	7.48	7.83	^63^
258	7.32	6.76	6.58	^63^
259	7.44	7.07	7.40	^63^
260	6.51	6.25	6.56	^63^
261	7.80	7.38	7.58	^63^
262	9.00	7.80	8.41	^63^
263	5.98	6.83	6.33	^63^
264	7.80	7.79	8.31	^63^
265	6.80	5.94	5.71	^63^
266	7.30	7.27	7.30	^63^
267	7.60	7.11	7.14	^63^
268	5.51	6.46	6.13	^63^
269	6.80	7.53	7.28	^64^
270	7.60	7.82	7.95	^64^
271	8.10	8.25	8.44	^64^
272	7.20	7.13	6.93	^64^
273	9.10	7.91	8.10	^64^
274	8.90	8.13	8.32	^64^
275	9.10	7.55	7.96	^64^
276	8.90	7.83	7.90	^64^
277	9.00	8.29	8.62	^64^
278	6.30	7.40	7.47	^65^
279	5.20	6.62	6.78	^65^
280	7.60	6.14	6.40	^65^
281	7.55	7.77	7.95	^65^
282	5.00	5.83	5.53	^65^
283	5.72	6.12	6.37	^65^
284	6.79	6.31	6.31	^17^
285	6.42	5.96	5.63	^17^
286	5.89	6.06	5.78	^17^
287	7.68	6.70	6.84	^33^
288	8.70	8.06	8.37	^33^
289	7.20	7.16	7.49	^25^
290	7.30	6.92	6.69	^25^

### Simultaneous comparison of training, test, and validation sets based on the QSAR analysis results

Displaying the training, test, and validation sets in one graphical plot provides a clear insight into the molecular distribution, goodness of fit in three subsets of PI3Kγ inhibitors, and ultimately, a more accurate assessment of the application domain of the models. In the following, each of these three aspects is explained in detail.

### Data set distribution in terms of standard deviation

**Figure 2:**
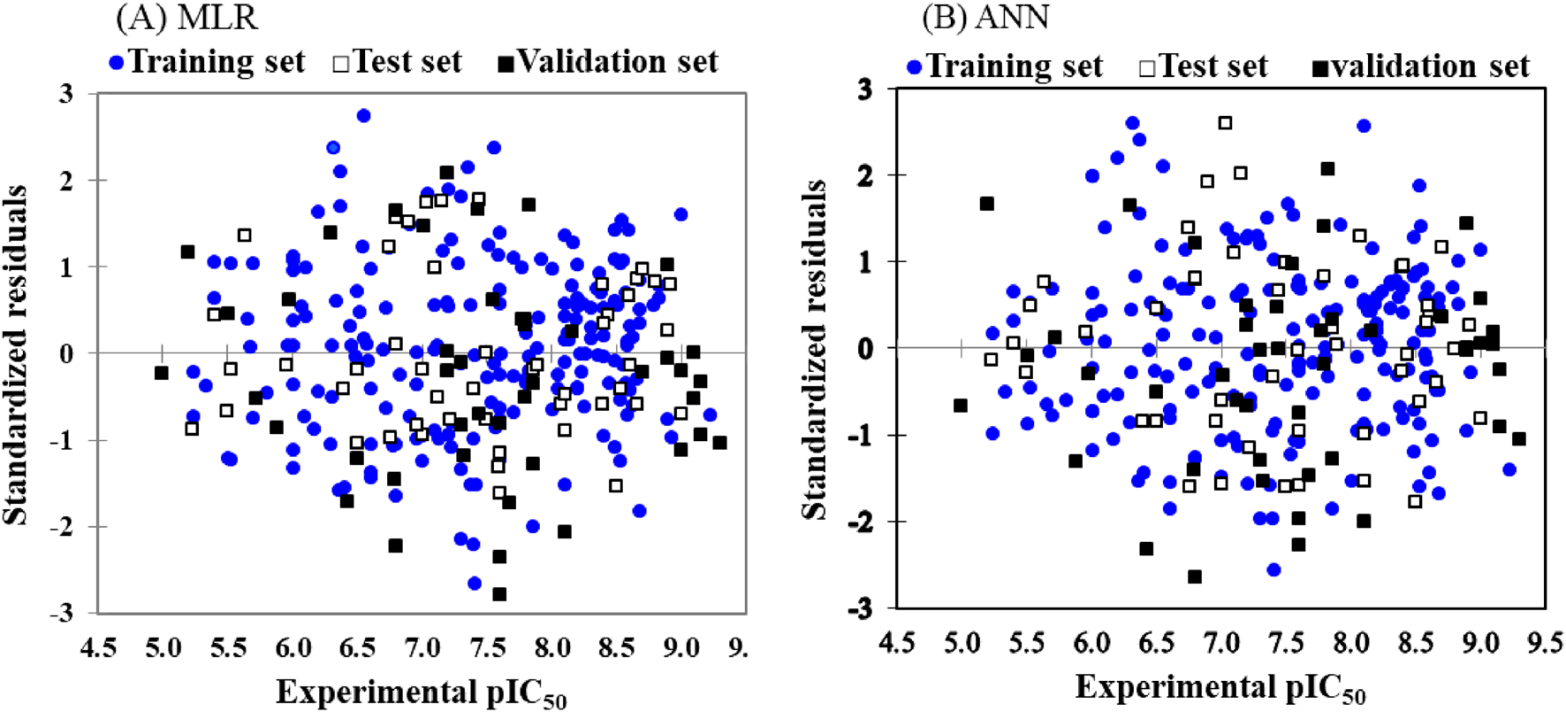
Dispersion plots of standardized residuals versus experimental values of the pIC_50_ during the QSAR model development on PI3Kγ inhibitors.

Based on the MLR and ANN models, standard deviation ((pIC_50_)_Exp_− (pIC_50_) _pred_) of PI3Kγ inhibitors versus their corresponding (pIC_50_)_Exp_ values have been displayed in Fig. 2. The random and uniform distribution of the data on both sides of standard deviation equal to zero can be seen not only in the training set but also in the test and validation sets as a reliable representative of the entire data set. These results confirm that the systematic error did not occur during the model development.

### Regression results of the validation set in comparison to the training and test sets

**Figure 3:**
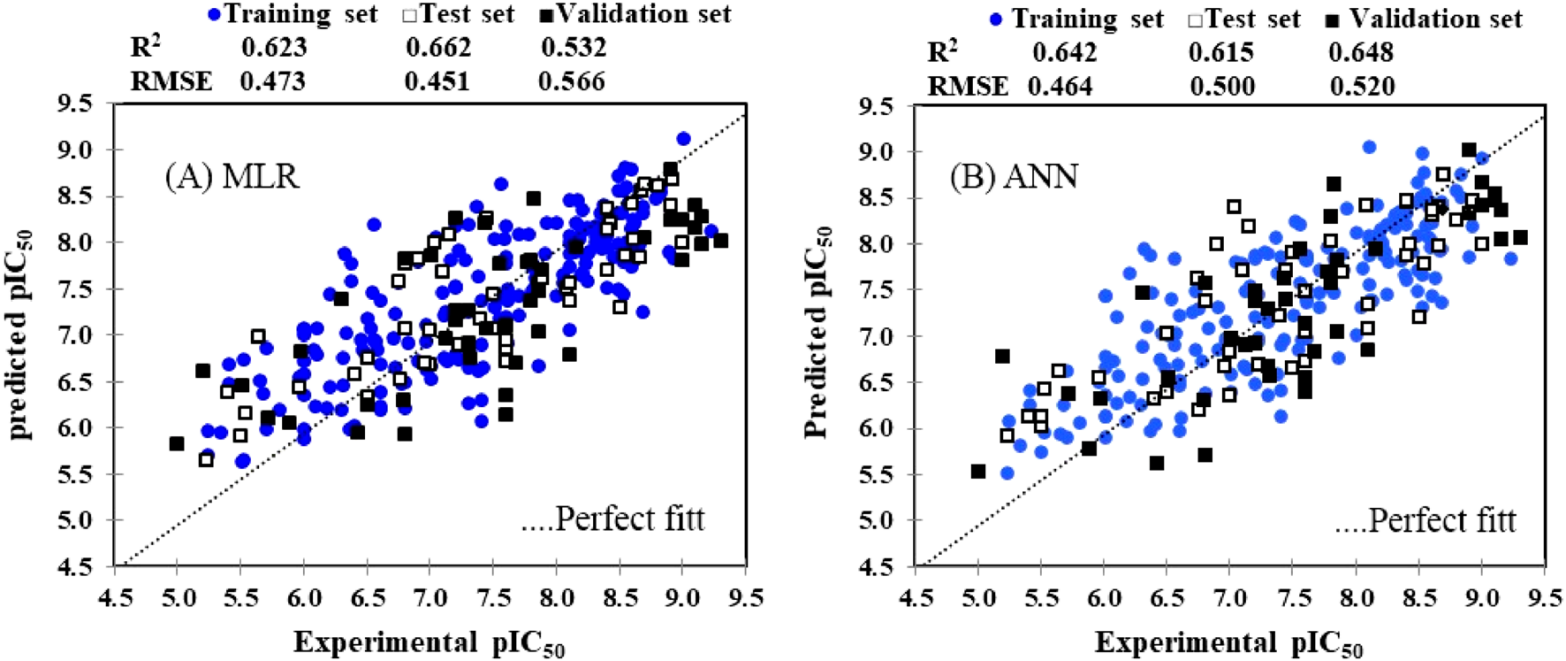
Scatter plots of the predicted versus experimental pIC_50_ values for MLR (**A**) and ANN (**B**) models constructed on PI3Kγ inhibitory activity.

Modeling efficiency has been evaluated based on the training and test sets in section “QSAR modeling results”; here, we will focus on the model evaluation based on the validation set. A scatter plot of the predicted pIC_50_ versus the experimental values during the QSAR model development on 196 PI3Kγ inhibitors has been displayed in Fig. 3. Based on these observations, both proposed models have good predictive performance; nevertheless, ANN is superior to MLR in the face of the validation set. (R^2^_valid._ = 0.648 for ANN in comparison to R^2^_valid._ = 0.532 for MLR). Since ANN is a nonlinear modeling algorithm, considered to be more efficient with high flexibility. Also, using three methods for data splitting, the performance statistical parameters were obtained as: DUPLEX algorithm (R^2^ = 0.532, RMSE = 0.566), Kennard–Stone algorithm, (R^2^ = 0.552, RMSE = 0.571) and random data splitting (R^2^ = 0.532, RMSE = 0.517). Based on the following reasons both models, especially ANN, are robust and approved:

1. The wide variety of structures with pIC_50_ from 5.00 to 9.30 was used in the validation process
2. QSAR models established on the small number of compounds tend to have better prediction performance than the models developed on a large data set. On the other hand, the efficiency of the QSAR model built from the large data set, consisting of diverse chemical structures and a wide range of pIC_50_, may seem low due to confounding factors^66^. However, a model that has been established on more compounds may have a wider applicability domain^67^ which will be described in the next section.

### Determination of the application domain of the model

**Figure 4:**
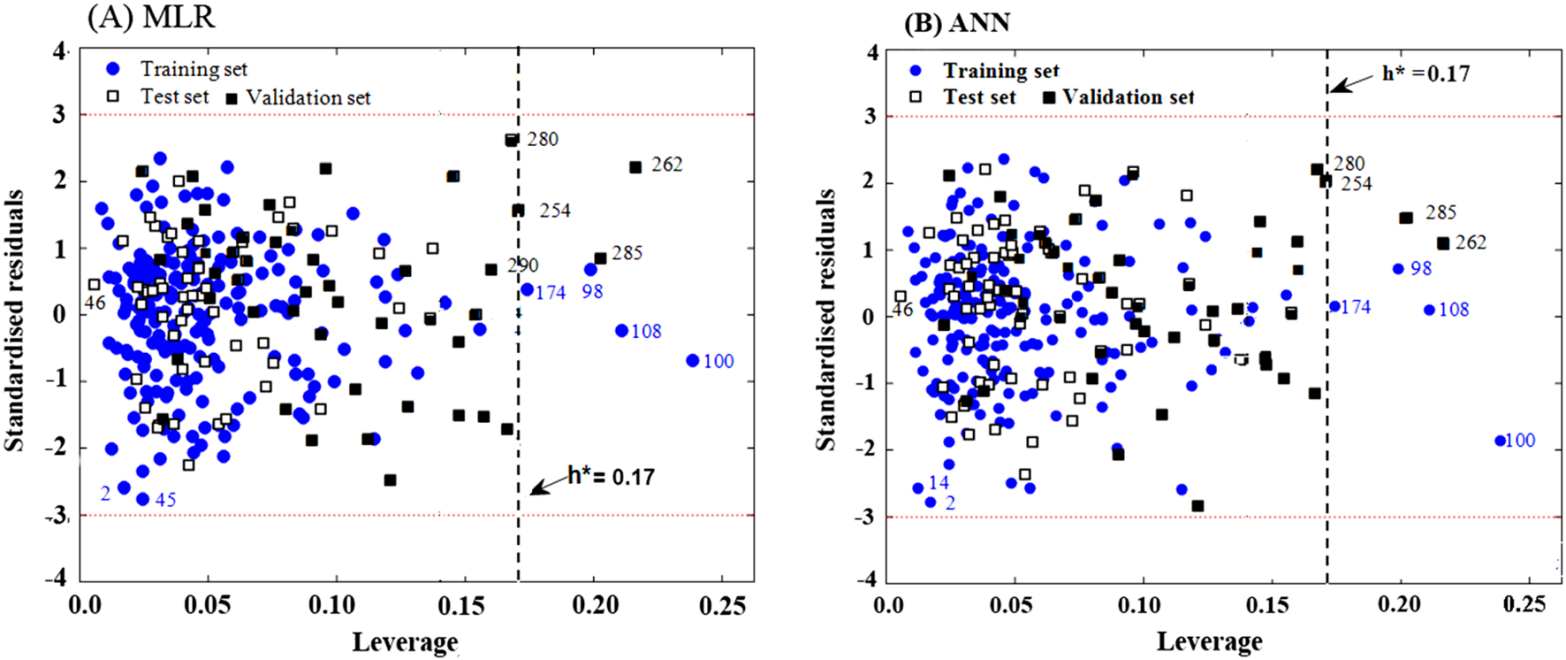
Williams plots-based analyses to compare training, test, and validation sets during the MLR and ANN models development on the PI3Kγ inhibitory.

One of the main aspects of QSAR modeling is determining the application domain (AD) of the model. AD is defined as a chemical space constructed by the descriptors and biological responses used in QSAR model development on the training set. Using this approach, the model efficiency in the face of new compounds that may have not been synthesized is assessed. Williams plot-based analysis was used to determine AD. Williams plots represented in Fig. 4 are based on the MLR and ANN model results. This figure makes it possible to comparison the validation set with the training and test set simultaneously in terms of structural similarity and inhibitory activity.

Acceptable limits of structural similarity and inhibitory activity, have been marked with vertical dash line (warning leverage) and horizontal dotted lines, respectively. It can be observed that all of the 290 compounds, consisting of 196, 49, and 45 molecules as training, test, and validation set respectively, are within the boundaries of acceptable standard deviation (± 3δ). To get a better insight from the structural similarity and biological activity in Fig. 4, the molecules close to the boundaries, have been also specified by their corresponding numbers. A chemical structure with high leverage (h > h*) in the training has high influences ability in the modeling process, thus the chemical in the training set is not an outlier for the response fitting. h* is calculated as follow:

6 $${h}^{*}=3\frac{(K+1)}{n}.$$

n and k are the numbers of training compounds and descriptors in the model, respectively.

None of the compounds belonging to the test set is *X* outlier (h* = 0.17). However, two molecules that belong to the validation set are out of the structural similarity threshold. In interpreting these observations, the following explanations can be noted:

1. The whole molecules of the test set are placed in the acceptable limits of structural similarity and standard deviation; it may be because that the models are developed based on the samples with a wide range of structures and pIC_50_. The proposed models with wide application domine can predict the test set with credibility.
2. The four molecules of the training set with a leverage value greater than 0.17, means that these compounds are very dominant in determining the model; in other words, they are good “influence points” and can be indicators of high accuracy and robustness of the model^68^. Moreover, these compounds have been accurately predicted by model 1 (Eq. 4) with the lowest standard deviation.
3. In the case of the validation set, as can be seen, only two compounds are *X* outlier; however, it should be noted that in the routine QSAR studies, evaluation is limited to the test set but in this research, for further assessment of the models, a wide variety of structures were employed as a validation set, out of the training and test sets. As shown in Fig. 4, two *X* outlier compounds of the validation set are also located on the left side of compound 100 with lower leverage than it. Therefore, according to the description provided in “Materials and methods”, the predicted results of these two compounds can be also accepted. In addition, these molecules are also well predicted by model 1 (Eq. 4). Accordingly, the high stability, predictive ability, and robustness of the MLR and ANN models were assessed in the face of new compounds.

### Descriptors interpretation

In-depth insight about structural descriptors helps chemists in the design of new effective drugs through the interpretation of QSAR models. For example, several factors are involved in the binding of a ligand to a target such as van der Waals volumes and surfaces, polarizability, hydrophobicity, lipophobicity, etc. Four categories of descriptors which are entered in the models are defined in Supplementary Table S5 and briefly described here:

### Characteristics and capabilities of the four different categories of descriptors

#### 3D-MoRSE descriptors

This category of descriptors was introduced in 1996 by Schuur and et al.^69^. Range of scattering parameter values (0–31 Å^−1^) and variety of weighting schemes (unweighted and weighted with atomic mass, atomic van der Waals volume, atomic Sanderson electronegativity, and atomic polarizability) has given them high flexibility and pervasively. 3D-MoRSE descriptors can be employed successfully to extract information from the entire structure of the molecule that results to discriminate a large and diverse set of compounds correctly. These descriptors are sensitive to the presence of specific molecular fragments.

Based on the coordinates, 3D structure, and electron diffraction of molecules, 3D-MoRSE descriptors provide information that calculates by summing atom weights as the following expression:

7 $$\mathrm{I}\left(\mathrm{s}\right)=\sum_{\mathrm{i}=2}^{\mathrm{N}}\sum_{\mathrm{j}=1}^{\mathrm{i}-1}{\mathrm{A}}_{\mathrm{i}}{\mathrm{A}}_{\mathrm{j}}\frac{{\mathrm{sinsr}}_{\mathrm{ij}}}{{\mathrm{sr}}_{\mathrm{ij}}},$$

where I is diffraction intensity of electron diffraction, s is the scattering parameter (Angle X-ray scattering), r_ij_ is the interatomic distance between *i*-th and *j*-th atoms, N indicates the number of atoms, and A_i_ and A_j_ expressed the different atomic properties as the weight that were mentioned above. The wide range of scattering parameters is calculated at 32 evenly distributed values at scattering angle(s) in the range of 0–31 Å^−1^ from the 3D atomic coordinates of molecules based on the above function.

#### RDF descriptors

In this category, molecular descriptors are calculated through radial basis functions centered on different interatomic distances and are based on the probability of finding an atom in interatomic space with an r radius^70^. In this category, atoms can also be weighted by various atomic properties (atomic mass, polarizability, etc.). They are independent of factors such as the size of a molecule that is dependent on the number of atoms and focus on describing the 3D arrangement of atoms. These descriptors are effective in providing properties that refer to the morphology of molecular such as steric hindrance, planar or non-planar structure, etc. Another feature of these descriptors which makes them a suitable choice for QSAR analysis is that they are invariant against translation and rotation of a molecule.

#### WHIM descriptors

These descriptors as statistical indexes are obtained through the projection of atoms on the Cartesian coordinate^71^. To calculate them, the most stable conformer with minimum energy is used. They can cover 3D information about different characteristics of molecular structure such as size, shape, symmetry, and atomic distribution. Specific information can be obtained from any subset of these descriptors.

#### 2D autocorrelation descriptors

2D autocorrelation descriptors are calculated based on the molecular graph to represent the topological structure of the compounds^72^. In this class of descriptors, interatomic topology distance is considered based on the length of the types of atomic pairs. Atoms are visualized as the set of discrete points in space and atomic properties including atomic masses, atomic van der Waals volumes, atomic Sanderson electronegativities, and atomic polarizabilities were used to evaluate at that points^73^. This class of descriptors in combination with 3D-MoRSE descriptors, discuss chemical space between the compounds^74^. Depending on they are unweighted or weighted with atomic mass, atomic van der Waals volume, atomic Sanderson electronegativity, and atomic polarizability provide a wide variety of information.

### Interpretation of the type and coefficient of descriptors in the model compared with X-ray structures of target-ligand complexes

It is noteworthy that in the models established in this research, 3D-MoRSE descriptors have a prominent presence. The negative sign of the coefficients for descriptors weighted with van der Waals volume (G2v and Mor02v) and the positive sign of the coefficients for descriptors weighted with atomic Sanderson electronegativity (RDF010e and Te) is favorable for increasing the inhibitory activities of molecules. The negative sign of the coefficients for Mor12p and GATS6p that were weighted with atomic polarizability, show that smaller or negative values for these descriptors are favorable for increasing the activities of molecules. In the case of descriptors weighted with atomic mass (Mor15m and Mor19m), the negative sign of coefficients is favorable for increasing the inhibitory activities of molecules.

These results, clearly are in good agreement with the experimental observations. This claim is confirmed through an investigation of the X-ray crystal structure which is used following the experimental assays to demonstrate the binding of the ligand with the target site (ATP-binding pocket) of the PI3Kγ enzyme^18,24^. Interaction between drug structures and PI3Kγ enzyme occurs through strongly electronegative atoms such as N, O, or F. These atoms in the role of hydrogen bond acceptor or hydrogen bond donor (NH and OH) bind to the corresponding polar group R of residual amino acids including Aspartic acid, Glycine, Glutamine, Tryptophan, Lysine, Serine and so on.

Based on the empirical observations, there is a direct relationship between the polarity of the compounds and their inhibitory activity. In a similar situation, the type and coefficients of the descriptors present in the models, show that by increasing the electronegative atoms (N, O, or F) in the structures, their inhibitory activity is enhanced.

Devinyak et al.^75^ reported that in 3D-MoRSE descriptors weighted by atomic van der Waals volume, and atomic polarizability, significantly decreases the effect of Hydrogen and diminishes the roles of Nitrogen, Oxygen, and Fluorine. In other words, the presence of Oxygen and Nitrogen atoms in the structures reduces the values of these descriptors. Since these descriptors have negative-sign coefficients in the model, smaller values for them are favorable and lead to an increase in their inhibitory activity.

They also showed that 3D-MoRSE descriptors weighted by atomic mass, practically eliminate the role of Hydrogen atoms, while significantly increasing the effect of Phosphorus, Sulfur, and Chlorine on the values of these descriptors. Considering the negative sign of coefficients for this class of descriptors entered in our developed model, larger values of them, in agreement with the experimental observations, lead to the decrease of drug inhibitory activity.

In the case of RDF010e and two other descriptors that were weighted by atomic Sanderson electronegativity coefficients in the models have a positive sign. The presence of the electronegative atoms such as N, O, F, or Cl in the structure of these compounds, increases the value of the aforementioned descriptors; consequently, leads to an increase in the predicted inhibitory activity. In agreement with the empirical observations, these results confirm the stability and correctness of the models, again.

Based on the above interpretation, the total descriptors used in the modeling are in good agreement with the experimental results except for GATS4p. This descriptor was the last choice in the stepwise modeling approach with SPSS software and has the least influence in prediction activity. Elimination of GATS4p has no significant effect on the predictive ability of the models.

Altogether, polar regions strengthen the inhibitory activity of the molecules used as inhibitors of PI3Kγ enzyme. while hydrophobic substitution such as bulky groups and long carbon chain substitution weaken it. Comparing the structures presented in Supplementary Table S1 with experimental activities and modeling results gives a better impression of these structures. For example, the pairs or the series of following compounds can be compared: the series 107, 108 and 109, the pairs 96 and 97, 102 and 103, 191 and 192, 193 and 194, 202 and 203, 209 and 228, 229 and 230, 167 and 168, and so on.

Based on the above discussions the stability and efficiency of the QSAR model were confirmed; so, it can be employed in the face of the external structures in the application domain of the model.

## Conclusion

These compounds were previously confirmed as selective isoform-specific PI3Kγ inhibitors by X-ray crystallography. Drug-likeness of them was also confirmed well, based on Lipinski’s rule of five, before using them in the modeling process. QSAR analysis and its evaluation were performed on a diverse set of PI3Kγ inhibitors in a wide range of pIC_50_ using MLR and ANN models. The out-of-sample testing, as a validation method, was carried out 10 times with different test set selected randomly. The results indicate that descriptors are relevant, the model is predictive, and not facing overfitting. The models were assessed also successfully using another set of compounds out of training and test sets with various structures. To further evaluate the robustness and interpretability of the models and to ensure the accuracy of the methodology used in the modeling process, these models were interpreted based on the type and coefficients of the descriptors included in the models. Results are in good agreement with X-ray structures of target-ligand complexes.

## Supplementary Information

Supplementary Information.

## References

[1] Fruman DA (2017). The PI3K pathway in human disease. Cell.

[2] Toker A, Cantley LC (1997). Signalling through the lipid products of phosphoinositide-3-OH kinase. Nature.

[3] Lewis J, Raff M, Roberts K (2002). Molecular biology of the cell (4th Ed). J. Biol. Educ..

[4] Hirsch E (2000). Central role for G protein coupled PI3Kgamma in inflammation. Science.

[5] Wymann MP, Zvelebil M, Laffargue M (2003). Phosphoinositide 3-kinase signalling—Which way to target?. Trends Pharmacol. Sci..

[6] Vanhaesebroeck B, Guillermet-Guibert J, Graupera M, Bilanges B (2010). The emerging mechanisms of isoform-specific PI3K signalling. Nat. Rev. Mol. Cell Biol..

[7] Cantley LC (2002). The phosphoinositide 3-kinase pathway. Science.

[8] Hawkins PT, Anderson KE, Davidson K, Stephens LR (2006). Signalling through Class I PI3Ks in mammalian cells. Biochem. Soc. Trans..

[9] Fruman DA, Rommel C (2014). PI3K and cancer: Lessons, challenges and opportunities. Nat. Rev. Drug Discov..

[10] Vivanco I, Sawyers CL (2002). The phosphatidylinositol 3-kinase-AKT pathway in humancancer. Nat. Rev. Cancer.

[11] Brader S, Eccles SA (2004). Phosphoinositide 3-kinase signalling pathways in tumor progression, invasion and angiogenesis. Tumori.

[12] Katso R, Okkenhaug K, Ahmadi K, Timms J, Waterfield MD (2001). Cellular function of phosphoinositide 3-kinases: Implications for development, homeostasis, and cancer. Annu. Rev. Cell Dev. Biol..

[13] Engelman JA, Luo J, Cantley LC (2006). The evolution of phosphatidylinositol 3-kinases as regulators of growth and metabolism. Nat. Rev. Genet..

[14] Porcu P (2014). Clinical activity of duvelisib (IPI-145), a phosphoinositide- 3-kinase-δ, γ inhibitor, in patients previously treated with ibrutinib. Blood.

[15] Hancox U (2015). Inhibition of PI3Kβ signaling with AZD8186 inhibits growth of PTEN-deficient breast and prostate tumors alone and in combination with docetaxel. Mol. Cancer Ther..

[16] Okkenhaug K, Graupera M, Vanhaesebroeck B (2016). Targeting PI3K in cancer: Impact on tumor cells, their protective stroma, angiogenesis, and immunotherapy. Cancer Discov..

[17] Williams O (2010). Discovery of dual inhibitors of the immune cell PI3Ks p110δ and p110γ: a prototype for new anti-inflammatory drugs. Chem. Biol..

[18] Perry MWD (2019). Evolution of PI3Kγ and δ inhibitors for inflammatory and autoimmune diseases. J. Med. Chem..

[19] D’Angelo ND (2011). Discovery and optimization of a series of benzothiazole phosphoinositide 3-kinase (PI3K)/mammalian target of rapamycin (mTOR) dual inhibitors. J. Med. Chem..

[20] Pujala B (2016). Discovery of pyrazolopyrimidine derivatives as novel dual inhibitors of BTK and PI3Kδ. ACS Med. Chem. Lett..

[21] Kaneda MM (2016). PI3Kγ 3 is a molecular switch that controls immune suppression. Nature.

[22] Stark AK, Sriskantharajah S, Hessel EM, Okkenhaug K (2015). PI3K inhibitors in inflammation, autoimmunity and cancer. Curr. Opin. Pharmacol..

[23] Ardito F, Giuliani M, Perrone D, Troiano G, Muzio LL (2017). The crucial role of protein phosphorylation in cell signaling and its use as targeted therapy (review). Int. J. Mol. Med..

[24] Garces AE, Stocks MJ (2019). Class 1 PI3K clinical candidates and recent inhibitor design strategies: A medicinal chemistry perspective. J. Med. Chem..

[25] Gangadhara G (2019). A class of highly selective inhibitors bind to an active state of PI3Kγ. Nat. Chem. Biol..

[26] Come JH (2018). Design and synthesis of a novel series of orally bioavailable, CNS-penetrant, isoform selective phosphoinositide 3-kinase γ (PI3Kγ) inhibitors with potential for the treatment of multiple sclerosis (MS). J. Med. Chem..

[27] Collier PN (2015). Structural basis for isoform selectivity in a class of benzothiazole inhibitors of phosphoinositide 3-kinase γ. J. Med. Chem..

[28] Sunose M (2012). Discovery of 5-(2-amino-[1,2,4]triazolo[1,5-a]pyridin-7-yl)-N-(tert-butyl) pyridine-3-sulfonamide (CZC24758), as a potent, orally bioavailable and selective inhibitor of PI3K for the treatment of inflammatory disease. Bioorg. Med. Chem. Lett..

[29] Evans CA (2016). Discovery of a selective phosphoinositide-3-Kinase (PI3K)-γ Inhibitor (IPI-549) as an Immuno-Oncology Clinical Candidate. ACS Med. Chem. Lett..

[30] Miles DH (2020). Discovery of potent and selective 7-azaindole isoindolinone-based PI3Kγ inhibitors. ACS Med. Chem. Lett..

[31] Drew SL (2020). Discovery of potent and selective PI3Kγ inhibitors. J. Med. Chem..

[32] Bell K (2012). SAR studies around a series of triazolopyridines as potent and selective PI3Kγ inhibitors. Bioorg. Med. Chem. Lett..

[33] Zhu J (2021). Targeting phosphatidylinositol 3-kinase gamma (PI3Kγ): Discovery and development of its selective inhibitors. Med. Res. Rev..

[34] Taha MO, Al-Sha’Er MA, Khanfar MA, Al-Nadaf AH (2014). Discovery of nanomolar phosphoinositide 3-kinase gamma (PI3Kγ) inhibitors using ligand-based modeling and virtual screening followed by in vitro analysis. Eur. J. Med. Chem..

[35] Halder AK, Cordeiro MNDS (2019). Development of multi-target chemometric models for the inhibition of class I PI3K enzyme isoforms: A case study using QSAR-Co tool. Int. J. Mol. Sci..

[36] Gramatica P (2013). On the development and validation of QSAR models. Methods Mol. Biol. (Clifton, N.J.).

[37] Speck-Planche A, Cordeiro MNDS (2013). Simultaneous modeling of antimycobacterial activities and ADMET profiles: A Chemoinformatic approach to medicinal chemistry. Curr. Top. Med. Chem..

[38] Speck-Planche A, Cordeiro MNDS (2014). Chemoinformatics for medicinal chemistry: In silico model to enable the discovery of potent and safer anti-cocci agents. Future Med. Chem.

[39] Speck-Planche A, Natalia Dias Soeiro Cordeiro MNDS (2017). Speeding up early drug discovery in antiviral research: A fragment-based in silico approach for the design of virtual anti-hepatitis C leads. ACS Comb. Sci..

[40] Lipinski CA, Lombardo F, Dominy BW, Feeney PJ (2012). Experimental and computational approaches to estimate solubility and permeability in drug discovery and development settings. Adv. Drug Deliv. Rev..

[41] Ellard K (2012). Discovery of novel PI3Kγ/δ inhibitors as potential agents for inflammation. Bioorg. Med. Chem. Lett..

[42] DRAGON Version 5.5, Todeschini, R., Consonni, V., Mauri, A. & Pavan, M. TALETE SRL: Milano, Italy, (2007); software available at http://www.talete.mi.it . (Accessed 07 Mar 2021).

[43] (Data warrior Version 05.05.0) software available at http://www.openmolecules.org/datawarrior/. (Accessed 20 Jan 2021).

[44] Open Babel Version 2.3.2. (2012) software available at http://openbabel.org/. (Accessed 10 Feb 2021).

[45] HyperChem Version 8.0, Hypercube, Inc. (2007); software available at http://www.hyper.com. (Accessed 10 Oct 2020).

[46] Sadeghi F, Afkhami A, Madrakian T, Ghavami R (2021). Computational study on subfamilies of piperidine derivatives: QSAR modelling, model external verification, the inter-subset similarity determination, and structure-based drug designing. SAR QSAR Environ. Res..

[47] Sadeghi F, Afkhami A, Madrakian T, Ghavami R (2021). A new approach for simultaneous calculation of pIC_50_ and logP through QSAR/QSPR modeling on anthracycline derivatives: A comparable study. J. Iran. Chem. Soc..

[48] Hassanat A (2019). Choosing mutation and crossover ratios for genetic algorithms-a review with a new dynamic approach. Information.

[49] MATLAB Version 9.0, math work. Inc., Natick, MA, USA, (2016); software available at http://www.mathworks.com. (Accessed 15 Nov 2020).

[50] Snee RD (1977). Validation of regression models: Methods and examples. Technometrics.

[51] Kennard RW, Stone LA (1969). Computer aided design of experimental. Technometrics.

[52] Wu, W., May, R., Dandy, G.C. & Maier, H. R. A method for comparing data splitting approaches for developing hydrological ANN models. In: *The 6th International Congress on Environmental Nodelling and Software (iEMSs), Leipzig, Germany* (2012).

[53] Puzyn T, Mostrag-Szlichtyng A, Gajewicz A, Skrzyński M, Worth AP (2011). Investigating the influence of data splitting on the predictive ability of QSAR/QSPR models. Struct. Chem..

[54] May RJ, Maier HR, Dandy GC (2010). Data splitting for artificial neural networks using SOM-based stratified sampling. Neural Netw..

[55] Minitab Version 18.0 software available at https://www.minitab.com/en-us/.

[56] SPSS software Version 26.0 (2019) software available at https://www.ibm.com/analytics/spss-statistics-software.

[57] Kato Y, Hamada S, Goto H (2020). Validation study of QSAR/DNN models using the competition datasets. Mol. Inform..

[58] Gramatica P (2007). Principles of QSAR models validation: Internal and external. QSAR Comb. Sci..

[59] Tropsha A (2010). Best practices for QSAR model development, validation, and exploitation. Mol. Inform..

[60] Goncalves, I., Silva, S., Melo, J. B. M. & Carreiras, J. M. B. Random sampling technique for overfitting control in genetic programming. In *Proceedings of the 15th European Conference on Genetic Programming*. 218–229 (Springer, 2012).

[61] Cawley GC, Talbot NLC (2010). On over-fitting in model selection and subsequent selection bias in performance evaluation. J. Mach. Learn. Res..

[62] Yang C (2017). Discovery of a novel series of 7-azaindole scaffold derivatives as PI3K inhibitors with potent activity. ACS Med. Chem. Lett..

[63] https://www.medchemexpress.com/Targets/PI3K.html. (Accessed 28 Feb 2021).

[64] Pemberton N (2018). Discovery of highly isoform selective orally bioavailable phosphoinositide 3-kinase (PI3K)-γ inhibitors. J. Med. Chem..

[65] Miller MS, Thompson PE, Gabelli SB (2019). Structural determinants of isoform selectivity in pi3k inhibitors. Biomolecules.

[66] De Fortuny EJ, Martens D, Provost F (2013). Predictive modeling with big data: Is bigger really better?. Big Data.

[67] Cherkasov A (2014). QSAR modeling: Where have you been? Where are you going to?. J. Med. Chem..

[68] Jaworska J, Nikolova-Jeliazkova N, Aldenberg T (2005). QSAR applicability domain estimation by projection of the training set in descriptor space: A review. Altern. Lab. Anim..

[69] Schuur JH, Selzer P, Gasteiger J (1996). The coding of the three-dimensional structure of molecules by molecular transforms and its application to structure-spectra correlations and studies of biological activity. J. Chem. Inf. Comput. Sci..

[70] Hemmer MC, Steinhauer V, Gasteiger J (1999). Deriving the 3D structure of organic molecules from their infrared spectra. Vib. Spectrosc..

[71] Gramatica P, Corradi M, Consonni V (2000). Modelling and prediction of soil sorption coefficients of non-ionic organic pesticides by molecular descriptors. Chemosphere.

[72] Moreau G, Broto P (1980). Autocorrelation of a topological structure: A new molecular descriptor. Nouv. J. Chim..

[73] Asadollahi T, Dadfarnia S, Shabani AMH, Ghasemi JB, Sarkhosh M (2011). QSAR models for cxcr2 receptor antagonists based on the genetic algorithm for data preprocessing prior to application of the pls linear regression method and design of the new compounds using in silico virtual screening. Molecules.

[74] Sadeghi F, Afkhami A, Madrakian T, Ghavami R (2021). Computational study to select the capable anthracycline derivatives through an overview of drug structure-specificity and cancer cell line-specificity. Chem. Pap..

[75] Devinyak O, Havrylyuk D, Lesyk R (2014). 3D-MoRSE descriptors explained. J. Mol. Graph. Model..

