# Supplementary material for: QSAR analysis on a large and diverse set of potent phosphoinositide 3-kinase gamma (PI3Kγ) inhibitors using MLR and ANN methods
Source: Sci Rep. 2022 Apr 12;12:6090. doi: 10.1038/s41598-022-09843-0 (PMC9005662; doi:10.1038/s41598-022-09843-0)
Supplement: Supplementary file 1 — Supplementary Information. [file 41598_2022_9843_MOESM1_ESM.docx]

Electronic Supporting Information on the [**[Scientific Reports](https://www.sciencedirect.com/journal/journal-of-molecular-graphics-and-modelling)** [publication entitled](https://www.sciencedirect.com/journal/journal-of-molecular-graphics-and-modelling)](https://www.nature.com/srep/)

**[QSAR](https://www.sciencedirect.com/journal/journal-of-molecular-graphics-and-modelling)** **[analysis on a](https://www.sciencedirect.com/journal/journal-of-molecular-graphics-and-modelling)** **[large and diverse set of potent phosphoinositide 3-kinase gamma (PI3Kγ) inhibitors using MLR and](https://www.sciencedirect.com/journal/journal-of-molecular-graphics-and-modelling)** **[ANN methods](https://www.sciencedirect.com/journal/journal-of-molecular-graphics-and-modelling)**

[Fereydoun Sadeghi](https://pubmed.ncbi.nlm.nih.gov/?term=Sadeghi+F&cauthor_id=33960256)^1^, [Abbas Afkhami](https://pubmed.ncbi.nlm.nih.gov/?term=Afkhami+A&cauthor_id=33960256)[^1^](https://pubmed.ncbi.nlm.nih.gov/33960256/#affiliation-1)^,^[^2^](https://pubmed.ncbi.nlm.nih.gov/33960256/#affiliation-2),* [Tayyebeh Madrakian](https://pubmed.ncbi.nlm.nih.gov/?term=Madrakian+T&cauthor_id=33960256)[^1^](https://pubmed.ncbi.nlm.nih.gov/33960256/#affiliation-1)^,3^, [Raouf Ghavami](https://pubmed.ncbi.nlm.nih.gov/?term=Ghavami+R&cauthor_id=33960256)^4^

^1^Faculty of Chemistry, Bu-Ali Sina University, Hamedan, Iran.

^2^D-8 International University, Hamedan, Iran.

^3^Autophagy Research Center, Shiraz University of Medical Sciences, Shiraz, Iran

^4^Chemometrics Laboratory, Chemistry Department, Faculty of Science, University of Kurdistan, Sanandaj, Iran.

*Corresponding author, Tel. / Fax: +98 81 38272404

E. mail: [afkhami@basu.ac.ir](mailto:afkhami@basu.ac.ir) (A. Afkhami)

**Table S1**. Structures of 245 PI3Kγ inhibitors used as training and test sets and corresponding experimental pIC_50_ values

| **Compd.** | **Structures of PI3Kγ inhibitors** |  | **Exp. (pIC_50_)** | **Ref.** |
| --- | --- | --- | --- | --- |
|  |  |  | 7.05 | 31 |
| 1 | 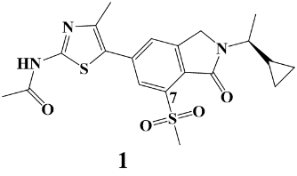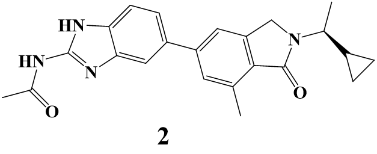 | |  |  |
| 2 |  |  | 6.32 | 31 |
| **Compd.** | **PI3Kγ inhibitor scaffold** | **R** | **Exp. (pIC_50_)** | **Ref.** |
| 3 |  | NH_2_ | 7.30 | 31 |
| 4 |  | NHCOMe | 7.10 | 31 |
| 5 |  | NH_2_ | 6.54 | 31 |
| 6 |  | NHCOMe | 7.12 | 31 |
| 7 |  | NH_2_ | 6.37 | 31 |
| 8 |  | NHCOMe | 8.26 | 31 |
| 9 |  | NH_2_ | 7.77 | 31 |
| 10 |  | H | 7.82 | 31 |
| 11 |  | NH_2_ | 7.22 | 31 |
| 12 |  | NHCOMe | 8.36 | 31 |
| 13 |  | NH_2_ | 8.54 | 31 |
| 14 |  | NHCOMe | 6.37 | 31 |
| 15 |  | H | 8.04 | 31 |
| 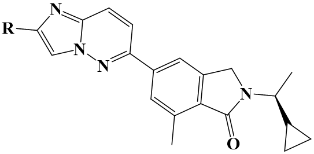 | | | |  |
| **Compd.** | **R** | | **Exp. (pIC_50_)** | **Ref.** |
| 16 | 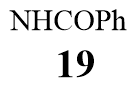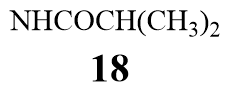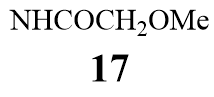 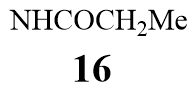    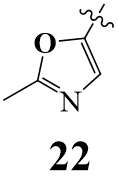  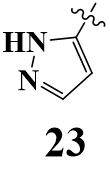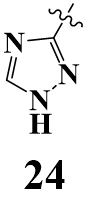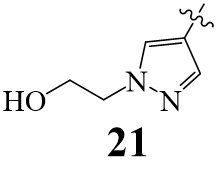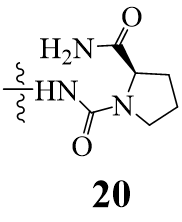  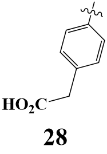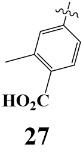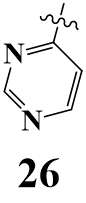 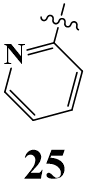 | | 8.14 | 31 |
| 17 |  |  | 7.85 | 31 |
| 18 |  |  | 7.15 | 31 |
| 19 |  |  | 7.03 | 31 |
| 20 |  |  | 8.54 | 31 |
| 21 |  |  | 8.19 | 31 |
| 22 |  |  | 8.23 | 31 |
| 23 |  |  | 7.36 | 31 |
| 24 |  |  | 7.51 | 31 |
| 25 |  |  | 7.19 | 31 |
| 26 |  |  | 8.12 | 31 |
| 27 |  |  | 8.66 | 31 |
| 28 |  |  | 8.68 | 31 |
| **Compd.** | **PI3Kγ inhibitor scaffold** | **R** | **Exp. (pIC_50_)** | **Ref.** |
| 29 |  | CF_3_ | 8.66 | 31 |
| 30 |  | NHSO_2_Me | 8.89 | 31 |
| 31 |  | SO_2_NHMe | 8.57 | 31 |
| 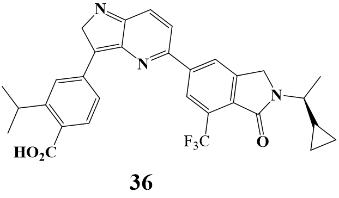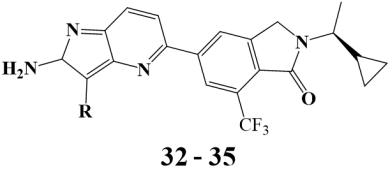 | | | |  |
| **Compd.** | **R** | | **Exp. (pIC_50_)** | **Ref.** |
| 32 | 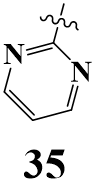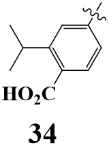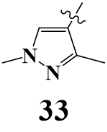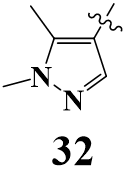 | | 8.18 | 31 |
| 33 |  |  | 8.10 | 31 |
| 34 |  |  | 7.28 | 31 |
| 35 |  |  | 9.00 | 31 |
| 36 |  |  | 8.68 | 31 |
| 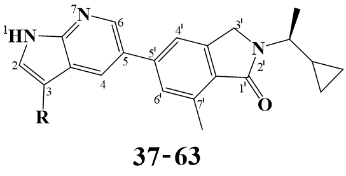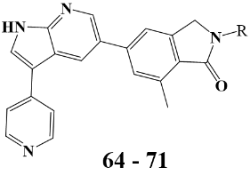 | | | |  |
| **Compd.** | **R** | | **Exp. (pIC_50_)** | **Ref.** |
| 37 | 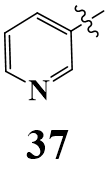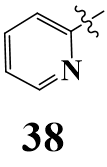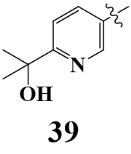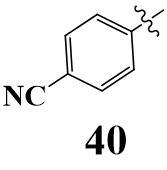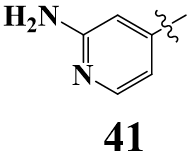      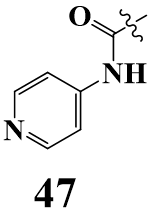  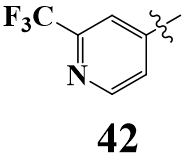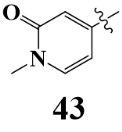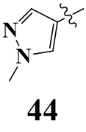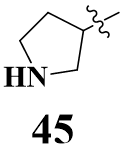  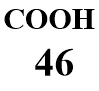      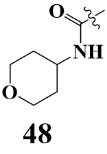  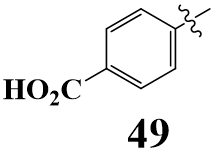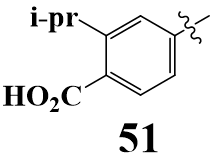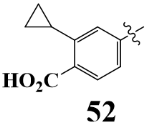  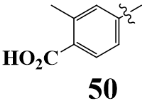      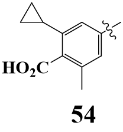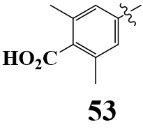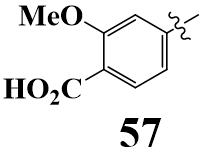  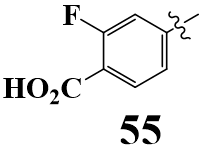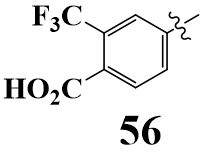      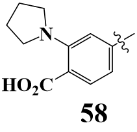  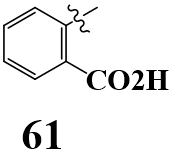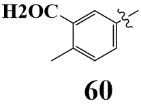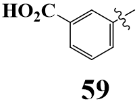    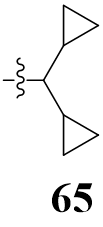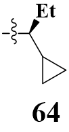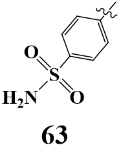  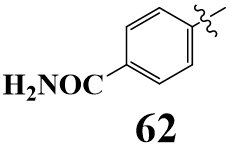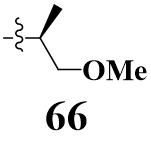    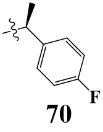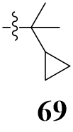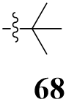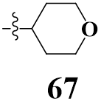  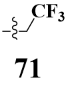 | | 8.15 | 30 |
| 38 |  |  | 8.48 | 30 |
| 39 |  |  | 8.48 | 30 |
| 40 |  |  | 7.38 | 30 |
| 41 |  |  | 8.24 | 30 |
| 42 |  |  | 7.80 | 30 |
| 43 |  |  | 8.59 | 30 |
| 44 |  |  | 8.49 | 30 |
| 45 |  |  | 6.55 | 30 |
| 46 |  |  | 7.89 | 30 |
| 47 |  |  | 7.92 | 30 |
| 48 |  |  | 8.35 | 30 |
| 49 |  |  | 8.60 | 30 |
| 50 |  |  | 8.55 | 30 |
| 51 |  |  | 8.68 | 30 |
| 52 |  |  | 8.80 | 30 |
| 53 |  |  | 8.66 | 30 |
| 54 |  |  | 8.92 | 30 |
| 55 |  |  | 8.51 | 30 |
| 56 |  |  | 8.79 | 30 |
| 57 |  |  | 8.82 | 30 |
| 58 |  |  | 8.40 | 30 |
| 59 |  |  | 8.57 | 30 |
| 60 |  |  | 8.30 | 30 |
| 61 |  |  | 6.74 | 30 |
| 62 |  |  | 8.59 | 30 |
| 63 |  |  | 8.30 | 30 |
| 64 |  |  | 8.22 | 30 |
| 65 |  |  | 8.17 | 30 |
| 66 |  |  | 8.05 | 30 |
| 67 |  |  | 7.30 | 30 |
| 68 |  |  | 7.59 | 30 |
| 69 |  |  | 7.55 | 30 |
| 70 |  |  | 7.40 | 30 |
| 71 |  |  | 7.60 | 30 |
| **Compd.** | **PI3Kγ inhibitor scaffold** | **R** | **Exp. (pIC_50_)** | **Ref.** |
| 72 | 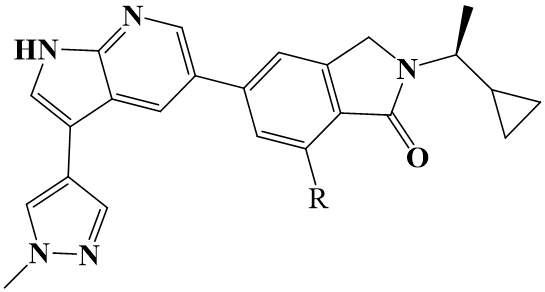 | Isopropyl | 8.38 | 30 |
| 73 |  | OMe | 8.37 | 30 |
| 74 |  | Cl | 8.62 | 30 |
| 75 |  | CN | 8.43 | 30 |
| 76 |  | CF_3_ | 8.62 | 30 |
| 77 |  | CONHMe | 8.10 | 30 |
| 78 |  | NHAc | 8.60 | 30 |
| 79 |  | SO_2_NHMe | 8.40 | 30 |
| 80 |  | SO_2_NMe_2_ | 8.59 | 30 |
| 81 |  | NHSO_2_Me | 8.40 | 30 |
| 82 |  | SO_2_Me | 8.82 | 30 |
| **Compd.** | **PI3Kγ inhibitor scaffold** | **R** | **Exp. (pIC_50_)** | **Ref.** |
| 83 | 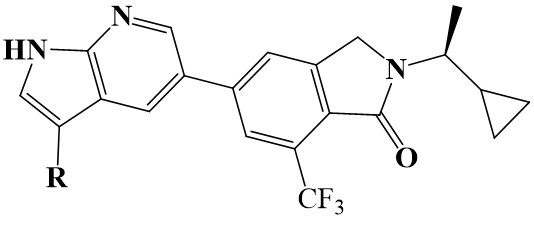 | 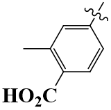 | 8.92 | 30 |
| 84 |  | 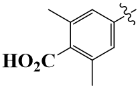 | 8.55 | 30 |
| 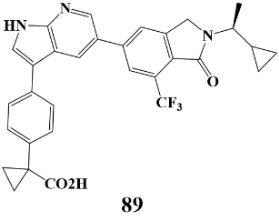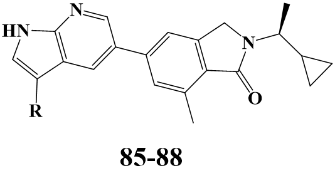 | | | |  |
| **Compd.** | **R** | | **Exp. (pIC_50_)** | **Ref.** |
| 85 | 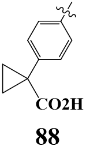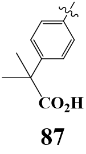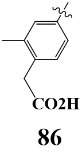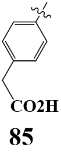 | | 8.59 | 30 |
| 86 |  |  | 8.48 | 30 |
| 87 |  |  | 8.68 | 30 |
| 88 |  |  | 8.48 | 30 |
| 89 |  |  | 8.44 | 30 |
| **Compd.** | **Structures of PI3Kγ inhibitors** | | **Exp. (pIC_50_)** | **Ref.** |
| 90 | 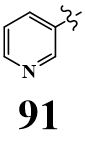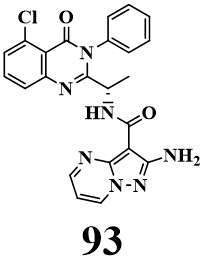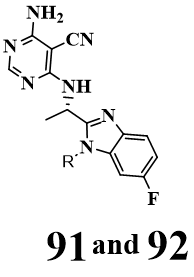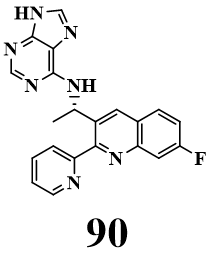  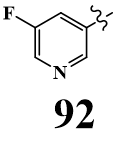      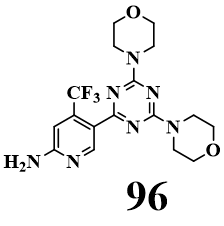  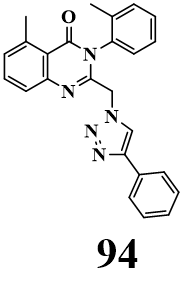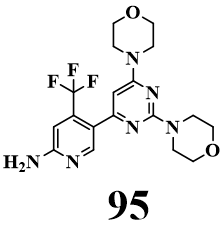      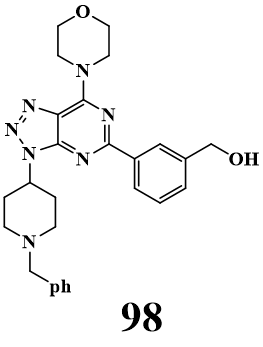  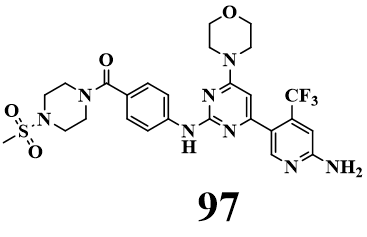  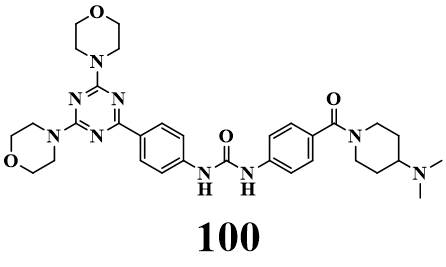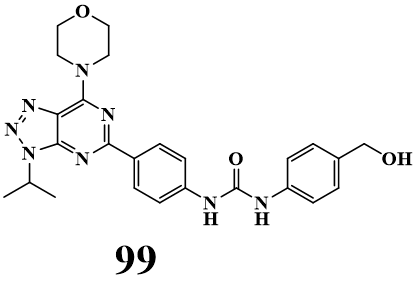  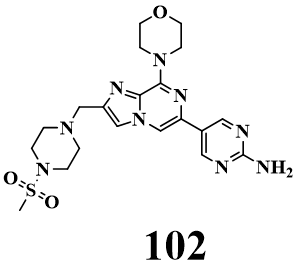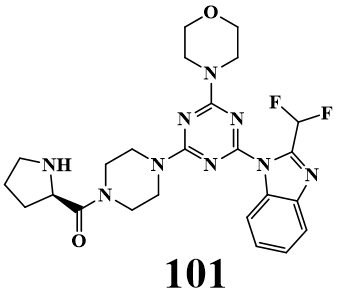  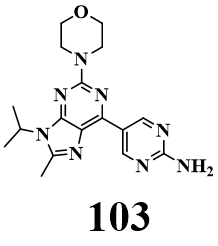    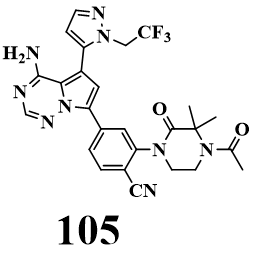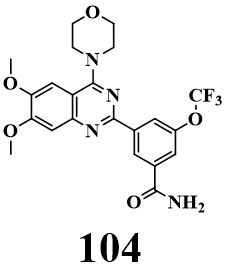  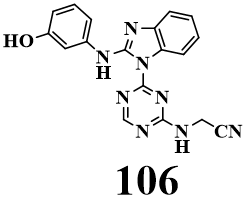  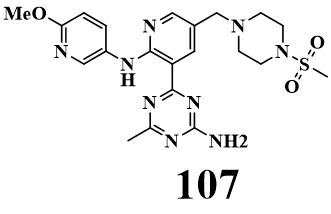  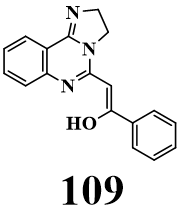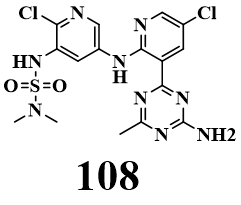  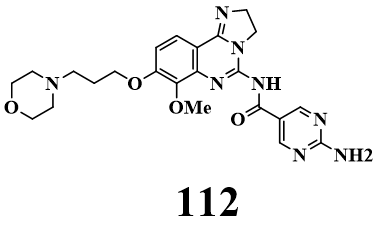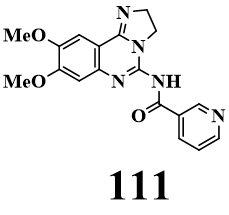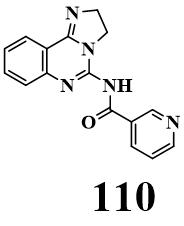 | | 6.07 | 24 |
| 91 |  |  | 5.24 | 24 |
| 92 |  |  | 5.23 | 24 |
| 93 |  |  | 7.40 | 24 |
| 94 |  |  | 6.00 | 24 |
| 95 |  |  | 6.58 | 24 |
| 96 |  |  | 7.60 | 24 |
| 97 |  |  | 7.10 | 24 |
| 98 |  |  | 6.36 | 24 |
| 99 |  |  | 7.61 | 24 |
| 100 |  |  | 8.10 | 24 |
| 101 |  |  | 5.23 | 24 |
| 102 |  |  | 6.75 | 24 |
| 103 |  |  | 7.60 | 24 |
| 104 |  |  | 6.33 | 24 |
| 105 |  |  | 6.57 | 24 |
| 106 |  |  | 7.00 | 24 |
| 107 |  |  | 8.40 | 24 |
| 108 |  |  | 9.00 | 24 |
| 109 |  |  | 6.09 | 24 |
| 110 |  |  | 7.22 | 24 |
| 111 |  |  | 7.22 | 24 |
| 112 |  |  | 8.19 | 24 |
| **Compd.** | **Structures of PI3Kγ inhibitors** | | **Exp. (pIC_50_)** | **Ref.** |
| 113 | 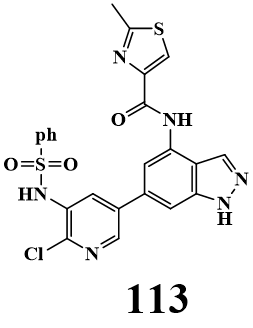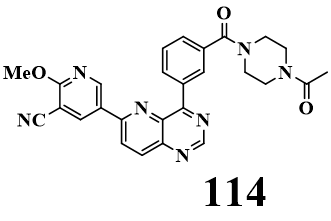  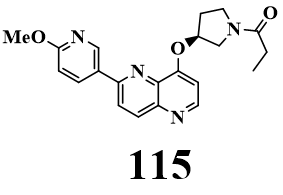  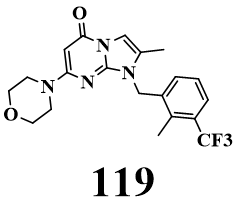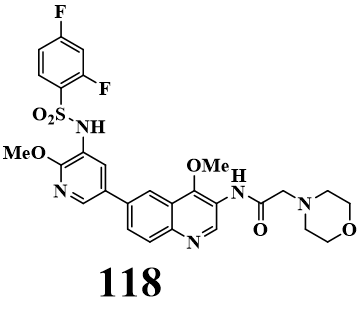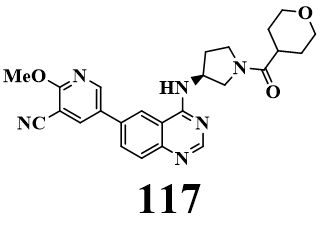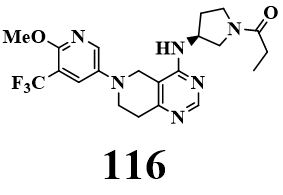 | | 7.30 | 24 |
| 114 |  |  | 5.33 | 24 |
| 115 |  |  | 5.53 | 24 |
| 116 |  |  | 5.65 | 24 |
| 117 |  |  | 6.76 | 24 |
| 118 |  |  | 8.08 | 24 |
| 119 |  |  | 6.00 | 24 |
| 120 |  |  | 6.10 | 24 |
| 121 |  |  | 6.29 | 24 |
| 122 |  |  | 5.52 | 24 |
| 123 |  |  | 6.17 | 24 |
| 124 |  |  | 5.70 | 24 |
| 125 |  |  | 7.60 | 24 |
| 126 |  |  | 7.60 | 24 |
| 127 |  |  | 8.40 | 24 |
| 128 |  |  | 8.30 | 24 |
| 129 |  |  | 8.00 | 24 |
| 130 |  |  | 8.52 | 24 |
| 131 |  |  | 7.54 | 24 |
| 132 |  |  | 6.80 | 24 |
| 133 |  |  | 8.90 | 24 |
| 134 |  |  | 8.10 | 24 |
| **Compd.** | **PI3Kγ inhibitor scaffold** | **R** | **Exp. (pIC_50_)** | **Ref.** |
| 135 |  | 2-OMe | 5.40 | 32 |
| 136 |  | 3-OMe | 6.80 | 32 |
| 137 |  | 4-diOMe | 6.00 | 32 |
| 138 |  | 3,4-diOMe | 7.40 | 32 |
| 139 |  | 3-SO_2_Me | 6.30 | 32 |
| 140 |  | 3-NHSO_2_Me | 6.50 | 32 |
| 141 |  | 3-SO_2_NHMe | 6.60 | 32 |
| 142 |  | 3-CONHMe | 6.20 | 32 |
| 143 |  | 3-SO_2_NH^n^Bu | 6.70 | 32 |
| 144 |  | 3-SO_2_NHBn | 6.30 | 32 |
|  | | | |  |
| **Compd.** | **R** | | **Exp. (pIC_50_)** | **Ref.** |
| 145 |  | | 6.60 | 32 |
| 146 |  |  | 5.40 | 32 |
| 147 |  |  | 5.50 | 32 |
| 148 |  |  | 5.80 | 32 |
| 149 |  |  | 5.40 | 32 |
| 150 |  |  | 6.40 | 32 |
| 151 |  |  | 7.20 | 32 |
| 152 |  |  | 7.10 | 32 |
| 153 |  |  | 8.10 | 32 |
| **Compd.** | **Structures of PI3Kγ inhibitors** | | **Exp. (pIC_50_)** | **Ref.** |
| 154 |  | | 7.00 | 32 |
| 155 |  |  | 6.50 | 32 |
| **Compd.** | **PI3Kγ inhibitor scaffold** | **R** | **Exp. (pIC_50_)** | **Ref.** |
| 156 |  | H | 6.50 | 32 |
| 157 |  | Me | 5.50 | 32 |
|  | | | |  |
| **Compd.** | **R** | | **Exp. (pIC_50_)** | **Ref.** |
| 158 |  | | 8.20 | 32 |
| 159 |  |  | 6.20 | 32 |
| 160 |  |  | 6.60 | 32 |
| 161 |  |  | 7.89 | 32 |
| 162 |  |  | 7.50 | 32 |
| 163 |  |  | 7.80 | 32 |
| 164 |  |  | 6.80 | 32 |
| 165 |  |  | 7.00 | 32 |
| 166 |  |  | 6.00 | 32 |
| **Compd.** | **Structures of PI3Kγ inhibitors** | | **Exp. (pIC_50_)** | **Ref.** |
| 167 |  | | 5.70 | 32 |
| 168 |  |  | 6.90 | 32 |
| 169 |  |  | 5.68 | 18 |
| 170 |  |  | 5.64 | 18 |
| 171 |  |  | 6.72 | 18 |
| 172 |  |  | 5.52 | 18 |
| 173 |  |  | 7.57 | 18 |
| 174 |  |  | 7.60 | 18 |
| 175 |  |  | 7.55 | 18 |
| 176 |  |  | 8.24 | 18 |
| 177 |  |  | 7.38 | 18 |
| 178 |  |  | 8.52 | 18 |
| 179 |  |  | 7.42 | 18 |
| 180 |  |  | 6.72 | 18 |
| 181 |  |  | 7.16 | 18 |
| 182 |  |  | 6.96 | 18 |
| 183 |  |  | 6.00 | 18 |
| 184 |  |  | 7.44 | 18 |
| 185 |  |  | 7.82 | 18 |
| 186 |  |  | 8.70 | 18 |
| 187 |  |  | 8.52 | 18 |
| 188 |  |  | 8.10 | 18 |
| 189 |  |  | 8.40 | 18 |
| 190 |  |  | 6.80 | 18 |
| 191 |  |  | 6.00 | 18 |
| 192 |  |  | 9.22 | 18 |
| 193 |  |  | 6.10 | 18 |
| 194 |  |  | 6.60 | 18 |
|  | | | |  |
| **Compd.** | **R** | | **Exp. (pIC_50_)** | **Ref.** |
| 195 |  | | 7.40 | 29 |
| 196 |  |  | 7.22 | 29 |
| 197 |  |  | 6.40 | 29 |
| 198 |  |  | 6.00 | 29 |
| 199 |  |  | 6.52 | 29 |
| 200 |  |  | 6.82 | 29 |
| **Compd.** | **PI3Kγ inhibitor scaffold** | **R** | **Exp. (pIC_50_)** | **Ref.** |
| 201 |  | Me | 6.96 | 29 |
| 202 |  | F | 7.30 | 29 |
| 203 |  | OMe | 6.00 | 29 |
| 204 |  | NHMe | 5.96 | 29 |
| 205 |  | NMe2 | 5.96 | 29 |
| 206 |  |  | 6.48 | 29 |
| 207 |  |  | 7.85 | 29 |
| 208 |  |  | 7.60 | 29 |
|  | | | |  |
| **Compd.** | **R** | | **Exp. (pIC_50_)** | **Ref.** |
| 209 |  | | 6.55 | 29 |
| 210 |  |  | 6.96 | 29 |
| 211 |  |  | 6.46 | 29 |
| 212 |  |  | 6.77 | 29 |
| 213 |  |  | 6.96 | 29 |
| 214 |  |  | 7.12 | 29 |
| 215 |  |  | 6.44 | 29 |
| 216 |  |  | 7.00 | 29 |
| 217 |  |  | 7.10 | 41 |
| 218 |  |  | 7.90 | 41 |
| 219 |  |  | 6.90 | 41 |
| 220 |  |  | 6.90 | 41 |
| 221 |  |  | 7.80 | 41 |
| 222 |  |  | 6.60 | 41 |
| 223 |  |  | 7.20 | 41 |
| 224 |  |  | 7.50 | 41 |
| 225 |  |  | 7.60 | 41 |
| 226 |  |  | 7.60 | 41 |
| 227 |  |  | 8.10 | 41 |
| 228 |  |  | 8.20 | 41 |
| 229 |  |  | 8.00 | 41 |
| 230 |  |  | 8.60 | 41 |
| 231 |  |  | 7.20 | 41 |
| 232 |  |  | 8.50 | 41 |
| 233 |  |  | 7.80 | 41 |
| 234 |  |  | 8.20 | 41 |
| 235 |  |  | 8.40 | 41 |
| 236 |  |  | 8.60 | 41 |
| **Compd.** | **PI3Kγ inhibitor scaffold** | **R** | **Exp. (pIC_50_)** | **Ref.** |
| 237 |  | NH_2_ | 7.70 | 28 |
| 238 |  | NHMe | 7.60 | 28 |
| 239 |  | NHPr | 7.50 | 28 |
| 240 |  | NHCH_2_CF_3_ | 7.70 | 28 |
| 241 |  | NHCMe_2_CF_3_ | 7.70 | 28 |
| 242 |  | NHPh | 7.40 | 28 |
| 243 |  | NEt_2_ | 8.10 | 28 |
| 244 |  |  | 7.60 | 28 |
| 245 |  | NHCMe_3_ | 7.80 | 28 |

**Table S2.** SMILES strings of the 245 PI3Kγ inhibitors (training and test sets) used to QSAR model 1 (Eq. **4**)development on pIC_50_ of these compounds

| **No.** | **SMILES strings** |
| --- | --- |
| 1 | O=C1N([C@@H](C)C2CC2)CC3=CC(C4=C(C)N=C(NC(C)=O)S4)=CC(S(=O)(C)=O)=C31 |
| 2 | O=C1N(C(C)C2CC2)CC3=C1C(C)=CC(C4=CC=C(NC(NC(C)=O)=N5)C5=C4)=C3 |
| 3 | NC1=NN2C(C=C(C=C2)C=2C=C3CN(C(C3=C(C2)C)=O)[C@@H](C)C2CC2)=N1 |
| 4 | C1(CC1)[C@H](C)N1C(C2=C(C=C(C=C2C1)C1=CC=2N(C=C1)N=C(N2)NC(C)=O)C)=O |
| 5 | NC1=NN(C=C(C2=CC(CN(C(C)C3CC3)C4=O)=C4C(C)=C2)C=C5)C5=N1 |
| 6 | O=C1N(C(C)C2CC2)CC3=C1C(C)=CC(C(C=C4)=CN5C4=NC(NC(C)=O)=N5)=C3 |
| 7 | NC1=CN(N=C(C2=CC(CN(C(C)C3CC3)C4=O)=C4C(C)=C2)C=C5)C5=N1 |
| 8 | O=C1N([C@@H](C)C2CC2)CC3=C1C(C)=CC(C(C=C4)=NN5C4=NC(NC(C)=O)=C5)=C3 |
| 9 | NC1=C(C2=CN(C)N=C2)N(N=C(C3=CC(CN([C@@H](C)C4CC4)C5=O)=C5C(C)=C3)C=C6)C6=N1 |
| 10 | O=C1N([C@@H](C)C2CC2)CC3=C1C(C)=CC(C(C=C4)=NN5C4=NC=C5C6=CN(C)N=C6)=C3 |
| 11 | O=C1N([C@@H](C)C2CC2)CC3=C1C(C)=CC(C4=NC5=CC(N)=NN5C=C4)=C3 |
| 12 | O=C1N([C@@H](C)C2CC2)CC3=C1C(C)=CC(C4=NC5=CC(NC(C)=O)=NN5C=C4)=C3 |
| 13 | O=C1N([C@@H](C)C2CC2)CC3=C1C(C)=CC(C4=NC5=C(C6=CN(C)N=C6)C(N)=NN5C=C4)=C3 |
| 14 | O=C1N([C@@H](C)C2CC2)CC3=C1C(C)=CC(C4=NC5=C(C6=CN(C)N=C6)C(NC(C)=O)=NN5C=C4)=C3 |
| 15 | O=C1N([C@@H](C)C2CC2)CC3=C1C(C)=CC(C4=NC5=C(C6=CN(C)N=C6)C=NN5C=C4)=C3 |
| 16 | O=C1N([C@@H](C)C2CC2)CC3=C1C(C)=CC(C(C=C4)=NN5C4=NC(NC(CC)=O)=C5)=C3 |
| 17 | O=C1N([C@@H](C)C2CC2)CC3=C1C(C)=CC(C(C=C4)=NN5C4=NC(NC(COC)=O)=C5)=C3 |
| 18 | O=C1N([C@@H](C)C2CC2)CC3=C1C(C)=CC(C(C=C4)=NN5C4=NC(NC(C(C)C)=O)=C5)=C3 |
| 19 | O=C1N([C@@H](C)C2CC2)CC3=C1C(C)=CC(C(C=C4)=NN5C4=NC(NC(C6=CC=CC=C6)=O)=C5)=C3 |
| 20 | O=C1N([C@@H](C)C2CC2)CC3=C1C(C)=CC(C(C=C4)=NN5C4=NC(NC(N6[C@H](C(N)=O)CCC6)=O)=C5)=C3 |
| 21 | O=C1N([C@@H](C)C2CC2)CC3=C1C(C)=CC(C4=NC5=C(C6=CN(CCO)N=C6)C(N)=NN5C=C4)=C3 |
| 22 | O=C1N([C@@H](C)C2CC2)CC3=C1C(C)=CC(C4=NC5=C(C6=CN=C(C)O6)C(N)=NN5C=C4)=C3 |
| 23 | O=C1N([C@@H](C)C2CC2)CC3=C1C(C)=CC(C4=NC5=C(C6=CC=NN6)C(N)=NN5C=C4)=C3 |
| 24 | O=C1N([C@@H](C)C2CC2)CC3=C1C(C)=CC(C4=NC5=C(C6=NNC=N6)C(N)=NN5C=C4)=C3 |
| 25 | O=C1N([C@@H](C)C2CC2)CC3=C1C(C)=CC(C4=NC5=C(C6=NC=CC=C6)C(N)=NN5C=C4)=C3 |
| 26 | O=C1N([C@@H](C)C2CC2)CC3=C1C(C)=CC(C4=NC5=C(C6=NC=NC=C6)C(N)=NN5C=C4)=C3 |
| 27 | O=C1N([C@@H](C)C2CC2)CC3=C1C(C)=CC(C4=NC5=C(C6=CC(C)=C(C(O)=O)C=C6)C(N)=NN5C=C4)=C3 |
| 28 | O=C1N([C@@H](C)C2CC2)CC3=C1C(C)=CC(C4=NC5=C(C6=CC=C(CC(O)=O)C=C6)C(N)=NN5C=C4)=C3 |
| 29 | O=C1N([C@@H](C)C2CC2)CC3=C1C(C(F)(F)F)=CC(C4=NC5=CC(NC(C)=O)=NN5C=C4)=C3 |
| 30 | O=C1N([C@@H](C)C2CC2)CC3=C1C(NS(=O)(C)=O)=CC(C4=NC5=CC(NC(C)=O)=NN5C=C4)=C3 |
| 31 | O=C1N([C@@H](C)C2CC2)CC3=C1C(S(=O)(NC)=O)=CC(C4=NC5=CC(NC(C)=O)=NN5C=C4)=C3 |
| 32 | O=C1N([C@@H](C)C2CC2)CC3=C1C(C(F)(F)F)=CC(C4=NC5=C(C6=C(C)N(C)N=C6)C(N)=NN5C=C4)=C3 |
| 33 | O=C1N([C@@H](C)C2CC2)CC3=C1C(C(F)(F)F)=CC(C4=NC5=C(C6=CN(C)N=C6C)C(N)=NN5C=C4)=C3 |
| 34 | O=C1N([C@@H](C)C2CC2)CC3=C1C(C(F)(F)F)=CC(C4=NC5=C(C6=NC=CC=N6)C(N)=NN5C=C4)=C3 |
| 35 | O=C1N([C@@H](C)C2CC2)CC3=C1C(C(F)(F)F)=CC(C4=NC5=C(C6=CC(C(C)C)=C(C(O)=O)C=C6)C(N)=NN5C=C4)=C3 |
| 36 | O=C1N([C@@H](C)C2CC2)CC3=C1C(C(F)(F)F)=CC(C4=NC5=C(C6=CC(C(C)C)=C(C(O)=O)C=C6)C=NN5C=C4)=C3 |
| 37 | C1(CC1)C(C)N1C(C2=C(C=C(C=C2C1)C=1C=C2C(=NC1)NC=C2C=2C=NC=CC2)C)=O |
| 38 | C1(CC1)[C@H](C)N1C(C2=C(C=C(C=C2C1)C=1C=C2C(=NC1)NC=C2C2=NC=CC=C2)C)=O |
| 39 | C1(CC1)[C@H](C)N1C(C2=C(C=C(C=C2C1)C=1C=C2C(=NC1)NC=C2C=2C=NC(=CC2)C(C)(C)O)C)=O |
| 40 | C1(CC1)[C@H](C)N1C(C2=C(C=C(C=C2C1)C=1C=C2C(=NC1)NC=C2C2=CC=C(C#N)C=C2)C)=O |
| 41 | NC1=NC=CC(=C1)C1=CNC2=NC=C(C=C21)C=2C=C1CN(C(C1=C(C2)C)=O)[C@@H](C)C2CC2 |
| 42 | C1(CC1)[C@H](C)N1C(C2=C(C=C(C=C2C1)C=1C=C2C(=NC1)NC=C2C2=CC(=NC=C2)C(F)(F)F)C)=O |
| 43 | C1(CC1)[C@H](C)N1C(C2=C(C=C(C=C2C1)C=1C=C2C(=NC1)NC=C2C2=CC(N(C=C2)C)=O)C)=O |
| 44 | C1(CC1)[C@H](C)N1C(C2=C(C=C(C=C2C1)C=1C=C2C(=NC1)NC=C2C=2C=NN(C2)C)C)=O |
| 45 | C1(CC1)[C@H](C)N1C(C2=C(C=C(C=C2C1)C=1C=C2C(=NC1)NC=C2C2CNCC2)C)=O |
| 46 | C1(CC1)[C@H](C)N1C(C2=C(C=C(C=C2C1)C=1C=C2C(=NC1)NC=C2C(=O)O)C)=O |
| 47 | N1=CC=C(C=C1)NC(=O)C1=CNC2=NC=C(C=C21)C=2C=C1CN(C(C1=C(C2)C)=O)C(C)C2CC2 |
| 48 | O1CCC(CC1)NC(=O)C1=CNC2=NC=C(C=C21)C=2C=C1CN(C(C1=C(C2)C)=O)C(C)C2CC2 |
| 49 | C1(CC1)[C@H](C)N1C(C2=C(C=C(C=C2C1)C=1C=C2C(=NC1)NC=C2C2=CC=C(C(=O)O)C=C2)C)=O |
| 50 | C1(CC1)[C@H](C)N1C(C2=C(C=C(C=C2C1)C=1C=C2C(=NC1)NC=C2C2=CC(=C(C(=O)O)C=C2)C)C)=O |
| 51 | C1(CC1)[C@H](C)N1C(C2=C(C=C(C=C2C1)C=1C=C2C(=NC1)NC=C2C2=CC(=C(C(=O)O)C=C2)C(C)C)C)=O |
| 52 | C1(CC1)C1=C(C(=O)O)C=CC(=C1)C1=CNC2=NC=C(C=C21)C=2C=C1CN(C(C1=C(C2)C)=O)[C@@H](C)C2CC2 |
| 53 | C1(CC1)[C@H](C)N1C(C2=C(C=C(C=C2C1)C=1C=C2C(=NC1)NC=C2C2=CC(=C(C(=O)O)C(=C2)C)C)C)=O |
| 54 | C1(CC1)C1=C(C(=O)O)C(=CC(=C1)C1=CNC2=NC=C(C=C21)C=2C=C1CN(C(C1=C(C2)C)=O)[C@@H](C)C2CC2)C |
| 55 | C1(CC1)[C@H](C)N1C(C2=C(C=C(C=C2C1)C=1C=C2C(=NC1)NC=C2C2=CC(=C(C(=O)O)C=C2)F)C)=O |
| 56 | C1(CC1)[C@H](C)N1C(C2=C(C=C(C=C2C1)C=1C=C2C(=NC1)NC=C2C2=CC(=C(C(=O)O)C=C2)C(F)(F)F)C)=O |
| 57 | C1(CC1)[C@H](C)N1C(C2=C(C=C(C=C2C1)C=1C=C2C(=NC1)NC=C2C2=CC(=C(C(=O)O)C=C2)OC)C)=O |
| 58 | C1(CC1)[C@H](C)N1C(C2=C(C=C(C=C2C1)C=1C=C2C(=NC1)NC=C2C2=CC(=C(C(=O)O)C=C2)N2CCCC2)C)=O |
| 59 | C1(CC1)[C@H](C)N1C(C2=C(C=C(C=C2C1)C=1C=C2C(=NC1)NC=C2C=2C=C(C(=O)O)C=CC2)C)=O |
| 60 | C1(CC1)[C@H](C)N1C(C2=C(C=C(C=C2C1)C=1C=C2C(=NC1)NC=C2C=2C=CC(=C(C(=O)O)C2)C)C)=O |
| 61 | C1(CC1)[C@H](C)N1C(C2=C(C=C(C=C2C1)C=1C=C2C(=NC1)NC=C2C2=C(C(=O)O)C=CC=C2)C)=O |
| 62 | C1(CC1)[C@H](C)N1C(C2=C(C=C(C=C2C1)C=1C=C2C(=NC1)NC=C2C2=CC=C(C(=O)N)C=C2)C)=O |
| 63 | C1(CC1)[C@H](C)N1C(C2=C(C=C(C=C2C1)C=1C=C2C(=NC1)NC=C2C2=CC=C(C=C2)S(=O)(=O)N)C)=O |
| 64 | C1(CC1)[C@H](CC)N1C(C2=C(C=C(C=C2C1)C=1C=C2C(=NC1)NC=C2C2=CC=NC=C2)C)=O |
| 65 | C1(CC1)C(N1C(C2=C(C=C(C=C2C1)C=1C=C2C(=NC1)NC=C2C2=CC=NC=C2)C)=O)C2CC2 |
| 66 | COC[C@H](C)N1C(C2=C(C=C(C=C2C1)C=1C=C2C(=NC1)NC=C2C2=CC=NC=C2)C)=O |
| 67 | CC=1C=C(C=C2CN(C(C12)=O)C1CCOCC1)C=1C=C2C(=NC1)NC=C2C2=CC=NC=C2 |
| 68 | C(C)(C)(C)N1C(C2=C(C=C(C=C2C1)C=1C=C2C(=NC1)NC=C2C2=CC=NC=C2)C)=O |
| 69 | C1(CC1)C(C)(C)N1C(C2=C(C=C(C=C2C1)C=1C=C2C(=NC1)NC=C2C2=CC=NC=C2)C)=O |
| 70 | FC1=CC=C(C=C1)[C@H](C)N1C(C2=C(C=C(C=C2C1)C=1C=C2C(=NC1)NC=C2C2=CC=NC=C2)C)=O |
| 71 | CC=1C=C(C=C2CN(C(C12)=O)CC(F)(F)F)C=1C=C2C(=NC1)NC=C2C2=CC=NC=C2 |
| 72 | C1(CC1)[C@H](C)N1C(C2=C(C=C(C=C2C1)C=1C=C2C(=NC1)NC=C2C=2C=NN(C2)C)C(C)C)=O |
| 73 | C1(CC1)[C@H](C)N1C(C2=C(C=C(C=C2C1)C=1C=C2C(=NC1)NC=C2C=2C=NN(C2)C)OC)=O |
| 74 | ClC=1C=C(C=C2CN(C(C12)=O)[C@@H](C)C1CC1)C=1C=C2C(=NC1)NC=C2C=2C=NN(C2)C |
| 75 | C1(CC1)[C@H](C)N1CC=2C=C(C=C(C2C1=O)C#N)C=1C=C2C(=NC1)NC=C2C=2C=NN(C2)C |
| 76 | C1(CC1)[C@H](C)N1C(C2=C(C=C(C=C2C1)C=1C=C2C(=NC1)NC=C2C=2C=NN(C2)C)C(F)(F)F)=O |
| 77 | C1(CC1)[C@H](C)N1CC=2C=C(C=C(C2C1=O)C(=O)NC)C=1C=C2C(=NC1)NC=C2C=2C=NN(C2)C |
| 78 | C1(CC1)[C@H](C)N1CC2=CC(=CC(=C2C1=O)NC(C)=O)C=1C=C2C(=NC1)NC=C2C=2C=NN(C2)C |
| 79 | C1(CC1)[C@H](C)N1CC=2C=C(C=C(C2C1=O)S(=O)(=O)NC)C=1C=C2C(=NC1)NC=C2C=2C=NN(C2)C |
| 80 | C1(CC1)[C@H](C)N1CC=2C=C(C=C(C2C1=O)S(=O)(=O)N(C)C)C=1C=C2C(=NC1)NC=C2C=2C=NN(C2)C |
| 81 | C1(CC1)[C@H](C)N1CC2=CC(=CC(=C2C1=O)NS(=O)(=O)C)C=1C=C2C(=NC1)NC=C2C=2C=NN(C2)C |
| 82 | C1(CC1)[C@H](C)N1C(C2=C(C=C(C=C2C1)C=1C=C2C(=NC1)NC=C2C=2C=NN(C2)C)S(=O)(=O)C)=O |
| 83 | C1(CC1)[C@H](C)N1C(C2=C(C=C(C=C2C1)C=1C=C2C(=NC1)NC=C2C2=CC(=C(C(=O)O)C=C2)C)C(F)(F)F)=O |
| 84 | C1(CC1)[C@H](C)N1C(C2=C(C=C(C=C2C1)C=1C=C2C(=NC1)NC=C2C2=CC(=C(C(=O)O)C(=C2)C)C)C(F)(F)F)=O |
| 85 | C1(CC1)[C@H](C)N1C(C2=C(C=C(C=C2C1)C=1C=C2C(=NC1)NC=C2C2=CC=C(C=C2)CC(=O)O)C)=O |
| 86 | C1(CC1)[C@H](C)N1C(C2=C(C=C(C=C2C1)C=1C=C2C(=NC1)NC=C2C2=CC(=C(C=C2)CC(=O)O)C)C)=O |
| 87 | C1(CC1)[C@H](C)N1C(C2=C(C=C(C=C2C1)C=1C=C2C(=NC1)NC=C2C2=CC=C(C=C2)C(C(=O)O)(C)C)C)=O |
| 88 | C1(CC1)[C@H](C)N1C(C2=C(C=C(C=C2C1)C=1C=C2C(=NC1)NC=C2C2=CC=C(C=C2)C2(CC2)C(=O)O)C)=O |
| 89 | C1(CC1)[C@H](C)N1C(C2=C(C=C(C=C2C1)C=1C=C2C(=NC1)NC=C2C2=CC=C(C=C2)C2(CC2)C(=O)O)C(F)(F)F)=O |
| 90 | CC(C1=C(N=C2C=C(C=CC2=C1)F)C3=CC=CC=N3)NC4=NC=NC5=C4NC=N5 |
| 91 | N#CC1=C(N[C@H](C2=NC3=CC=C(F)C=C3N2C4=CC=CN=C4)C)N=CN=C1N |
| 92 | N#CC1=C(N[C@H](C2=NC3=CC=C(F)C=C3N2C4=CC(F)=CN=C4)C)N=CN=C1N |
| 93 | C[C@H](NC(=O)C1=C2N=CC=CN2N=C1N)C1=NC2=C(C(Cl)=CC=C2)C(=O)N1C1=CC=CC=C1 |
| 94 | CC1=CC=CC=C1N1C(CN2C=C(N=N2)C2=CC=CC=C2)=NC2=C(C(C)=CC=C2)C1=O |
| 95 | NC1=NC=C(C2=NC(=NC(=C2)N2CCOCC2)N2CCOCC2)C(=C1)C(F)(F)F |
| 96 | NC1=NC=C(C2=NC(=NC(=N2)N2CCOCC2)N2CCOCC2)C(=C1)C(F)(F)F |
| 97 | CS(=O)(=O)N1CCN(CC1)C(=O)C1=CC=C(NC2=NC(=CC(=N2)N2CCOCC2)C2=CN=C(N)C=C2C(F)(F)F)C=C1 |
| 98 | OCC1=CC(=CC=C1)C1=NC(N2CCOCC2)=C2N=NN(C3CCN(CC4=CC=CC=C4)CC3)C2=N1 |
| 99 | CC(C)N1N=NC2=C(N=C(N=C12)C1=CC=C(NC(=O)NC2=CC=C(CO)C=C2)C=C1)N1CCOCC1 |
| 100 | CN(C)C1CCN(CC1)C(=O)C1=CC=C(NC(=O)NC2=CC=C(C=C2)C2=NC(=NC(=N2)N2CCOCC2)N2CCOCC2)C=C1 |
| 101 | FC(F)C1=NC2=C(C=CC=C2)N1C1=NC(=NC(=N1)N1CCN(CC1)C(=O)C1CCCN1)N1CCOCC1 |
| 102 | CS(=O)(=O)N1CCN(CC2=CN3C=C(N=C(N4CCOCC4)C3=N2)C2=CN=C(N)N=C2)CC1 |
| 103 | C(C)(C)N1C2=NC(=NC(=C2N=C1C)C=1C=NC(=NC1)N)N1CCOCC1 |
| 104 | COC=1C=C2C(=NC(=NC2=CC1OC)C=1C=C(C(=O)N)C=C(C1)OC(F)(F)F)N1CCOCC1 |
| 105 | C(C)(=O)N1C(C(N(CC1)C1=C(C#N)C=CC(=C1)C1=CC(=C2C(=NC=NN21)N)C2=CC=NN2CC(F)(F)F)=O)(C)C |
| 106 | OC1=CC(NC2=NC3=C(C=CC=C3)N2C2=NC=NC(NCC#N)=N2)=CC=C1 |
| 107 | COC1=CC=C(NC2=C(C=C(CN3CCN(CC3)S(C)(=O)=O)C=N2)C2=NC(C)=NC(N)=N2)C=N1 |
| 108 | CN(C)S(=O)(=O)NC1=CC(NC2=C(C=C(Cl)C=N2)C2=NC(C)=NC(N)=N2)=CN=C1Cl |
| 109 | O\C(=C/C1=NC2=C(C=CC=C2)C2=NCCN12)C1=CC=CC=C1 |
| 110 | O=C(NC1=NC2=C(C=CC=C2)C2=NCCN12)C1=CC=CN=C1 |
| 111 | COC1=CC2=C(C=C1OC)C1=NCCN1C(NC(=O)C1=CC=CN=C1)=N2 |
| 112 | COC1=C(OCCCN2CCOCC2)C=CC2=C1N=C(NC(=O)C1=CN=C(N)N=C1)N1CCN=C21 |
| 113 | CC1=NC(=CS1)C(=O)NC1=CC(=CC2=C1C=NN2)C1=CC(NS(=O)(=O)C2=CC=CC=C2)=C(Cl)N=C1 |
| 114 | COC1=NC=C(C=C1C#N)C1=NC2=C(N=CN=C2C=C1)C1=CC(=CC=C1)C(=O)N1CCN(CC1)C(C)=O |
| 115 | CCC(=O)N1CC[C@@H](C1)OC1=CC=NC2=CC=C(N=C12)C1=CN=C(OC)C=C1 |
| 116 | CCC(=O)N1CC[C@@H](C1)NC1=NC=NC2=C1CN(CC2)C1=CN=C(OC)C(=C1)C(F)(F)F |
| 117 | COC1=NC=C(C=C1C#N)C1=CC=C2N=CN=C(N[C@H]3CCN(C3)C(=O)C3CCOCC3)C2=C1 |
| 118 | COC1=C(NS(=O)(=O)C2=C(F)C=C(F)C=C2)C=C(C=N1)C1=CC2=C(C=C1)N=CC(NC(=O)CN1CCOCC1)=C2OC |
| 119 | CC1=CN2C(=NC(=CC2=O)N2CCOCC2)N1CC1=C(C)C(=CC=C1)C(F)(F)F |
| 120 | CC1=NC(=O)C2=C(N=C(S2)N2CCOCC2)N1CC1=C(C)C(=CC=C1)C(F)(F)F |
| 121 | C[C@@H](NC1=CC(F)=CC(F)=C1)C1=CC(=CN2C(=O)C=C(N=C12)N1CCOCC1)C(=O)N(C)C |
| 122 | C[C@@H](NC1=CC(F)=C(F)C=C1)C1=CC(=CN2C(=O)C=C(N=C12)N1CCOCC1)C(=O)N(C)C |
| 123 | CC(C1=CC(=CC2=C1OC(=CC2=O)N3CCOCC3)C(=O)N(C)C)NC4=CC(=CC(=C4)F)F |
| 124 | CC(=O)NC1=NN2C=C(C=CC2=N1)C1=CC(C)=C(O)C(C)=C1 |
| 125 | CC(C)(C)NS(=O)(=O)C1=CC(=CN=C1)C1=CN2N=C(N)N=C2C(F)=C1 |
| 126 | CC(C)(C)NS(=O)(=O)C1=CC(=CN=C1)C1=CN2N=C(N)N=C2C=C1 |
| 127 | CC(C)(C)NS(=O)(=O)C1=CC(=CN=C1)C1=CN2N=C(NCC(=O)N3CCOCC3)N=C2C=C1 |
| 128 | CC(=O)NC1=NC(C)=C(S1)C1=CSC(NC2=CC(=CC=C2)C(O)=O)=N1 |
| 129 | CC(=O)NC1=NC(C)=C(S1)C1=COC(CC(F)(F)F)=N1 |
| 130 | CC(=O)NC1=NC(C)=C(S1)C1=COC(=N1)C(C)(C)C |
| 131 | CC(=O)NC1=NC(C)=C(S1)C1=NC(=NO1)N1CCCC(O)C1 |
| 132 | C[C@@H](C1CC1)N1CC2=C(C1=O)C(Cl)=CC(=C2)C1=CC(NC(C)=O)=NO1 |
| 133 | C[C@H](C1CC1)N1CC2=C(C1=O)C(Cl)=CC(=C2)C1=CN=C(NC(C)=O)S1 |
| 134 | C[C@@H](C1CC1)N1CC2=C(C1=O)C(=CC(=C2)C1=CN=C(NC(C)=O)S1)S(C)(=O)=O |
| 135 | COC1=C(C=CC=C1)C=1C=CC=2N(C1)N=C(N2)NC(C)=O |
| 136 | COC=1C=C(C=CC1)C=1C=CC=2N(C1)N=C(N2)NC(C)=O |
| 137 | COC1=CC=C(C=C1)C=1C=CC=2N(C1)N=C(N2)NC(C)=O |
| 138 | COC=1C=C(C=CC1OC)C=1C=CC=2N(C1)N=C(N2)NC(C)=O |
| 139 | CS(=O)(=O)C=1C=C(C=CC1)C=1C=CC=2N(C1)N=C(N2)NC(C)=O |
| 140 | CS(=O)(=O)NC=1C=C(C=CC1)C=1C=CC=2N(C1)N=C(N2)NC(C)=O |
| 141 | CNS(=O)(=O)C=1C=C(C=CC1)C=1C=CC=2N(C1)N=C(N2)NC(C)=O |
| 142 | C(C)(=O)NC1=NN2C(C=CC(=C2)C=2C=C(C(=O)NC)C=CC2)=N1 |
| 143 | C(CCC)NS(=O)(=O)C=1C=C(C=CC1)C=1C=CC=2N(C1)N=C(N2)NC(C)=O |
| 144 | C(C1=CC=CC=C1)NS(=O)(=O)C=1C=C(C=CC1)C=1C=CC=2N(C1)N=C(N2)NC(C)=O |
| 145 | N1=CC(=CC=C1)C=1C=CC=2N(C1)N=C(N2)NC(C)=O |
| 146 | N1=CC=CC(=C1)C=1C=CC=2N(C1)N=C(N2)NC(C)=O |
| 147 | C1=NC=C(C2=CC=CC=C12)C=1C=CC=2N(C1)N=C(N2)NC(C)=O |
| 148 | N1=CC=C(C=C1)C=1C=CC=2N(C1)N=C(N2)NC(C)=O |
| 149 | CN1N=CC(=C1)C=1C=CC=2N(C1)N=C(N2)NC(C)=O |
| 150 | S1C=C(C=C1)C=1C=CC=2N(C1)N=C(N2)NC(C)=O |
| 151 | COC=1C=C(C=NC1)C=1C=CC=2N(C1)N=C(N2)NC(C)=O |
| 152 | CS(=O)(=O)C=1C=C(C=NC1)C=1C=CC=2N(C1)N=C(N2)NC(C)=O |
| 153 | CS(=O)(=O)NC=1C=C(C=NC1)C=1C=CC=2N(C1)N=C(N2)NC(C)=O |
| 154 | CS(=O)(=O)C=1C=C(C=NC1)C=1C=CC=2N(C1)N=C(N2)N |
| 155 | CS(=O)(=O)NC=1C=C(C=CC1)C=1C=CC=2N(C1)N=C(N2)NC(=O)C2CCCCC2 |
| 156 | CS(=O)(=O)C=1C=C(C=NC1)C=1C=CC=2N(C1)N=C(N2)NC |
| 157 | CS(=O)(=O)C=1C=C(C=NC1)C=1C=CC=2N(C1)N=C(N2)N(C)C |
| 158 | NC1=NN2C(C=CC(=C2)C=2C=C(C=NC2)NS(=O)(=O)C2=CC=C(C=C2)Cl)=N1 |
| 159 | ClC1=CC=C(C=C1)NS(=O)(=O)C=1C=NC=C(C1)C=1C=CC=2N(C1)N=C(N2)N |
| 160 | C(C1=CC=CC=C1)NS(=O)(=O)C=1C=NC=C(C1)C=1C=CC=2N(C1)N=C(N2)N |
| 161 | C(C)(C)(C)NS(=O)(=O)C=1C=NC=C(C1)C=1C=CC=2N(C1)N=C(N2)N |
| 162 | C(C)(C)NS(=O)(=O)C=1C=NC=C(C1)C=1C=CC=2N(C1)N=C(N2)N |
| 163 | C1(CC1)CNS(=O)(=O)C=1C=NC=C(C1)C=1C=CC=2N(C1)N=C(N2)N |
| 164 | CC(C)(C)S(=O)(=O)C=1C=C(C=NC1)C=1C=CC=2N(C1)N=C(N2)N |
| 165 | CS(=O)(=O)C=1C=C(C=NC1)C=1C=C(C=2N(C1)N=C(N2)NC(C)=O)C |
| 166 | CS(=O)(=O)C=1C=C(C=NC1)C=1C=CC=2N(C1C)N=C(N2)NC(C)=O |
| 167 | ClC=1C=2N(C=C(C1)C=1C=NC=C(C1)S(=O)(=O)C)N=C(N2)N |
| 168 | FC=1C=2N(C=C(C1)C=1C=NC=C(C1)S(=O)(=O)C)N=C(N2)NC(C)=O |
| 169 | CC[C@H](NC1=C2N=CNC2=NC=N1)C1=NC2=C(C(F)=CC=C2)C(=O)N1C1=CC=CC=C1 |
| 170 | CC1=CSC2=NC(=C(C(=O)N12)C3=CC(=CC=C3)F)C(C)NC4=NC=NC5=C4NC=N5 |
| 171 | NC1=NC(N)=C(C#N)C(N[C@@H](C2CC2)C2=NC3=C(C(Cl)=CC=C3F)C(=O)N2C2=CN=CC=C2)=N1 |
| 172 | C[C@H](NC1=C2N=CNC2=NC=N1)C1=NC2=C(C=C(F)C=C2)C(=O)N1C1=CC=CC=C1 |
| 173 | C[C@H](NC1=C2N=CNC2=NC=N1)C1=CC2=C(C(Cl)=CC=C2)C(=O)N1C1=CC=CC=C1 |
| 174 | COCCN(CCOC)C(=O)CCCC#CC1=CC=CC2=C1C(=O)N(CC1=C(Cl)C=CC=C1)C(CN1NC(C3=C1N=CN=C3N)C1=CC(O)=CC=C1)=N2 |
| 175 | COCCOCCOCC#CC1=CC=CC2=C1C(=O)N(CC1=C(C=CC=C1)C(F)(F)F)C(CN1NC(C3=C1N=CN=C3N)C1=CC(O)=CC(F)=C1)=N2 |
| 176 | CC(NC1=NC(N)=NC(N)=C1C#N)C1=C(NC2=C(C=CC=C2Cl)C1=O)C1=CC=CC=C1 |
| 177 | O=C1N(C2=CC=CC=C2)C(=NN2C=CC=C12)[C@@H]1CCCN1C1=NC=NC2=C1C(=CN2)C#N |
| 178 | C[C@H]1CN([C@@H]1C1=NN2C=CC(Cl)=C2C(=O)N1C1=CC=CC=C1)C1=C2C(CCS2(=O)=O)=NC(N)=N1 |
| 179 | C[C@H](NC1=C2C(NC=C2C#N)=NC=N1)C1=CC2=NC=C(Cl)N2N=C1C1=CC=CC=C1 |
| 180 | CC1=CC(Cl)=C(C=C1C1=CN=C(N)C=N1)S(C)(=O)=O |
| 181 | CC1=CC(Cl)=C(C=C1C1=CN=C(N)C=N1)S(=O)(=O)NCCCO |
| 182 | CC1=CC(Cl)=C(C=C1C1=CN=C2N1C=CN=C2N)S(C)(=O)=O |
| 183 | COC(=O)C1=C(NC(=O)C2=CC=C(F)C=C2)C2=C(S1)C1=C(C=C(OC)C(OC)=C1)N=C2 |
| 184 | CCN1N=NC(CCNC(=O)NC2=NC(=C(C)S2)C2=CC(F)=C(C=C2)S(C)(=O)=O)=N1 |
| 185 | CC1=NN=C(O1)C1=C(N)N=CC(=N1)C1=CC(=CC=C1C)S(=O)(=O)N[C@H]1CCC(O)CC1 |
| 186 | CCCN1C=NC(CCNC(=O)NC2=NC3=C(S2)C=C(C=C3)C2=CC(OC)=CN=C2)=C1 |
| 187 | COC1=C(Cl)N=CC(=C1)N1CCC2=C(C1)SC(NC(=O)NCCC1=CN(CC(F)F)C=N1)=N2 |
| 188 | CC1=C2C(=O)N(C3=CN(CC(F)F)N=C3)C(C)(C)C2=NC(=C1)C1=CC(=CN=C1)[C@@H](O)C(F)(F)F |
| 189 | COC1=C(OC)N=CC(=C1)C1=CC(C)=C2C(=O)N([C@H](C)C2=N1)C1=CN(CC(F)(F)F)N=C1 |
| 190 | CC1=CC=CC=C1N1C(CN2N=C(C3=C2C=CC=C3N)C2=CC=C(O)C(F)=C2)=NC2=C(C(C)=CC=C2)C1=O |
| 191 | CN1N=C(N=C1C1(CC1)C1=CC=CC=C1)C1=NC(=C(N)N=C1)C1=CC=C(C=C1)C(N)=O |
| 192 | C[C@@H](C1CC1)N1CC2=C(C1=O)C(C)=CC(=C2)C1=C(C)N=C(NC(C)=O)S1 |
| 193 | CC(=O)NC1=NC2=C(S1)C=CC(=C2)C1=NN(CC2=CC3=C(C(C)=CC=C3)C(=O)N2C2=CC=CC=C2C)C2=C1C(N)=NC=N2 |
| 194 | FC1(F)OC2=C(O1)C=C(\C=C1/SC(=O)NC1=O)C=C2 |
| 195 | ClC=1C=CC=C2C=C(N(C(C12)=O)C1=CC=CC=C1)[C@H](C)NC(=O)C=1C(=NN2C1N=CC=C2)N |
| 196 | ClC=1C=CC=C2C=C(N(C(C12)=O)C1=CC=CC=C1)[C@H](C)NC(=O)C=1C(=NC=C2C=CC=NC12)N |
| 197 | ClC=1C=CC=C2C=C(N(C(C12)=O)C1=CC=CC=C1)[C@H](C)NC(=O)C=1C=NN2C1N=CC=C2 |
| 198 | ClC=1C=CC=C2C=C(N(C(C12)=O)C1=CC=CC=C1)[C@H](C)NC(=O)C=1C=NC=C2C=CC=NC12 |
| 199 | ClC=1C=CC=C2C=C(N(C(C12)=O)C1=CC=CC=C1)[C@H](C)NC(=O)C=1C(=NN2C1C=CC=C2)N |
| 200 | ClC=1C=CC=C2C=C(N(C(C12)=O)C1=CC=CC=C1)[C@H](CC)NC(=O)C=1C(=NN2C1N=CC=C2)N |
| 201 | CC=1C=CC=C2C=C(N(C(C12)=O)C1=CC=CC=C1)[C@H](C)NC(=O)C=1C(=NN2C1N=CC=C2)N |
| 202 | FC=1C=CC=C2C=C(N(C(C12)=O)C1=CC=CC=C1)[C@H](C)NC(=O)C=1C(=NN2C1N=CC=C2)N |
| 203 | COC=1C=CC=C2C=C(N(C(C12)=O)C1=CC=CC=C1)[C@H](C)NC(=O)C=1C(=NN2C1N=CC=C2)N |
| 204 | CNC=1C=CC=C2C=C(N(C(C12)=O)C1=CC=CC=C1)[C@H](C)NC(=O)C=1C(=NN2C1N=CC=C2)N |
| 205 | CN(C=1C=CC=C2C=C(N(C(C12)=O)C1=CC=CC=C1)[C@H](C)NC(=O)C=1C(=NN2C1N=CC=C2)N)C |
| 206 | O=C1N(C(=CC2=CC=CC(=C12)C=CC)[C@H](C)NC(=O)C=1C(=NN2C1N=CC=C2)N)C2=CC=CC=C2 |
| 207 | C(#C)C=1C=CC=C2C=C(N(C(C12)=O)C1=CC=CC=C1)[C@H](C)NC(=O)C=1C(=NN2C1N=CC=C2)N |
| 208 | O=C1N(C(=CC2=CC=CC(=C12)C#CC)[C@H](C)NC(=O)C=1C(=NN2C1N=CC=C2)N)C2=CC=CC=C2 |
| 209 | O=C1N(C(=CC2=CC=CC(=C12)C#CC1=CC=CC=C1)[C@H](C)NC(=O)C=1C(=NN2C1N=CC=C2)N)C2=CC=CC=C2 |
| 210 | O=C1N(C(=CC2=CC=CC(=C12)C#CC=1C=NC=CC1)[C@H](C)NC(=O)C=1C(=NN2C1N=CC=C2)N)C2=CC=CC=C2 |
| 211 | O=C1N(C(=CC2=CC=CC(=C12)C#CC1=CC=NC=C1)[C@H](C)NC(=O)C=1C(=NN2C1N=CC=C2)N)C2=CC=CC=C2 |
| 212 | O=C1N(C(=CC2=CC=CC(=C12)C#CC1=NC=CC=C1)[C@H](C)NC(=O)C=1C(=NN2C1N=CC=C2)N)C2=CC=CC=C2 |
| 213 | O=C1N(C(=CC2=CC=CC(=C12)C#CC1=CN=CS1)[C@H](C)NC(=O)C=1C(=NN2C1N=CC=C2)N)C2=CC=CC=C2 |
| 214 | O=C1N(C(=CC2=CC=CC(=C12)C#CC=1N=CSC1)[C@H](C)NC(=O)C=1C(=NN2C1N=CC=C2)N)C2=CC=CC=C2 |
| 215 | O=C1N(C(=CC2=CC=CC(=C12)C#CC=1SC=CN1)[C@H](C)NC(=O)C=1C(=NN2C1N=CC=C2)N)C2=CC=CC=C2 |
| 216 | CN1N=C(C=C1)C#CC=1C=CC=C2C=C(N(C(C12)=O)C1=CC=CC=C1)[C@H](C)NC(=O)C=1C(=NN2C1N=CC=C2)N |
| 217 | C1=CC(=CC(=C1)O)C2=NC3=C(N=C(N=C3N=C2C4=CC(=CC=C4)O)N)N |
| 218 | CNC(=O)NC1=NN2C=C(C=CC2=N1)C1=CN=CC(=C1)S(=O)(=O)NC(C)(C)C |
| 219 | CC(C)(C)NS(=O)(=O)C1=CC(=CN=C1)C1=CN2N=C(NC(=O)N3CCOCC3)N=C2C=C1 |
| 220 | CN(C)CCNC(=O)NC1=NN2C=C(C=CC2=N1)C1=CN=CC(=C1)S(=O)(=O)NC(C)(C)C |
| 221 | CC(C)(C)NS(=O)(=O)C1=CC(=CN=C1)C1=CN2N=C(NC(=O)NCCN3CCOCC3)N=C2C=C1 |
| 222 | CC(C)(C)NS(=O)(=O)C1=CC(=CN=C1)C1=CN2N=C(NC(=O)NC3=CC=CC=C3)N=C2C=C1 |
| 223 | CC(C)(C)NS(=O)(=O)C1=CC(=CN=C1)C1=CN2N=C(NC(=O)NCC3=CC=CC=C3)N=C2C=C1 |
| 224 | CC(C)(C)NS(=O)(=O)C1=CC(=CN=C1)C1=CN2N=C(NC(=O)NCCC3=CC=CC=C3)N=C2C=C1 |
| 225 | CC1=C(CCNC(=O)NC2=NN3C=C(C=CC3=N2)C2=CN=CC(=C2)S(=O)(=O)NC(C)(C)C)C(C)=NO1 |
| 226 | CC(C)(C)NS(=O)(=O)C1=CC(=CN=C1)C1=CN2N=C(NC(=O)NCCN3C=CC=N3)N=C2C=C1 |
| 227 | CC1=NC(CCNC(=O)NC2=NN3C=C(C=CC3=N2)C2=CN=CC(=C2)S(=O)(=O)NC(C)(C)C)=CS1 |
| 228 | CC(C)(C)NS(=O)(=O)C1=CC(=CN=C1)C1=CN2N=C(NC(=O)NCCC3=CN=CC=N3)N=C2C=C1 |
| 229 | CC(C)(C)NS(=O)(=O)C1=CC(=CN=C1)C1=CN2N=C(NC(=O)NCCC3=NN=C(O3)C3CC3)N=C2C=C1 |
| 230 | CC(C)(C)NS(=O)(=O)C1=CN=CC(=C1)C1=CN2N=C(NC(=O)NCCN3N=NC(=N3)C3CC3)N=C2C=C1 |
| 231 | CC(C)(C)NS(=O)(=O)C1=CC(=CN=C1)C1=CN2N=C(NC(=O)NCCCN3N=NC(=N3)C3CC3)N=C2C=C1 |
| 232 | CN(C)C(=O)CNC(=O)NC1=NN2C=C(C=CC2=N1)C1=CN=CC(=C1)S(=O)(=O)NC(C)(C)C |
| 233 | CNC(=O)CNC(=O)NC1=NN2C=C(C=CC2=N1)C1=CN=CC(=C1)S(=O)(=O)NC(C)(C)C |
| 234 | CC(C)(C)NS(=O)(=O)C1=CC(=CN=C1)C1=CN2N=C(NC(=O)NCC(=O)N3CCCC3)N=C2C=C1 |
| 235 | CC(C)(C)NS(=O)(=O)C1=CC(=CN=C1)C1=CN2N=C(NC(=O)NCC(=O)N3CCOCC3)N=C2C=C1 |
| 236 | CN1CCN(CC1)C(=O)CNC(=O)NC1=NN2C=C(C=CC2=N1)C1=CN=CC(=C1)S(=O)(=O)NC(C)(C)C |
| 237 | NC1=NN2C=CC(=CC2=N1)C1=CN=CC(=C1)S(N)(=O)=O |
| 238 | CNS(=O)(=O)C1=CC(=CN=C1)C1=CC2=NC(N)=NN2C=C1 |
| 239 | CCCNS(=O)(=O)C1=CC(=CN=C1)C1=CC2=NC(N)=NN2C=C1 |
| 240 | NC1=NN2C=CC(=CC2=N1)C1=CN=CC(=C1)S(=O)(=O)NCC(F)(F)F |
| 241 | CC(C)(NS(=O)(=O)C1=CC(=CN=C1)C1=CC2=NC(N)=NN2C=C1)C(F)(F)F |
| 242 | NC1=NN2C=CC(=CC2=N1)C1=CN=CC(=C1)S(=O)(=O)NC1=CC=CC=C1 |
| 243 | CCN(CC)S(=O)(=O)C1=CC(=CN=C1)C1=CC2=NC(N)=NN2C=C1 |
| 244 | CC1(C)CCN1S(=O)(=O)C1=CC(=CN=C1)C1=CC2=NC(N)=NN2C=C1 |
| 245 | CC(C)(C)NS(=O)(=O)C1=CC(=CN=C1)C1=CC2=NC(N)=NN2C=C1 |

**Table S3.** Calculated parameters for checking drug-likeness of 245 compounds involved in the modeling process based on Lipinski’s rule of five

| No. | MW | nHDon | nHAcc | ClogP | TPSA(NO) | RBN | nAT |
| --- | --- | --- | --- | --- | --- | --- | --- |
| 1 | 433.60 | 1 | 7 | 2.5341 | 96.44 | 5 | 52 |
| 2 | 388.51 | 2 | 5 | 3.5828 | 76.44 | 4 | 53 |
| 3 | 347.46 | 2 | 5 | 1.9435 | 76.21 | 3 | 47 |
| 4 | 389.50 | 1 | 6 | 2.3245 | 79.29 | 4 | 52 |
| 5 | 347.46 | 2 | 5 | 1.9435 | 76.21 | 3 | 47 |
| 6 | 389.50 | 1 | 6 | 2.3245 | 79.29 | 4 | 52 |
| 7 | 347.46 | 2 | 5 | 1.5778 | 76.21 | 3 | 47 |
| 8 | 389.50 | 1 | 6 | 1.9588 | 79.29 | 4 | 52 |
| 9 | 427.56 | 2 | 6 | 1.3903 | 92.98 | 4 | 57 |
| 10 | 412.54 | 0 | 5 | 1.7162 | 66.96 | 4 | 55 |
| 11 | 347.46 | 2 | 5 | 1.5778 | 76.21 | 3 | 47 |
| 12 | 389.50 | 1 | 6 | 1.9588 | 79.29 | 4 | 52 |
| 13 | 427.56 | 2 | 6 | 1.2993 | 92.98 | 4 | 57 |
| 14 | 469.60 | 1 | 7 | 1.6803 | 96.06 | 5 | 62 |
| 15 | 412.54 | 0 | 5 | 1.6252 | 66.96 | 4 | 55 |
| 16 | 403.53 | 1 | 6 | 2.4132 | 79.29 | 5 | 55 |
| 17 | 419.53 | 1 | 7 | 1.4600 | 88.52 | 6 | 56 |
| 18 | 417.56 | 1 | 6 | 2.6315 | 79.29 | 5 | 58 |
| 19 | 451.57 | 1 | 6 | 3.4026 | 79.29 | 5 | 59 |
| 20 | 487.62 | 3 | 9 | 1.3959 | 125.62 | 5 | 65 |
| 21 | 457.59 | 3 | 7 | 0.6286 | 113.21 | 6 | 61 |
| 22 | 428.54 | 2 | 7 | 2.1338 | 102.24 | 4 | 56 |
| 23 | 413.53 | 3 | 6 | 1.2216 | 103.24 | 4 | 54 |
| 24 | 414.52 | 3 | 7 | 0.9907 | 116.13 | 4 | 53 |
| 25 | 424.55 | 2 | 6 | 2.3271 | 89.10 | 4 | 56 |
| 26 | 425.54 | 2 | 7 | 1.7648 | 101.99 | 4 | 55 |
| 27 | 481.60 | 3 | 7 | 2.9921 | 113.51 | 5 | 63 |
| 28 | 481.60 | 3 | 7 | 2.6463 | 113.51 | 6 | 63 |
| 29 | 443.47 | 1 | 9 | 2.4632 | 79.29 | 4 | 52 |
| 30 | 468.59 | 2 | 9 | 0.6636 | 125.46 | 6 | 57 |
| 31 | 468.59 | 2 | 9 | 0.7403 | 125.46 | 6 | 57 |
| 32 | 495.56 | 2 | 9 | 2.2016 | 92.98 | 4 | 60 |
| 33 | 495.56 | 2 | 9 | 2.2016 | 92.98 | 4 | 60 |
| 34 | 479.51 | 2 | 10 | 2.1620 | 101.99 | 4 | 55 |
| 35 | 563.63 | 3 | 10 | 4.3396 | 113.51 | 6 | 69 |
| 36 | 548.61 | 1 | 9 | 4.5690 | 87.49 | 6 | 67 |
| 37 | 408.54 | 1 | 4 | 3.8897 | 60.23 | 4 | 55 |
| 38 | 408.54 | 1 | 4 | 3.9807 | 60.23 | 4 | 55 |
| 39 | 466.63 | 2 | 5 | 4.2196 | 80.46 | 5 | 65 |
| 40 | 432.56 | 1 | 4 | 4.7262 | 71.13 | 4 | 57 |
| 41 | 423.56 | 3 | 5 | 3.5638 | 86.25 | 4 | 57 |
| 42 | 476.54 | 1 | 7 | 4.7920 | 60.23 | 4 | 58 |
| 43 | 438.57 | 1 | 5 | 2.7153 | 69.34 | 4 | 59 |
| 44 | 411.55 | 1 | 4 | 2.9529 | 64.11 | 4 | 56 |
| 45 | 400.57 | 2 | 4 | 3.7187 | 59.37 | 4 | 58 |
| 46 | 375.46 | 2 | 5 | 2.7165 | 84.64 | 4 | 49 |
| 47 | 451.57 | 2 | 6 | 3.3780 | 89.33 | 5 | 59 |
| 48 | 458.61 | 2 | 6 | 3.0619 | 85.67 | 5 | 64 |
| 49 | 451.56 | 2 | 5 | 4.3757 | 84.64 | 5 | 59 |
| 50 | 465.59 | 2 | 5 | 4.7196 | 84.64 | 5 | 62 |
| 51 | 493.65 | 2 | 5 | 5.5627 | 84.64 | 6 | 68 |
| 52 | 491.63 | 2 | 5 | 5.4865 | 84.64 | 6 | 66 |
| 53 | 479.62 | 2 | 5 | 5.0635 | 84.64 | 5 | 65 |
| 54 | 505.66 | 2 | 5 | 5.8304 | 84.64 | 6 | 69 |
| 55 | 469.55 | 2 | 6 | 4.4765 | 84.64 | 5 | 59 |
| 56 | 519.56 | 2 | 8 | 5.2240 | 84.64 | 5 | 62 |
| 57 | 481.59 | 2 | 6 | 4.3057 | 93.87 | 6 | 63 |
| 58 | 520.68 | 2 | 6 | 4.8667 | 87.88 | 6 | 71 |
| 59 | 451.56 | 2 | 5 | 4.3757 | 84.64 | 5 | 59 |
| 60 | 465.59 | 2 | 5 | 4.7196 | 84.64 | 5 | 62 |
| 61 | 451.56 | 2 | 5 | 4.3757 | 84.64 | 5 | 59 |
| 62 | 450.58 | 3 | 5 | 3.9779 | 90.43 | 5 | 60 |
| 63 | 486.64 | 3 | 6 | 3.6546 | 107.50 | 5 | 61 |
| 64 | 422.57 | 1 | 4 | 4.3441 | 60.23 | 5 | 58 |
| 65 | 434.58 | 1 | 4 | 4.2078 | 60.23 | 5 | 59 |
| 66 | 412.53 | 1 | 5 | 3.0728 | 69.46 | 5 | 55 |
| 67 | 424.54 | 1 | 5 | 3.1977 | 69.46 | 3 | 56 |
| 68 | 396.53 | 1 | 4 | 3.9522 | 60.23 | 3 | 54 |
| 69 | 422.57 | 1 | 4 | 4.2703 | 60.23 | 4 | 58 |
| 70 | 462.56 | 1 | 5 | 4.6116 | 60.23 | 4 | 58 |
| 71 | 422.44 | 1 | 7 | 3.6966 | 60.23 | 3 | 48 |
| 72 | 439.61 | 1 | 4 | 3.7960 | 64.11 | 5 | 62 |
| 73 | 427.55 | 1 | 5 | 2.5390 | 73.34 | 5 | 57 |
| 74 | 431.96 | 1 | 4 | 3.2150 | 64.11 | 4 | 53 |
| 75 | 422.53 | 1 | 5 | 2.4446 | 87.90 | 4 | 54 |
| 76 | 465.52 | 1 | 7 | 3.4573 | 64.11 | 4 | 56 |
| 77 | 454.58 | 2 | 6 | 2.0478 | 93.21 | 5 | 60 |
| 78 | 454.58 | 2 | 6 | 2.3127 | 93.21 | 5 | 60 |
| 79 | 490.64 | 2 | 7 | 1.7344 | 110.28 | 6 | 61 |
| 80 | 504.67 | 1 | 7 | 1.8276 | 101.49 | 6 | 64 |
| 81 | 490.64 | 2 | 7 | 1.6577 | 110.28 | 6 | 61 |
| 82 | 475.62 | 1 | 6 | 1.6110 | 98.25 | 5 | 59 |
| 83 | 519.56 | 2 | 8 | 5.2240 | 84.64 | 5 | 62 |
| 84 | 533.59 | 2 | 8 | 5.5679 | 84.64 | 5 | 65 |
| 85 | 465.59 | 2 | 5 | 4.3738 | 84.64 | 6 | 62 |
| 86 | 479.62 | 2 | 5 | 4.7177 | 84.64 | 6 | 65 |
| 87 | 493.65 | 2 | 5 | 5.1962 | 84.64 | 6 | 68 |
| 88 | 491.63 | 2 | 5 | 4.4971 | 84.64 | 6 | 66 |
| 89 | 545.60 | 2 | 8 | 5.0015 | 84.64 | 6 | 66 |
| 90 | 385.44 | 2 | 7 | 2.9392 | 90.62 | 4 | 45 |
| 91 | 374.42 | 3 | 8 | 1.3607 | 117.28 | 4 | 43 |
| 92 | 392.41 | 3 | 9 | 1.4615 | 117.28 | 4 | 43 |
| 93 | 459.93 | 3 | 8 | 1.6067 | 118.84 | 4 | 51 |
| 94 | 407.51 | 0 | 4 | 3.6214 | 63.50 | 4 | 52 |
| 95 | 410.45 | 2 | 11 | 1.4270 | 89.63 | 3 | 50 |
| 96 | 411.44 | 2 | 12 | 1.2142 | 102.52 | 3 | 49 |
| 97 | 606.70 | 3 | 15 | 2.6907 | 146.88 | 6 | 71 |
| 98 | 485.65 | 1 | 8 | 2.8000 | 91.38 | 6 | 67 |
| 99 | 488.61 | 3 | 10 | 2.4775 | 129.27 | 6 | 64 |
| 100 | 615.82 | 2 | 13 | 3.0405 | 128.29 | 7 | 86 |
| 101 | 513.62 | 1 | 12 | 2.2523 | 103.49 | 4 | 66 |
| 102 | 473.63 | 2 | 11 | -1.1253 | 134.77 | 5 | 60 |
| 103 | 354.47 | 2 | 8 | 1.0062 | 106.82 | 3 | 48 |
| 104 | 354.47 | 2 | 8 | 3.1230 | 106.82 | 3 | 48 |
| 105 | 354.47 | 2 | 8 | 1.4223 | 106.82 | 3 | 48 |
| 106 | 358.40 | 3 | 8 | 2.8946 | 123.52 | 5 | 41 |
| 107 | 485.64 | 3 | 12 | 1.0226 | 152.35 | 7 | 61 |
| 108 | 470.39 | 4 | 11 | 1.6371 | 151.91 | 6 | 47 |
| 109 | 289.36 | 1 | 3 | 2.0905 | 49.36 | 2 | 37 |
| 110 | 291.34 | 1 | 5 | 1.2208 | 71.12 | 2 | 35 |
| 111 | 351.40 | 1 | 7 | 1.0808 | 89.58 | 4 | 43 |
| 112 | 480.59 | 3 | 11 | 0.3339 | 140.96 | 8 | 63 |
| 113 | 525.05 | 3 | 8 | 2.9064 | 128.08 | 6 | 52 |
| 114 | 493.57 | 0 | 10 | 2.9918 | 125.20 | 4 | 60 |
| 115 | 378.47 | 0 | 7 | 2.7281 | 77.44 | 5 | 50 |
| 116 | 450.52 | 1 | 11 | 2.1617 | 83.48 | 5 | 57 |
| 117 | 458.57 | 1 | 9 | 2.5829 | 113.26 | 5 | 60 |
| 118 | 599.67 | 2 | 13 | 2.3122 | 131.98 | 9 | 69 |
| 119 | 406.45 | 0 | 7 | 2.8444 | 50.41 | 3 | 50 |
| 120 | 424.49 | 0 | 9 | 3.3700 | 60.25 | 3 | 48 |
| 121 | 457.53 | 1 | 10 | 0.8775 | 79.18 | 5 | 58 |
| 122 | 457.53 | 1 | 10 | 0.8775 | 79.18 | 5 | 58 |
| 123 | 457.52 | 1 | 9 | 3.0471 | 75.02 | 5 | 58 |
| 124 | 296.36 | 2 | 5 | 2.0754 | 79.21 | 2 | 38 |
| 125 | 364.45 | 3 | 8 | 0.7238 | 114.96 | 4 | 42 |
| 126 | 346.46 | 3 | 7 | 0.6230 | 114.96 | 4 | 42 |
| 127 | 473.62 | 2 | 10 | 0.2665 | 130.51 | 7 | 60 |
| 128 | 374.48 | 3 | 7 | 3.8462 | 104.21 | 5 | 39 |
| 129 | 305.31 | 1 | 8 | 3.0815 | 68.02 | 3 | 30 |
| 130 | 279.40 | 1 | 5 | 3.4196 | 68.02 | 3 | 36 |
| 131 | 323.42 | 2 | 8 | 1.9056 | 104.38 | 3 | 39 |
| 132 | 359.84 | 1 | 6 | 2.7285 | 75.44 | 4 | 43 |
| 133 | 375.91 | 1 | 5 | 3.7402 | 62.30 | 4 | 43 |
| 134 | 419.57 | 1 | 7 | 2.1362 | 96.44 | 5 | 49 |
| 135 | 282.33 | 1 | 5 | 1.6633 | 68.21 | 3 | 35 |
| 136 | 282.33 | 1 | 5 | 1.6633 | 68.21 | 3 | 35 |
| 137 | 282.33 | 1 | 5 | 1.6633 | 68.21 | 3 | 35 |
| 138 | 312.36 | 1 | 6 | 1.5933 | 77.44 | 4 | 39 |
| 139 | 330.40 | 1 | 6 | 0.7353 | 93.12 | 3 | 37 |
| 140 | 345.42 | 2 | 7 | 0.7820 | 105.15 | 4 | 39 |
| 141 | 345.42 | 2 | 7 | 0.8587 | 105.15 | 4 | 39 |
| 142 | 309.36 | 2 | 6 | 1.1721 | 88.08 | 3 | 38 |
| 143 | 387.51 | 2 | 7 | 2.1738 | 105.15 | 7 | 48 |
| 144 | 421.52 | 2 | 7 | 2.2768 | 105.15 | 6 | 49 |
| 145 | 253.29 | 1 | 5 | 0.7324 | 71.87 | 2 | 30 |
| 146 | 253.29 | 1 | 5 | 0.7324 | 71.87 | 2 | 30 |
| 147 | 303.35 | 1 | 5 | 1.9268 | 71.87 | 2 | 36 |
| 148 | 253.29 | 1 | 5 | 0.7324 | 71.87 | 2 | 30 |
| 149 | 256.30 | 1 | 5 | -0.2044 | 75.75 | 2 | 31 |
| 150 | 258.33 | 1 | 4 | 1.5177 | 58.98 | 2 | 28 |
| 151 | 283.32 | 1 | 6 | 0.6624 | 81.10 | 3 | 34 |
| 152 | 331.39 | 1 | 7 | -0.2656 | 106.01 | 3 | 36 |
| 153 | 346.41 | 2 | 8 | -0.2189 | 118.04 | 4 | 38 |
| 154 | 289.35 | 2 | 6 | -0.6466 | 102.93 | 2 | 31 |
| 155 | 413.55 | 2 | 7 | 2.1261 | 105.15 | 5 | 52 |
| 156 | 303.38 | 1 | 6 | -0.3223 | 88.94 | 3 | 34 |
| 157 | 317.41 | 0 | 6 | -0.0729 | 80.15 | 3 | 37 |
| 158 | 400.88 | 3 | 7 | 1.2565 | 114.96 | 4 | 40 |
| 159 | 400.88 | 3 | 7 | 1.2565 | 114.96 | 4 | 40 |
| 160 | 380.47 | 3 | 7 | 0.8949 | 114.96 | 5 | 43 |
| 161 | 346.46 | 3 | 7 | 0.6230 | 114.96 | 4 | 42 |
| 162 | 332.43 | 3 | 7 | 0.2424 | 114.96 | 4 | 39 |
| 163 | 344.44 | 3 | 7 | 0.2012 | 114.96 | 5 | 40 |
| 164 | 331.44 | 2 | 6 | 0.6425 | 102.93 | 3 | 40 |
| 164 | 345.42 | 1 | 7 | 0.0783 | 106.01 | 3 | 39 |
| 166 | 345.42 | 1 | 7 | 0.1323 | 106.01 | 3 | 39 |
| 167 | 323.79 | 2 | 6 | -0.0406 | 102.93 | 2 | 31 |
| 168 | 349.38 | 1 | 8 | -0.1648 | 106.01 | 3 | 36 |
| 169 | 415.47 | 2 | 7 | 2.8580 | 98.68 | 5 | 49 |
| 170 | 421.50 | 2 | 8 | 2.2507 | 99.21 | 4 | 46 |
| 171 | 477.93 | 5 | 11 | 1.6462 | 160.37 | 5 | 51 |
| 172 | 401.44 | 2 | 8 | 2.4036 | 98.68 | 4 | 46 |
| 173 | 416.90 | 2 | 6 | 3.9288 | 85.79 | 4 | 47 |
| 174 | 737.33 | 4 | 13 | 3.7714 | 159.91 | 14 | 94 |
| 175 | 719.76 | 4 | 16 | 3.3086 | 148.83 | 12 | 85 |
| 176 | 431.92 | 6 | 7 | 2.9538 | 144.85 | 4 | 49 |
| 177 | 422.49 | 1 | 6 | 2.1565 | 104.89 | 3 | 50 |
| 178 | 498.01 | 2 | 9 | 1.2918 | 127.12 | 3 | 54 |
| 179 | 414.89 | 2 | 6 | 2.3138 | 105.62 | 4 | 45 |
| 180 | 297.79 | 2 | 5 | 1.0340 | 85.94 | 2 | 31 |
| 181 | 356.87 | 4 | 7 | 1.0914 | 118.20 | 6 | 40 |
| 182 | 336.83 | 2 | 5 | 0.9774 | 90.04 | 2 | 35 |
| 183 | 440.48 | 1 | 8 | 3.9181 | 86.75 | 6 | 48 |
| 184 | 453.58 | 2 | 10 | 2.1641 | 130.71 | 7 | 50 |
| 185 | 444.57 | 4 | 10 | 1.4045 | 157.12 | 5 | 55 |
| 186 | 436.59 | 2 | 7 | 3.3886 | 92.91 | 8 | 55 |
| 187 | 498.01 | 2 | 10 | 1.9192 | 96.15 | 7 | 55 |
| 188 | 481.47 | 1 | 11 | 1.9514 | 83.09 | 4 | 54 |
| 189 | 447.46 | 0 | 10 | 2.3130 | 81.32 | 5 | 52 |
| 190 | 505.59 | 3 | 6 | 4.3705 | 96.86 | 4 | 62 |
| 191 | 411.51 | 4 | 7 | 1.8298 | 124.55 | 5 | 52 |
| 192 | 369.53 | 1 | 5 | 3.8760 | 62.30 | 4 | 49 |
| 193 | 586.73 | 3 | 9 | 5.2126 | 131.51 | 5 | 69 |
| 194 | 285.24 | 1 | 7 | 3.2632 | 68.39 | 1 | 24 |
| 195 | 458.94 | 3 | 7 | 2.6267 | 105.95 | 4 | 52 |
| 196 | 469.96 | 3 | 7 | 4.0391 | 101.85 | 4 | 54 |
| 197 | 443.92 | 1 | 6 | 2.9526 | 79.93 | 4 | 50 |
| 198 | 454.94 | 1 | 6 | 4.3650 | 75.83 | 4 | 52 |
| 199 | 457.95 | 3 | 6 | 3.3447 | 93.06 | 4 | 53 |
| 200 | 472.97 | 3 | 7 | 3.0811 | 105.95 | 5 | 55 |
| 201 | 438.53 | 3 | 7 | 2.3646 | 105.95 | 4 | 55 |
| 202 | 442.49 | 3 | 8 | 2.1215 | 105.95 | 4 | 52 |
| 203 | 454.53 | 3 | 8 | 1.9507 | 115.18 | 5 | 56 |
| 204 | 454.53 | 3 | 8 | 1.6677 | 115.18 | 5 | 56 |
| 205 | 467.58 | 3 | 8 | 1.9171 | 109.19 | 5 | 60 |
| 206 | 464.57 | 3 | 6 | 3.1113 | 105.95 | 5 | 59 |
| 207 | 448.52 | 3 | 7 | 2.1219 | 105.95 | 4 | 54 |
| 208 | 462.55 | 3 | 6 | 3.0420 | 105.95 | 4 | 57 |
| 209 | 524.62 | 3 | 6 | 4.2047 | 105.95 | 4 | 64 |
| 210 | 525.61 | 3 | 7 | 3.2038 | 118.84 | 4 | 63 |
| 211 | 525.61 | 3 | 7 | 3.2038 | 118.84 | 4 | 63 |
| 212 | 525.61 | 3 | 7 | 3.2578 | 118.84 | 4 | 63 |
| 213 | 531.64 | 3 | 7 | 3.0642 | 118.84 | 4 | 60 |
| 214 | 531.64 | 3 | 7 | 3.0360 | 118.84 | 4 | 60 |
| 215 | 531.64 | 3 | 7 | 3.0348 | 118.84 | 4 | 60 |
| 216 | 528.62 | 3 | 8 | 2.3210 | 122.72 | 4 | 64 |
| 217 | 346.38 | 6 | 8 | 1.9922 | 144.06 | 2 | 40 |
| 218 | 403.52 | 3 | 9 | 0.7330 | 130.07 | 5 | 49 |
| 219 | 459.59 | 2 | 10 | 0.9685 | 130.51 | 5 | 57 |
| 220 | 460.63 | 3 | 10 | 0.4328 | 133.31 | 8 | 60 |
| 221 | 502.67 | 3 | 11 | 0.4018 | 142.54 | 8 | 65 |
| 222 | 465.59 | 3 | 9 | 2.4417 | 130.07 | 6 | 56 |
| 223 | 479.62 | 3 | 9 | 2.1511 | 130.07 | 7 | 59 |
| 224 | 493.65 | 3 | 9 | 2.5812 | 130.07 | 8 | 62 |
| 225 | 512.66 | 3 | 11 | 1.7873 | 156.10 | 8 | 64 |
| 226 | 483.62 | 3 | 10 | 0.1400 | 146.84 | 8 | 59 |
| 227 | 514.70 | 3 | 10 | 1.8092 | 142.96 | 8 | 61 |
| 228 | 495.63 | 3 | 11 | 0.6334 | 155.85 | 8 | 60 |
| 229 | 525.66 | 3 | 12 | 1.6531 | 168.99 | 9 | 64 |
| 230 | 525.67 | 3 | 12 | 1.2517 | 172.62 | 9 | 64 |
| 231 | 539.70 | 3 | 12 | 1.7061 | 172.62 | 10 | 67 |
| 232 | 474.61 | 3 | 11 | 0.0832 | 150.38 | 7 | 59 |
| 233 | 460.58 | 4 | 11 | -0.1833 | 159.17 | 7 | 56 |
| 234 | 500.65 | 3 | 11 | 0.8742 | 150.38 | 7 | 63 |
| 235 | 516.65 | 3 | 12 | 0.0522 | 159.61 | 7 | 64 |
| 236 | 529.70 | 3 | 12 | 0.1616 | 153.62 | 7 | 68 |
| 237 | 290.34 | 4 | 7 | -0.8846 | 128.95 | 2 | 30 |
| 238 | 304.37 | 3 | 7 | -0.5232 | 114.96 | 3 | 33 |
| 239 | 332.43 | 3 | 7 | 0.3375 | 114.96 | 5 | 39 |
| 240 | 372.37 | 3 | 10 | 0.3674 | 114.96 | 4 | 36 |
| 241 | 400.43 | 3 | 10 | 1.1073 | 114.96 | 4 | 42 |
| 242 | 366.44 | 3 | 7 | 0.6505 | 114.96 | 4 | 40 |
| 243 | 346.46 | 2 | 7 | 0.3826 | 106.17 | 5 | 42 |
| 244 | 358.47 | 2 | 7 | 0.4969 | 106.17 | 3 | 43 |
| 245 | 346.46 | 3 | 7 | 0.6230 | 114.96 | 4 | 42 |

**Table S4.** The values of ten descriptors, extracted from the 245 PI3Kγ inhibitors (training and test sets) used to QSAR model development on pIC_50_ of these compounds

| No | Mor12p | RDF010e | Mor14u | Mor15m | GATS6p | Mor19m | Te | G2v | Mor02v | GATS4p |
| --- | --- | --- | --- | --- | --- | --- | --- | --- | --- | --- |
| 1 | -0.456 | 6.662 | 0.292 | 0.622 | 0.904 | 0.685 | 25.433 | 0.161 | 22.93 | 1.153 |
| 2 | -0.399 | 8.121 | 0.808 | 1.345 | 0.767 | 0.674 | 30.051 | 0.158 | 25.46 | 0.95 |
| 3 | -0.35 | 7.394 | 0.746 | 1.298 | 0.813 | 0.52 | 24.928 | 0.199 | 22.795 | 1.083 |
| 4 | -0.246 | 7.014 | 0.634 | 1.153 | 0.821 | 0.68 | 31.078 | 0.154 | 25.749 | 0.968 |
| 5 | -0.317 | 7.393 | 0.917 | 1.272 | 0.886 | 0.565 | 24.283 | 0.163 | 22.187 | 1.138 |
| 6 | -0.241 | 7.014 | 0.842 | 1.207 | 0.874 | 0.738 | 32.094 | 0.172 | 25.066 | 1.01 |
| 7 | -0.241 | 7.546 | 0.549 | 1.399 | 0.74 | 0.697 | 24.68 | 0.158 | 22.034 | 1.083 |
| 8 | -0.221 | 7.163 | 0.775 | 1.216 | 0.719 | 0.719 | 31.531 | 0.149 | 23.969 | 1.006 |
| 9 | -0.635 | 8.548 | 0.574 | 1.632 | 0.857 | 0.769 | 29.57 | 0.155 | 25.625 | 1.109 |
| 10 | -0.481 | 6.788 | 0.847 | 1.396 | 0.834 | 0.678 | 28.69 | 0.156 | 25.762 | 1.121 |
| 11 | -0.392 | 7.547 | 0.816 | 1.385 | 0.667 | 0.531 | 25.16 | 0.163 | 22.459 | 1.027 |
| 12 | -0.399 | 7.165 | 0.899 | 1.295 | 0.665 | 0.575 | 32.051 | 0.163 | 24.215 | 0.964 |
| 13 | -0.335 | 8.541 | 0.558 | 1.776 | 0.857 | 0.646 | 30.29 | 0.162 | 25.783 | 1.23 |
| 14 | -0.444 | 8.12 | 0.945 | 1.737 | 0.944 | 0.572 | 32.699 | 0.158 | 26.413 | 1.118 |
| 15 | -0.459 | 6.766 | 0.588 | 1.562 | 0.834 | 0.729 | 29.484 | 0.164 | 25.963 | 1.258 |
| 16 | -0.353 | 7.589 | 0.699 | 1.275 | 0.751 | 0.709 | 36.146 | 0.172 | 26.037 | 1.01 |
| 17 | -0.162 | 7.51 | 0.943 | 1.678 | 0.72 | 0.602 | 35.267 | 0.163 | 26.14 | 1.026 |
| 18 | -0.469 | 8.082 | 0.793 | 1.326 | 0.783 | 0.906 | 37.801 | 0.154 | 26.942 | 1.014 |
| 19 | -0.15 | 8.151 | 0.473 | 1.812 | 0.846 | 0.687 | 44.285 | 0.161 | 30.29 | 1.246 |
| 20 | -0.508 | 10.069 | 0.693 | 1.793 | 0.789 | 0.747 | 43.932 | 0.15 | 31.169 | 1.252 |
| 21 | -0.444 | 9.808 | 0.726 | 1.922 | 0.792 | 0.748 | 32.369 | 0.152 | 27.27 | 1.084 |
| 22 | -0.435 | 8.167 | 0.33 | 1.713 | 0.86 | 0.816 | 30.506 | 0.156 | 25.233 | 0.907 |
| 23 | -0.474 | 9.05 | 0.431 | 1.615 | 0.705 | 0.527 | 27.944 | 0.161 | 25.027 | 0.984 |
| 24 | -0.444 | 8.544 | 0.639 | 1.38 | 0.765 | 0.455 | 27.906 | 0.162 | 24.657 | 0.776 |
| 25 | -0.356 | 8.458 | 1.102 | 1.746 | 0.816 | 0.363 | 28.631 | 0.163 | 26.524 | 0.98 |
| 26 | -0.351 | 8.038 | 0.797 | 1.734 | 0.556 | 0.247 | 28.223 | 0.181 | 25.703 | 0.948 |
| 27 | -0.126 | 9.893 | 0.628 | 1.91 | 0.791 | 0.813 | 31.481 | 0.151 | 26.765 | 1.186 |
| 28 | -0.462 | 9.957 | 0.757 | 1.85 | 0.681 | 0.649 | 31.574 | 0.158 | 29.472 | 1.092 |
| 29 | -0.286 | 6.423 | 0.542 | 1.356 | 0.851 | 0.44 | 32.33 | 0.149 | 25.026 | 1.074 |
| 30 | -0.282 | 8.186 | 0.795 | 1.33 | 0.933 | 0.227 | 32.269 | 0.146 | 27.342 | 0.804 |
| 31 | -0.432 | 8.114 | 0.512 | 1.542 | 0.838 | 0.357 | 33.607 | 0.155 | 25.713 | 1.191 |
| 32 | -0.293 | 8.076 | -0.05 | 1.328 | 0.891 | 0.627 | 30.327 | 0.167 | 28.817 | 1.154 |
| 33 | -0.641 | 8.109 | 0.318 | 1.647 | 0.891 | 0.673 | 31.926 | 0.167 | 26.287 | 1.137 |
| 34 | -0.178 | 7.322 | 0.202 | 1.53 | 0.908 | 0.3 | 28.33 | 0.147 | 26.42 | 0.936 |
| 35 | -0.207 | 10.117 | 1.231 | 1.557 | 0.823 | 0.889 | 34.222 | 0.141 | 26.511 | 1.085 |
| 36 | -0.085 | 8.327 | 0.541 | 1.561 | 0.803 | 0.723 | 28.766 | 0.167 | 32.739 | 1.094 |
| 37 | -0.376 | 7.658 | 0.228 | 1.833 | 0.82 | 0.283 | 28.088 | 0.147 | 27.044 | 1.207 |
| 38 | -0.377 | 7.706 | 0.556 | 1.637 | 0.955 | 0.493 | 27.64 | 0.164 | 26.535 | 1.151 |
| 39 | -0.499 | 9.888 | 0.593 | 1.988 | 0.734 | 0.537 | 33.62 | 0.142 | 30.845 | 1.083 |
| 40 | -0.279 | 7.766 | 0.482 | 1.739 | 0.875 | 0.579 | 29.457 | 0.155 | 28.498 | 1.204 |
| 41 | -0.438 | 9.538 | 0.477 | 1.819 | 0.876 | 0.507 | 28.956 | 0.159 | 26.704 | 1.115 |
| 42 | -0.39 | 7.335 | 0.156 | 1.685 | 0.879 | 0.781 | 28.688 | 0.154 | 28.83 | 0.906 |
| 43 | -0.276 | 7.968 | 0.535 | 1.689 | 0.862 | 0.48 | 32.075 | 0.15 | 30.728 | 1.064 |
| 44 | -0.613 | 7.812 | 0.173 | 1.339 | 0.732 | 0.623 | 29.737 | 0.179 | 26.749 | 1.178 |
| 45 | -0.607 | 8.932 | 0.929 | 1.422 | 0.786 | 0.717 | 30.955 | 0.154 | 29.129 | 1.197 |
| 46 | -0.252 | 7.45 | 0.493 | 1.3 | 0.89 | 0.509 | 25.679 | 0.161 | 24.347 | 1.025 |
| 47 | -0.301 | 8.714 | 0.405 | 1.675 | 0.873 | 0.453 | 32.822 | 0.145 | 29.444 | 1.126 |
| 48 | -0.949 | 9.283 | 0.982 | 1.719 | 0.815 | 0.745 | 36.764 | 0.15 | 33.119 | 1.011 |
| 49 | -0.305 | 8.828 | 0.345 | 1.89 | 0.74 | 0.404 | 29.871 | 0.153 | 27.181 | 1.047 |
| 50 | -0.439 | 9.174 | 0.346 | 1.801 | 0.756 | 0.542 | 36.128 | 0.144 | 32.53 | 1.153 |
| 51 | -0.44 | 10.114 | 0.375 | 1.721 | 0.734 | 0.696 | 33.795 | 0.151 | 32.776 | 1.1 |
| 52 | -0.548 | 10.003 | 0.089 | 1.653 | 0.734 | 0.844 | 35.124 | 0.168 | 31.615 | 1.1 |
| 53 | -0.429 | 9.539 | 0.32 | 1.86 | 0.773 | 0.645 | 36.068 | 0.146 | 33.495 | 1.234 |
| 54 | -0.525 | 10.372 | 0.501 | 1.766 | 0.752 | 0.759 | 37.725 | 0.153 | 33.383 | 1.184 |
| 55 | -0.243 | 8.481 | -0.062 | 1.941 | 0.784 | 0.381 | 30.203 | 0.145 | 27.13 | 0.95 |
| 56 | -0.382 | 8.46 | -0.007 | 1.58 | 0.822 | 0.52 | 34.761 | 0.155 | 29.797 | 1.097 |
| 57 | -0.299 | 9.205 | -0.191 | 2.104 | 0.752 | 0.476 | 32.643 | 0.143 | 27.642 | 0.949 |
| 58 | -0.555 | 10.21 | 0.458 | 2.103 | 0.961 | 0.822 | 42.357 | 0.152 | 34.573 | 0.953 |
| 59 | -0.168 | 8.807 | 0.195 | 1.868 | 0.919 | 0.447 | 30.902 | 0.145 | 29.807 | 1.029 |
| 60 | -0.385 | 9.164 | 0.29 | 1.829 | 0.905 | 0.539 | 28.94 | 0.151 | 29.491 | 1.153 |
| 61 | 0.163 | 8.784 | 0.17 | 1.97 | 0.918 | 0.496 | 31.674 | 0.145 | 31.229 | 1.126 |
| 62 | -0.756 | 9.849 | 0.257 | 1.823 | 0.783 | 0.452 | 34.294 | 0.16 | 32.778 | 1.094 |
| 63 | -0.542 | 9.789 | 0.456 | 2.107 | 0.684 | 0.524 | 35.204 | 0.181 | 32.391 | 0.788 |
| 64 | -0.555 | 8.077 | 0.464 | 1.762 | 0.871 | 0.544 | 27.907 | 0.162 | 25.863 | 1.098 |
| 65 | -0.759 | 8.492 | -0.06 | 1.876 | 0.856 | 0.527 | 31.615 | 0.153 | 31.031 | 1.052 |
| 66 | -0.273 | 7.241 | 0.149 | 1.727 | 0.992 | 0.451 | 27.17 | 0.147 | 25.743 | 1.113 |
| 67 | -0.211 | 7.065 | 0.718 | 1.861 | 0.867 | 0.537 | 29.562 | 0.175 | 31.397 | 0.902 |
| 68 | -0.271 | 7.441 | -0.251 | 1.501 | 0.884 | 0.206 | 26.672 | 0.182 | 26.919 | 1.259 |
| 69 | -0.546 | 8.237 | -0.211 | 1.618 | 0.852 | 0.17 | 29.691 | 0.169 | 29.505 | 1.233 |
| 70 | 0.23 | 7.472 | 0.145 | 2.178 | 0.865 | 0.321 | 27.764 | 0.146 | 27.761 | 0.959 |
| 71 | -0.018 | 5.6 | -0.262 | 1.289 | 0.965 | 0.26 | 26.585 | 0.202 | 25.772 | 0.924 |
| 72 | -0.612 | 8.752 | 0.571 | 1.484 | 0.676 | 0.829 | 30.177 | 0.158 | 29.358 | 1.111 |
| 73 | -0.564 | 7.854 | -0.192 | 1.752 | 0.818 | 0.546 | 29.251 | 0.155 | 25.845 | 1.046 |
| 74 | -0.507 | 7.126 | 0.088 | 1.399 | 0.814 | 0.625 | 29.754 | 0.166 | 26.003 | 1.289 |
| 75 | -0.334 | 7.2 | 0.242 | 1.242 | 0.798 | 0.627 | 29.892 | 0.148 | 26.525 | 1.174 |
| 76 | -0.401 | 7.107 | 0.031 | 1.137 | 1.033 | 0.584 | 31.695 | 0.163 | 28.749 | 1.122 |
| 77 | -0.535 | 8.818 | 0.213 | 1.6 | 0.951 | 0.815 | 30.427 | 0.16 | 27.85 | 1.147 |
| 78 | -0.461 | 8.93 | 0.296 | 1.345 | 0.887 | 0.488 | 31.125 | 0.178 | 29.691 | 0.988 |
| 79 | -0.512 | 8.783 | -0.308 | 1.802 | 1.046 | 0.504 | 29.826 | 0.159 | 27.243 | 1.281 |
| 80 | -0.56 | 8.288 | 0.228 | 1.503 | 1.052 | 0.398 | 28.872 | 0.15 | 30.268 | 1.273 |
| 81 | -0.384 | 8.875 | 0.85 | 1.48 | 0.83 | 0.45 | 29.197 | 0.152 | 33.178 | 0.867 |
| 82 | -0.459 | 7.744 | 0 | 1.098 | 1.011 | 0.476 | 30.337 | 0.145 | 26.778 | 1.279 |
| 83 | -0.311 | 8.476 | 0.277 | 1.575 | 0.988 | 0.463 | 35.743 | 0.165 | 32.482 | 1.097 |
| 84 | -0.249 | 8.834 | -0.005 | 1.628 | 0.979 | 0.602 | 31.902 | 0.156 | 29.463 | 1.115 |
| 85 | -0.446 | 9.221 | 0.46 | 1.835 | 0.742 | 0.638 | 31.538 | 0.165 | 27.554 | 1.036 |
| 86 | -0.245 | 9.547 | 0.379 | 1.869 | 0.759 | 0.79 | 34.443 | 0.142 | 36.106 | 1.009 |
| 87 | -0.554 | 10.288 | 0.406 | 1.974 | 0.747 | 0.861 | 37.373 | 0.148 | 35.839 | 1.017 |
| 88 | -0.622 | 10.09 | 0.315 | 1.685 | 0.747 | 0.74 | 38.57 | 0.155 | 34.36 | 1.017 |
| 89 | -0.307 | 9.379 | 0.388 | 1.447 | 1.005 | 0.659 | 36.641 | 0.159 | 34.843 | 1.033 |
| 90 | 0.384 | 6.405 | -0.189 | 2.038 | 0.923 | 0.122 | 18.368 | 0.154 | 19.354 | 0.774 |
| 91 | 0.444 | 6.841 | 0.312 | 1.966 | 1.274 | 0.014 | 17.456 | 0.156 | 19.394 | 0.671 |
| 92 | 0.553 | 6.482 | 0.154 | 2.167 | 1.273 | -0.027 | 17.741 | 0.168 | 19.411 | 0.644 |
| 93 | 0.856 | 7.867 | 0.249 | 2.392 | 1.087 | -0.23 | 21.123 | 0.16 | 26.611 | 1.132 |
| 94 | 0.237 | 6.281 | 0.796 | 1.787 | 1.161 | 0.547 | 22.508 | 0.185 | 25.708 | 1.613 |
| 95 | -0.322 | 6.417 | 0.432 | 1.977 | 0.944 | 0.663 | 19.495 | 0.151 | 24.392 | 1.142 |
| 96 | -0.321 | 6.074 | 0.347 | 2.281 | 0.968 | 0.218 | 18.877 | 0.151 | 25.183 | 1.19 |
| 97 | 0.062 | 9.416 | 0.917 | 1.763 | 0.866 | 0.474 | 37.286 | 0.158 | 34.467 | 1.043 |
| 98 | -0.647 | 8.289 | 2.397 | 2.063 | 0.902 | 1.335 | 33.623 | 0.142 | 36.429 | 0.951 |
| 99 | -0.011 | 9.555 | 0.336 | 2.572 | 0.926 | 0.805 | 44.526 | 0.15 | 36.349 | 1.139 |
| 100 | -0.887 | 11.144 | 1.887 | 3.493 | 0.798 | 0.496 | 71.449 | 0.144 | 44.853 | 1.152 |
| 101 | -0.352 | 7.317 | 0.954 | 2.59 | 1.284 | 0.955 | 26.739 | 0.149 | 29.919 | 1.125 |
| 102 | -0.335 | 7.735 | 0.85 | 2.332 | 0.499 | 0.517 | 29.698 | 0.156 | 29.611 | 0.918 |
| 103 | -0.243 | 6.672 | 0.402 | 2.067 | 0.768 | 0.33 | 19.895 | 0.181 | 22.807 | 0.963 |
| 104 | -0.246 | 6.672 | 0.426 | 2.072 | 0.768 | 0.333 | 19.895 | 0.172 | 22.81 | 0.963 |
| 105 | -0.244 | 6.672 | 0.406 | 2.068 | 0.768 | 0.331 | 19.895 | 0.181 | 22.806 | 0.963 |
| 106 | 0.933 | 6.767 | 0.048 | 1.759 | 0.747 | 0.069 | 20.261 | 0.157 | 24.972 | 0.875 |
| 107 | -0.195 | 8.886 | 1.019 | 1.838 | 0.618 | 0.128 | 30.754 | 0.159 | 26.667 | 0.728 |
| 108 | 0.045 | 7.513 | 0.262 | 1.293 | 0.571 | -1.002 | 22.494 | 0.163 | 17.495 | 0.809 |
| 109 | 0.387 | 5.434 | 0.21 | 1.462 | 1.2 | -0.101 | 18.038 | 0.175 | 20.738 | 1.063 |
| 110 | 0.348 | 4.636 | 0.02 | 1.463 | 0.884 | -0.25 | 17.439 | 0.163 | 20.182 | 0.926 |
| 111 | 0.519 | 5.375 | -0.014 | 1.877 | 0.919 | -0.242 | 22.539 | 0.168 | 21.695 | 0.952 |
| 112 | 0.061 | 8.94 | 0.446 | 2.479 | 0.933 | -0.025 | 39.117 | 0.151 | 30.398 | 0.831 |
| 113 | -0.164 | 7.726 | 0.617 | 1.246 | 0.702 | 0.085 | 35.496 | 0.185 | 30.104 | 1.25 |
| 114 | 0.768 | 5.972 | 0.547 | 1.954 | 0.774 | 0.343 | 27.414 | 0.16 | 32.854 | 0.944 |
| 115 | 0.135 | 5.698 | 0.132 | 2.337 | 0.972 | 0.11 | 24.297 | 0.169 | 29.073 | 0.834 |
| 116 | -0.377 | 6.407 | 0.427 | 1.944 | 1.162 | 0.461 | 26.49 | 0.155 | 24.894 | 0.636 |
| 117 | -0.222 | 7.132 | 1.216 | 1.701 | 1.116 | 0.493 | 27.839 | 0.145 | 28.403 | 0.929 |
| 118 | 0.158 | 8.495 | 0.745 | 3.225 | 0.796 | -0.243 | 41.436 | 0.147 | 33.155 | 1.106 |
| 119 | -0.002 | 5.066 | 0.217 | 1.474 | 0.652 | 0.672 | 21.11 | 0.16 | 21.857 | 1.009 |
| 120 | -0.145 | 4.367 | 0.3 | 0.929 | 0.591 | 0.675 | 17.046 | 0.172 | 21.62 | 0.989 |
| 121 | -0.049 | 6.837 | 1.137 | 2.089 | 0.857 | 0.219 | 21.767 | 0.158 | 26.879 | 0.59 |
| 122 | 0.121 | 6.782 | 1.483 | 2.686 | 0.799 | 0.158 | 21.727 | 0.162 | 25.582 | 0.596 |
| 123 | 0.597 | 6.837 | 0.689 | 2.018 | 0.786 | 0.237 | 21.665 | 0.154 | 26.65 | 0.748 |
| 124 | 0.287 | 5.986 | 0.211 | 0.998 | 1.24 | 0.418 | 22.593 | 0.2 | 18.49 | 0.748 |
| 125 | -0.01 | 6.986 | 0.373 | 1.372 | 1.055 | -0.164 | 23.084 | 0.198 | 19.55 | 0.992 |
| 126 | -0.085 | 7.34 | 0.702 | 1.281 | 1.15 | -0.066 | 22.479 | 0.169 | 19.922 | 0.962 |
| 127 | -0.543 | 8.229 | 0.751 | 1.572 | 0.968 | 0.575 | 42.005 | 0.153 | 28.626 | 1.06 |
| 128 | 0.185 | 6.48 | 0.387 | -0.071 | 0.965 | -0.117 | 24.653 | 0.173 | 17.964 | 1.028 |
| 129 | -0.108 | 3.372 | 0.218 | 0.036 | 0.78 | 0.572 | 17.943 | 0.169 | 11.971 | 0.839 |
| 130 | -0.361 | 5.104 | 0.252 | 0.188 | 0.985 | 0.541 | 18.207 | 0.177 | 13.958 | 1.285 |
| 131 | -0.058 | 5.42 | 1.149 | 0.302 | 0.88 | 0.687 | 22.387 | 0.173 | 19.036 | 1.427 |
| 132 | -0.554 | 5.762 | 0.289 | 1.498 | 0.833 | 0.343 | 26.985 | 0.178 | 21.167 | 1.186 |
| 133 | -0.541 | 5.694 | 0.255 | 0.772 | 0.997 | 0.021 | 25.744 | 0.156 | 20.356 | 1.246 |
| 134 | -0.326 | 6.313 | 0.049 | 0.927 | 1.507 | -0.06 | 26.575 | 0.151 | 22.922 | 1.225 |
| 135 | 0.509 | 4.877 | 0.293 | 1.104 | 1.083 | 0.143 | 19.575 | 0.163 | 15.961 | 0.97 |
| 136 | 0.386 | 4.904 | 0.434 | 1.141 | 1.003 | 0.201 | 22.619 | 0.193 | 18.028 | 0.91 |
| 137 | 0.42 | 4.89 | 0.561 | 1.153 | 1.214 | 0.27 | 23.066 | 0.163 | 18.134 | 0.809 |
| 138 | 0.645 | 5.25 | 0.232 | 1.465 | 1.053 | 0.296 | 24.989 | 0.185 | 18.552 | 0.988 |
| 139 | 0.07 | 4.789 | 0.49 | 0.824 | 1.122 | 0.042 | 22.793 | 0.189 | 18.062 | 0.772 |
| 140 | 0.269 | 5.922 | 0.304 | 0.997 | 0.918 | 0.175 | 22.555 | 0.159 | 18.916 | 0.786 |
| 141 | 0.13 | 5.781 | 0.976 | 0.969 | 1.114 | 0.006 | 22.841 | 0.185 | 18.18 | 0.786 |
| 142 | 0.322 | 5.857 | 0.528 | 0.921 | 1.18 | 0.274 | 23.995 | 0.174 | 19.278 | 0.907 |
| 143 | -0.253 | 7.112 | 1.302 | 1.184 | 1.11 | 0.343 | 34.237 | 0.177 | 23.351 | 0.985 |
| 144 | 0.003 | 7.244 | 0.805 | 1.544 | 1.246 | 0.107 | 29.856 | 0.171 | 24.96 | 1.115 |
| 145 | 0.404 | 4.134 | 0.446 | 0.867 | 1.1 | 0.1 | 16.86 | 0.169 | 16.998 | 0.987 |
| 146 | 0.368 | 4.128 | 0.37 | 0.822 | 1.1 | 0.121 | 17.926 | 0.169 | 16.178 | 0.987 |
| 147 | 0.643 | 4.786 | 0.653 | 1.259 | 1.098 | 0.176 | 20.666 | 0.184 | 18.075 | 0.906 |
| 148 | 0.342 | 4.148 | 0.395 | 0.823 | 1.1 | 0.04 | 16.578 | 0.179 | 17.015 | 0.907 |
| 149 | 0.239 | 4.287 | 0.137 | 0.656 | 1.043 | 0.306 | 17.994 | 0.177 | 16.647 | 1.086 |
| 150 | 0.444 | 4.059 | 0.226 | 0.252 | 1.269 | 0.228 | 15.292 | 0.172 | 16.225 | 0.76 |
| 151 | 0.474 | 4.483 | 0.241 | 1.021 | 0.956 | 0.118 | 20.718 | 0.164 | 16.402 | 1.045 |
| 152 | 0.181 | 4.386 | 0.148 | 0.743 | 1.086 | -0.074 | 22.247 | 0.177 | 18.025 | 0.723 |
| 153 | 0.333 | 5.518 | 0.167 | 1.116 | 0.894 | 0.07 | 25.114 | 0.16 | 18.931 | 0.949 |
| 154 | 0.082 | 4.764 | 0.156 | 0.857 | 1.207 | -0.06 | 17.19 | 0.168 | 16.281 | 0.76 |
| 155 | -0.414 | 7.536 | 1.973 | 1.329 | 0.999 | 0.544 | 35.734 | 0.167 | 28.139 | 0.94 |
| 156 | 0.142 | 4.303 | 0.231 | 0.947 | 1.14 | -0.079 | 19.219 | 0.188 | 17.642 | 0.77 |
| 157 | 0.231 | 3.86 | 0.556 | 1.12 | 1.084 | 0.096 | 22.293 | 0.189 | 18.143 | 0.781 |
| 158 | -0.27 | 6.51 | 0.419 | 1.107 | 1.141 | -0.342 | 20.233 | 0.171 | 22.045 | 1.277 |
| 159 | -0.084 | 6.49 | 0.186 | 1.321 | 1.123 | -0.363 | 18.585 | 0.158 | 23.611 | 1.064 |
| 160 | 0.028 | 7.221 | 0.944 | 1.334 | 1.33 | -0.001 | 21.531 | 0.156 | 20.836 | 1.078 |
| 161 | -0.145 | 7.353 | 0.682 | 1.229 | 0.94 | -0.074 | 19.933 | 0.156 | 19.93 | 0.933 |
| 162 | -0.26 | 6.783 | 0.631 | 1.143 | 1.158 | -0.017 | 19.669 | 0.159 | 18.3 | 0.883 |
| 163 | -0.35 | 7.051 | 0.721 | 1.188 | 1.073 | 0.082 | 21.266 | 0.158 | 21.454 | 1.001 |
| 164 | -0.211 | 6.185 | 0.563 | 0.735 | 1.038 | -0.083 | 19.424 | 0.189 | 19.408 | 0.693 |
| 165 | 0.082 | 4.718 | 0.285 | 0.655 | 1.039 | -0.021 | 22.124 | 0.166 | 18.181 | 0.691 |
| 166 | 0.228 | 4.759 | 0.356 | 0.869 | 1.108 | 0.069 | 21.75 | 0.166 | 17.433 | 0.672 |
| 167 | 0.011 | 4.416 | 0.026 | 0.846 | 1.194 | -0.193 | 17.339 | 0.168 | 16.271 | 0.757 |
| 168 | 0.287 | 4.039 | -0.138 | 0.935 | 1.075 | -0.158 | 22.185 | 0.184 | 17.876 | 0.817 |
| 169 | 0.463 | 6.816 | 0.392 | 1.995 | 0.988 | 0.139 | 18.32 | 0.161 | 22.738 | 1.027 |
| 170 | 0.331 | 6.175 | 0.605 | 1.266 | 0.782 | 0.352 | 19.389 | 0.153 | 22.587 | 1.064 |
| 171 | 0.422 | 9.158 | 0.157 | 2.414 | 0.89 | -0.005 | 18.166 | 0.173 | 20.34 | 1.172 |
| 172 | 0.529 | 6.415 | -0.275 | 2.087 | 0.93 | 0.302 | 19.564 | 0.153 | 22.784 | 1.098 |
| 173 | 0.376 | 6.744 | 0.309 | 1.891 | 0.843 | 0.113 | 17.887 | 0.153 | 23.808 | 1.317 |
| 174 | -0.297 | 13.519 | 2.713 | 2.953 | 1.093 | 0.109 | 46.536 | 0.137 | 40.251 | 1.12 |
| 175 | 0.39 | 11.611 | 0.309 | 3.228 | 0.971 | 0.431 | 29.443 | 0.14 | 26.097 | 1.078 |
| 176 | 0.414 | 10.204 | 1.003 | 1.255 | 0.854 | -0.022 | 19.923 | 0.151 | 22.228 | 0.677 |
| 177 | 0.017 | 6.352 | -0.091 | 2.576 | 1.142 | 0.209 | 17.232 | 0.151 | 20.719 | 1.222 |
| 178 | -0.131 | 7.251 | 0.292 | 1.487 | 0.934 | 0.164 | 17.252 | 0.174 | 15.082 | 0.921 |
| 179 | -0.113 | 6.263 | 0.115 | 2.117 | 0.88 | 0.325 | 17.665 | 0.176 | 21.493 | 0.889 |
| 180 | -0.088 | 4.812 | 0.021 | 1.122 | 1.192 | 0.047 | 14.372 | 0.186 | 14.228 | 1.017 |
| 181 | -0.449 | 7.51 | 0.418 | 1.283 | 0.901 | 0.261 | 19.964 | 0.183 | 18.606 | 1.119 |
| 182 | 0.03 | 5.229 | -0.128 | 1.539 | 1.142 | -0.222 | 15.148 | 0.193 | 16.16 | 0.966 |
| 183 | 0.292 | 5.575 | -0.081 | 2.011 | 0.893 | 0.004 | 28.161 | 0.172 | 22.515 | 1.004 |
| 184 | -0.43 | 6.378 | 0.122 | 0.139 | 1.221 | 0.191 | 29.372 | 0.151 | 23.801 | 0.984 |
| 185 | -0.607 | 9.063 | 1.097 | 1.048 | 1.126 | 0.323 | 25.519 | 0.168 | 27.081 | 1.025 |
| 186 | -0.25 | 8.002 | 0.407 | 0.986 | 1.269 | 0.096 | 49.837 | 0.156 | 28.194 | 1.301 |
| 187 | -0.466 | 6.823 | 0.319 | 0.83 | 1.029 | 0.263 | 43.592 | 0.168 | 25.235 | 1.109 |
| 188 | -0.246 | 6.118 | 0.037 | 1.381 | 0.697 | 0.561 | 29.298 | 0.148 | 26.684 | 0.85 |
| 189 | 0.198 | 5.218 | -0.059 | 1.505 | 0.533 | 0.466 | 33.095 | 0.18 | 23.112 | 0.874 |
| 190 | 0.754 | 9.495 | -0.455 | 3.24 | 0.883 | 0.571 | 24.87 | 0.151 | 33.681 | 1.131 |
| 191 | 0.194 | 9.589 | 0.898 | 1.202 | 1.134 | 0.409 | 30.803 | 0.194 | 23.132 | 1.067 |
| 192 | -0.775 | 6.71 | 0.906 | 1.056 | 0.904 | 0.447 | 25.468 | 0.151 | 20.929 | 1.153 |
| 193 | 0.608 | 9.721 | -0.004 | 2.51 | 0.933 | 0.394 | 35.635 | 0.175 | 36.659 | 1.058 |
| 194 | 0.268 | 2.393 | -0.454 | 0.782 | 1.286 | -0.14 | 14.719 | 0.179 | 14.014 | 0.876 |
| 195 | 0.486 | 8.235 | 0.455 | 1.627 | 1.039 | -0.116 | 20.653 | 0.159 | 28.017 | 1.06 |
| 196 | 0.517 | 8.511 | 0.529 | 1.792 | 1.055 | 0.042 | 21.166 | 0.148 | 28.777 | 1.197 |
| 197 | 0.493 | 6.41 | 0.323 | 1.637 | 1.045 | 0.065 | 20.442 | 0.151 | 26.752 | 1.138 |
| 198 | 0.581 | 6.652 | 0.666 | 2.059 | 1.05 | -0.09 | 20.291 | 0.149 | 26.655 | 1.301 |
| 199 | 0.25 | 8.589 | 0.66 | 1.809 | 1.031 | 0.286 | 21.113 | 0.158 | 22.456 | 1.258 |
| 200 | 0.381 | 8.627 | 0.682 | 2.183 | 1.006 | -0.219 | 20.703 | 0.152 | 27.131 | 1.065 |
| 201 | 0.532 | 8.927 | 0.406 | 1.682 | 1.089 | 0.057 | 21.433 | 0.164 | 28.936 | 1.001 |
| 202 | 0.647 | 8.241 | 0.274 | 1.956 | 1.152 | 0.007 | 20.611 | 0.159 | 28.184 | 0.892 |
| 203 | 0.492 | 8.973 | 0.266 | 1.863 | 1.132 | -0.069 | 22.148 | 0.163 | 28.501 | 0.875 |
| 204 | 0.404 | 8.932 | 0.355 | 2.074 | 1.132 | -0.113 | 22.415 | 0.163 | 31.36 | 0.875 |
| 205 | 0.598 | 9.487 | 0.7 | 2.08 | 1.106 | -0.091 | 23.915 | 0.167 | 28.908 | 0.872 |
| 206 | 0.418 | 9.564 | 0.774 | 1.691 | 1.134 | 0.034 | 24.644 | 0.153 | 31.629 | 0.966 |
| 207 | 0.59 | 8.903 | 0.582 | 2.03 | 1.072 | -0.17 | 21.754 | 0.157 | 27.628 | 0.975 |
| 208 | 0.432 | 8.893 | 0.661 | 1.992 | 1.134 | -0.175 | 24.003 | 0.155 | 29.725 | 0.966 |
| 209 | 0.598 | 9.984 | 0.803 | 1.899 | 1.056 | -0.21 | 30.106 | 0.143 | 40.106 | 0.995 |
| 210 | 0.746 | 9.579 | 0.544 | 1.899 | 1.056 | -0.281 | 29.535 | 0.143 | 38.671 | 0.966 |
| 211 | 0.63 | 9.559 | 0.672 | 1.825 | 1.024 | -0.466 | 29.54 | 0.164 | 40.78 | 0.966 |
| 212 | 0.661 | 9.599 | 0.855 | 2.258 | 1.089 | -0.154 | 28.196 | 0.164 | 31 | 0.966 |
| 213 | 0.726 | 9.009 | 0.446 | 1.328 | 0.934 | -0.135 | 27.412 | 0.145 | 36.217 | 0.735 |
| 214 | 0.746 | 9.061 | 0.809 | 1.286 | 0.889 | 0.066 | 27.226 | 0.153 | 36.349 | 0.735 |
| 215 | 0.424 | 9.061 | 0.677 | 1.221 | 0.956 | -0.056 | 28.026 | 0.167 | 36.633 | 0.735 |
| 216 | 0.611 | 9.761 | 0.628 | 2.079 | 1.093 | 0.135 | 32.106 | 0.163 | 35.975 | 0.946 |
| 217 | 1.027 | 9.37 | -0.138 | 2.068 | 0.926 | -0.054 | 17.504 | 0.158 | 16.523 | 0.931 |
| 218 | -0.209 | 7.947 | 0.421 | 1.341 | 1.1 | 0.061 | 28.761 | 0.151 | 21.807 | 0.838 |
| 219 | -0.53 | 7.883 | 0.471 | 2.021 | 1.118 | 0.358 | 44.503 | 0.162 | 27.36 | 0.933 |
| 220 | -0.192 | 9.336 | 0.749 | 1.762 | 1.24 | 0.063 | 38.124 | 0.145 | 28.173 | 0.956 |
| 221 | -0.679 | 9.725 | 1.598 | 1.633 | 1.152 | 0.353 | 44.579 | 0.156 | 32.051 | 0.938 |
| 222 | 0.181 | 9.048 | 0.609 | 1.826 | 1.218 | -0.092 | 35.924 | 0.156 | 29.056 | 0.993 |
| 223 | -0.26 | 9.405 | 0.805 | 1.65 | 1.25 | 0.054 | 35.155 | 0.161 | 29.996 | 0.967 |
| 224 | -0.134 | 9.851 | 1.425 | 1.728 | 1.28 | 0.138 | 35.188 | 0.158 | 29.125 | 0.971 |
| 225 | -0.427 | 9.469 | 1.095 | 2.018 | 1.198 | 0.137 | 55.284 | 0.157 | 28.725 | 0.922 |
| 226 | 0.026 | 9.393 | 0.77 | 1.24 | 1.135 | 0.165 | 35.217 | 0.153 | 27.779 | 0.916 |
| 227 | -0.265 | 9.224 | 1.218 | 1.128 | 0.972 | 0.307 | 37.442 | 0.159 | 30.786 | 0.772 |
| 228 | -0.131 | 9.087 | 0.908 | 1.823 | 1.161 | -0.161 | 50.441 | 0.156 | 29.188 | 0.918 |
| 229 | -0.306 | 9.635 | 1.042 | 1.291 | 1.006 | 0.376 | 48.466 | 0.163 | 31.046 | 0.849 |
| 230 | -0.366 | 9.616 | 0.714 | 1.52 | 1.064 | 0.283 | 48.673 | 0.157 | 31.668 | 0.937 |
| 231 | -0.784 | 10.026 | 0.933 | 1.401 | 1.079 | 0.776 | 44.145 | 0.152 | 36.109 | 0.96 |
| 232 | -0.209 | 8.937 | 1.108 | 1.876 | 1.201 | 0.138 | 41.802 | 0.168 | 28.231 | 0.934 |
| 233 | -0.085 | 9.401 | 1.028 | 1.81 | 1.152 | 0.108 | 46.312 | 0.167 | 26.126 | 0.911 |
| 234 | -0.327 | 9.442 | 0.653 | 1.391 | 1.199 | 0.214 | 44.824 | 0.151 | 28.446 | 0.991 |
| 235 | -0.545 | 9.288 | 0.995 | 2.285 | 1.125 | 0.288 | 58.855 | 0.16 | 30.365 | 0.97 |
| 236 | -0.317 | 9.841 | 1.656 | 1.762 | 1.199 | 0.288 | 55.004 | 0.148 | 31.95 | 1.018 |
| 237 | 0.023 | 6.24 | 0.345 | 1.097 | 1.007 | -0.157 | 17.11 | 0.169 | 15.708 | 0.701 |
| 238 | -0.055 | 5.769 | 0.62 | 1.058 | 0.949 | -0.15 | 18.962 | 0.19 | 16.245 | 0.675 |
| 239 | -0.316 | 6.66 | 0.856 | 1.061 | 0.899 | -0.046 | 22.747 | 0.185 | 19.369 | 0.868 |
| 240 | -0.055 | 5.536 | 0.635 | 0.856 | 1.044 | 0.057 | 22.74 | 0.17 | 17.74 | 1.337 |
| 241 | -0.422 | 6.612 | 0.211 | 0.772 | 1.013 | 0.188 | 21.757 | 0.18 | 18.922 | 1.355 |
| 242 | -0.053 | 6.865 | 0.534 | 1.577 | 0.962 | -0.243 | 23.002 | 0.183 | 20.142 | 1.083 |
| 243 | -0.268 | 6.237 | 1.333 | 1.161 | 0.902 | 0.028 | 20.53 | 0.169 | 16.851 | 0.81 |
| 244 | -0.622 | 6.573 | 0.47 | 1.055 | 0.906 | 0.319 | 20.1 | 0.162 | 19.648 | 0.915 |
| 245 | -0.234 | 7.347 | 0.635 | 1.265 | 0.94 | -0.048 | 20.177 | 0.156 | 19.074 | 0.933 |

**Table S5.** Brief description of 2D-autocorrelation and 3D descriptors entered in QSAR models

| Molecular descriptors | Definition | Descriptor category |
| --- | --- | --- |
| GATS4p | Geary autocorrelation of lag 4 weighted by polarizability | 2D autocorrelations**^a^** |
| GATS6p | Geary autocorrelation of lag 6 weighted by polarizability |  |
| RDF010e | Radial Distribution Function - 010 / weighted by Sanderson electronegativity | RDF descriptors**^b^** |
| Mor14u | signal 14 / unweighted | 3D-MoRSE descriptors**^b^** |
| Mor15m | signal 15 / weighted by mass |  |
| Mor19m | signal 19 / weighted by mass |  |
| Mor02v | signal 02 / weighted by van der Waals volume |  |
| Mor12p | signal 12 / weighted by polarizability |  |
|  |  |  |
| G2v | 2nd component symmetry directional WHIM index / weighted by van der Waals volume | WHIM descriptors**^b^** |
| Te | T total size index / weighted by Sanderson electronegativity |  |

**^a^**Belong to 2D descriptors

**^b^**Belong to 3D descriptors

**Table S6.** The test compounds that were selected randomly at each time of out-of-sample testing validation

| Iteration | Molecules of the test set selected randomly by Minitab |
| --- | --- |
| 1 | 6 11 14 18 20 21 28 36 38 40 44 48 50 59 66 71 74 81 85 91 92 94 97 101 102 107 113 116 119 129 139 141 143 147 150 152 156 168 175 180 183 186 204 208 211 213 223 226 244 |
| 2 | 8 11 14 24 26 28 33 34 41 49 53 60 68 73 85 88 91 93 98 99 100 101 105 106 112 114 121 123 145 146 155 162 178 184 189 190 191 204 213 216 219 220 225 231 232 234 236 241 242 |
| 3 | 6 11 22 24 27 28 32 33 34 41 49 60 63 64 71 86 104 108 128 129 135 139 142 144 151 157 158 161 172 173 174 177 183 186 189 192 194 196 198 201 206 214 225 232 234 237 241 243 66 |
| 4 | 7 9 10 11 31 34 50 55 67 69 72 76 77 80 82 84 85 87 88 104 111 114 116 132 136 144 149 157 161 163 180 183 186 193 195 199 200 201 202 203 205 211 216 218 220 223 232 234 240 |
| 5 | 2 12 16 24 32 40 41 42 45 54 59 62 66 71 72 78 82 84 92 96 101 105 106 114 117 119 120 122 123 128 137 141 144 146 149 155 157 161 166 170 178 180 186 198 209 213 231 233 236 |
| 6 | 2 11 13 16 18 19 21 24 29 33 36 46 75 78 79 90 92 96 104 105 106 113 115 117 119 121 128 131 134 153 157 160 175 179 188 192 193 198 205 206 210 212 215 216 222 228 236 237 245 |
| 7 | 4 8 9 12 15 23 25 39 40 43 49 63 76 80 81 87 89 91 92 98 105 106 108 117 126 137 140 144 149 150 154 164 167 168 171 179 180 181 182 188 197 201 208 209 212 219 227 235 236 |
| 8 | 5 9 10 12 20 35 38 43 57 63 70 72 75 77 79 83 85 89 93 97 98 107 113 114 117 119 122 126 131 143 146 147 158 164 165 170 178 189 206 208 209 214 216 221 222 224 227 233 237 |
| 9 | 1 2 18 20 30 33 35 40 56 57 62 66 75 92 95 100 102 107 109 121 123 124 125 127 130 131 132 133 139 143 146 152 154 160 161 163 166 172 177 180 188 189 192 200 214 223 231 233 242 |
| 10 | 1 5 8 9 11 21 24 31 32 34 36 39 40 52 54 56 69 72 75 78 80 94 99 100 101 106 113 114 121 123 125 131 134 143 147 154 160 164 177 189 193 194 199 201 210 214 222 230 232 |

**Table S7**. Structures of 45 PI3Kγ inhibitors used as a validation set and corresponding experimental pIC_50_ values

| **Compd.** | **R** | | **Exp. (pIC_50_)** | **Ref.** |
| --- | --- | --- | --- | --- |
|  |  | |  |  |
| 246 |  | | 7.01 | 62 |
| 247 |  |  | 7.43 | 62 |
| 248 |  |  | 7.20 | 62 |
| 249 |  |  | 7.82 | 62 |
| 250 |  |  | 7.77 | 62 |
| 251 |  |  | 9.30 | 62 |
| 252 |  |  | 8.15 | 62 |
| 253 |  |  | 9.15 | 62 |
| 254 |  |  | 9.15 | 62 |
| **Compd.** | **Structures of PI3Kγ inhibitors** | | **Exp. (pIC_50_)** | **Ref.** |
| 255 |  | | 7.12 | 62 |
| 256 |  |  | 7.85 | 62 |
| 257 |  |  | 7.85 | 63 |
| 258 |  |  | 7.32 | 63 |
| 259 |  |  | 7.44 | 63 |
| 260 |  |  | 6.51 | 63 |
| 261 |  |  | 7.80 | 63 |
| 262 |  |  | 9.00 | 63 |
| 263 |  |  | 5.98 | 63 |
| 264 |  |  | 7.80 | 63 |
| 265 |  |  | 6.80 | 63 |
| 266 |  |  | 7.30 | 63 |
| 267 |  |  | 7.60 | 63 |
| 268 |  |  | 5.51 | 63 |
|  | | | | |
| **Compd.** | **R** | | **Exp. (pIC_50_)** | **Ref.** |
| 269 |  | | 6.80 | 64 |
| 270 |  |  | 7.60 | 64 |
| 271 |  |  | 8.10 | 64 |
| 272 |  |  | 7.20 | 64 |
| **Compd.** | **PI3Kγ inhibitor scaffold** | **R** | **Exp. (pIC_50_)** | **Ref.** |
| 273 |  | SO_2_NHMe | 9.10 | 64 |
| 274 |  | SO_2_NH(CH_2_)_2_OMe | 8.90 | 64 |
| 275 |  | SO_2_Me | 9.10 | 64 |
| 276 |  | SO_2_(CH_2_)_3_OH | 8.90 | 64 |
| 277 |  | NHSO_2_(CH_2_)_2_OMe | 9.00 | 64 |
| **Compd.** | **Structures of PI3Kγ inhibitors** | | **Exp. (pIC_50_)** | **Ref.** |
| 278 |  | | 6.30 | 65 |
| 279 |  |  | 5.20 | 65 |
| 280 |  |  | 7.60 | 65 |
| 281 |  |  | 7.55 | 65 |
| 282 |  |  | 5.00 | 65 |
| 283 |  |  | 5.72 | 65 |
| 284 |  |  | 6.79 | 17 |
| 285 |  |  | 6.42 | 17 |
| 286 |  |  | 5.89 | 17 |
| 287 |  |  | 7.68 | 33 |
| 288 |  |  | 8.70 | 33 |
| 289 |  |  | 7.20 | 25 |
| 290 |  |  | 7.30 | 25 |

**Table S8.** SMILES strings of validation set consisting of 45 PI3Kγ inhibitors

| **No.** | **SMILES strings** |
| --- | --- |
| 246 | ClC1=NC=C(C=C1NS(=O)(=O)C1=CC=CC=C1)C=1C=C2C(=NC1)NC=C2 |
| 247 | ClC1=NC=C(C=C1NS(=O)(=O)C1=CC=CC=C1)C=1C=C2C(=NC1)NN=C2 |
| 248 | ClC1=NC=C(C=C1NS(=O)(=O)C1=CC=CC=C1)C=1C=C2C(=NC1)NC(=C2)C |
| 249 | ClC1=NC=C(C=C1NS(=O)(=O)C1=CC=CC=C1)C=1C=C2C(=NC1)NC=C2C2=CC=CC=C2 |
| 250 | ClC1=NC=C(C=C1NS(=O)(=O)C1=CC=CC=C1)C=1C=C2C(=NC1)NN=C2C2=CC=CC=C2 |
| 251 | ClC1=NC=C(C=C1NS(=O)(=O)C1=CC=CC=C1)C=1C=C2C(=NC1)NC=C2C2=CC=NC=C2 |
| 252 | ClC1=NC=C(C=C1NS(=O)(=O)C1=CC=CC=C1)C=1C=C2C(=NC1)NN=C2C2=CC=NC=C2 |
| 253 | ClC1=NC=C(C=C1NS(=O)(=O)C1=C(C=CC=C1)F)C=1C=C2C(=NC1)NC=C2C2=CC=NC=C2 |
| 254 | ClC1=NC=C(C=C1NS(=O)(=O)C1=CC=C(C=C1)F)C=1C=C2C(=NC1)NC=C2C2=CC=NC=C2 |
| 255 | N1N=CC2=C(C=CC=C12)C=1N=C(C2=C(N1)C=C(S2)CN2CCN(CC2)S(=O)(=O)C)N2CCOCC2 |
| 256 | CC1=C(SC2=C1N=C(N=C2N3CCOCC3)C4=CN=C(N=C4)N)CN5CCN(CC5)C(=O)C |
| 257 | C[C@@H](C(N1CCN(CC1)CC2=C(C3=NC(C4=CN=C(N=C4)N)=NC(N5CCOCC5)=C3S2)C)=O)O |
| 258 | O=C1N2C(C([C@H](NC3=CC=CC=C3C(O)=O)C)=CC(C)=C2)=NC(N4CCOCC4)=C1 |
| 259 | NC1=NC=C(C2=C3C(N(S(=O)(C)=O)CC3)=NC(N4CCOCC4)=N2)C=N1 |
| 260 | O=C(C1=CN=C(N(CC2=CC3=NC(C4=CC=C(OC)N=C4)=NC(N5CCOCC5)=C3S2)C)N=C1)NO |
| 261 | OC(C)(C)C1=CC2=C(S1)C(N3CCOCC3)=NC(C4=CN=C(N)N=C4)=N2 |
| 262 | FC1=CC(F)=C(S(=O)(NC2=CC(C(C=C3)=CN4C3=NC=C(Cl)C4=O)=CN=C2OC)=O)C=C1 |
| 263 | CC(N=C1NC(C2CCCC2)=O)=C(S1)C3=CC=C(C(S(=O)(NC4=CC=C(O)C=C4)=O)=C3)OC |
| 264 | CCN1N=NC2=C1N=C(N=C2N3CCOCC3)C4=CC=C(C=C4)NC(NC5=CC=C(C=C5)C(N6CCN(CC6)C)=O)=O |
| 265 | O=C1C=C(N2CCOCC2)OC3=C1SC=C3C4=CC=C(OCCO5)C5=C4 |
| 266 | OC1=CC=CC(C2=NC3=C(N)N=C(N)N=C3N=C2)=C1 |
| 267 | NC1=NC=C(C2=C3N=C(C)N(C(C)C)C3=NC(N4CCOCC4)=N2)C=N1 |
| 268 | O=C(OC)NC1=CC=C(C2=NC(N3CCOCC3)=C4C(N(C5CCN(CC6=CC=CN=C6)CC5)N=C4)=N2)C=C1.[H]Cl.[H]Cl |
| 269 | ClC=1C=C(C=C2CN(C(C12)=O)[C@@H](C)C1CC1)C1=CC(=NO1)NC(C)=O |
| 270 | NC=1C(=NC=CN1)C=1C=C2CN(C(C2=C(C1)C)=O)[C@@H](C)C1CC1 |
| 271 | NC=1C(=NC(=CN1)C=1C=C2CN(C(C2=C(C1)C)=O)[C@@H](C)C1CC1)C(=O)NC |
| 272 | CC=1N=C(SC1C=1C=C2CNC(C2=C(C1)C)=O)NC(C)=O |
| 273 | C1(CC1)[C@H](C)N1C(C2=C(C=C(C=C2C1)C1=C(N=C(S1)NC(C)=O)C)S(NC)(=O)=O)=O |
| 274 | C1(CC1)[C@H](C)N1C(C2=C(C=C(C=C2C1)C1=C(N=C(S1)NC(C)=O)C)S(NCCOC)(=O)=O)=O |
| 275 | C1(CC1)[C@H](C)N1C(C2=C(C=C(C=C2C1)C1=C(N=C(S1)NC(C)=O)C)S(=O)(=O)C)=O |
| 276 | C1(CC1)[C@H](C)N1C(C2=C(C=C(C=C2C1)C1=C(N=C(S1)NC(C)=O)C)S(=O)(=O)CCCO)=O |
| 277 | C1(CC1)[C@H](C)N1C(C2=C(C=C(C=C2C1)C1=C(N=C(S1)NC(C)=O)C)NS(=O)(=O)CCOC)=O |
| 278 | C[C@@H]1CN(C[C@@H](O1)C)CC1=CN=C(O1)C1=C2C=NNC2=CC(=C1)C=1C=C(C(=NC1)OC)NS(=O)(=O)C |
| 279 | N1C=CC2=C(C=CC=C12)C1=CC(=C2C=NNC2=C1)C=1OC(=CN1)CN1CCN(CC1)C(C)C |
| 280 | NC1=C2C(=NC=N1)N(N=C2C2=CC(=CC=C2)O)CC2=NC1=CC=CC(=C1C(N2CC2=C(C=CC=C2)Cl)=O)C#CCCCC(=O)N(CCOC)CCOC |
| 281 | NC1=C2C(=NC=N1)N(N=C2C2=CC(=CC(=C2)O)F)CC2=NC1=CC=CC(=C1C(N2CC2=C(C=CC=C2)C(F)(F)F)=O)C#CCOCCOCCOC |
| 282 | C[C@@H]1N(C2=CC=CC=C2C1)C(CC1=NC(=CC(N1)=O)N1CCOCC1)=O |
| 283 | NC=1OC2=C(N1)C=C(C=C2)C=2C=CC=1N(C2)C(=CN1)C(=O)N1CCOCC1 |
| 284 | O=C1N(C2=CC=CC=C2C)C(CN3N=C(C(C3=NC=N4)=C4N)C5=CC(O)=CC=C5)=NC6=CC=CC(C)=C16 |
| 285 | CC1=CC=CC2=C1C(=O)N(C(CN1N=C(C3=C1C=CC=C3N)C1=C3C=CNC3=CC=C1)=N2)C1=C(C)C=CC=C1 |
| 286 | CC1=CC=CC2=C1C(=O)N(C(CN1N=C(C#CCO)C3=C1C=CC=C3N)=N2)C1=C(C)C=CC=C1 |
| 287 | NC1=C2C(=NC=N1)N(N=C2C2=CC(=C(C=C2)O)F)CC2=NC1=CC=CC(=C1C(N2C2=C(C=CC=C2)C)=O)C |
| 288 | CCCN1C=NC(CNC(=O)NC2=NC3=C(S2)C=C(C=C3)C2=CC(OC)=CN=C2)=C1 |
| 289 | CC=1N=C(SC1C=1C=C2CNC(C2=C(C1)C)=O)NC(C)=O |
| 290 | CC=1N=C(SC1C=1C=C2CNC(C2=C(C1)S(N)(=O)=O)=O)NC(C)=O |

**Table S9.** The values of ten descriptors, extracted from the validation set consisting of 45 PI3Kγ inhibitors based on model 1 (Eq. **4**)

| No | Mor12p | RDF010e | Mor14u | Mor15m | GATS6p | Mor19m | Te | G2v | Mor02v | GATS4p |
| --- | --- | --- | --- | --- | --- | --- | --- | --- | --- | --- |
| 246 | -0.385 | 5.927 | 0.149 | 1.442 | 0.842 | -0.44 | 18.718 | 0.198 | 21.469 | 1.313 |
| 247 | -0.408 | 5.507 | 0.189 | 1.364 | 0.863 | -0.431 | 18.124 | 0.16 | 20.912 | 1.303 |
| 248 | -0.503 | 6.214 | 0.165 | 1.427 | 0.847 | -0.331 | 21.724 | 0.18 | 21.918 | 1.321 |
| 249 | -0.004 | 7.255 | 0.457 | 1.47 | 0.831 | -0.444 | 30.117 | 0.151 | 25.966 | 1.266 |
| 250 | -0.211 | 6.809 | 0.468 | 1.934 | 0.84 | -0.407 | 21.565 | 0.152 | 29.426 | 1.3 |
| 251 | 0.077 | 6.826 | -0.057 | 1.594 | 0.811 | -0.442 | 22.186 | 0.162 | 28 | 1.219 |
| 252 | -0.073 | 6.429 | 0.135 | 1.648 | 0.824 | -0.234 | 24.485 | 0.153 | 28.168 | 1.26 |
| 253 | 0.051 | 6.493 | -0.511 | 1.978 | 0.809 | -0.65 | 23.412 | 0.172 | 28.033 | 1.039 |
| 254 | 0.031 | 6.493 | -0.505 | 1.977 | 0.663 | -0.655 | 23.429 | 0.172 | 28.004 | 1.081 |
| 255 | -0.421 | 7.101 | 0.974 | 1.558 | 0.862 | 1.071 | 31.25 | 0.151 | 29.188 | 0.951 |
| 256 | -0.463 | 7.64 | 0.871 | 2.062 | 0.933 | 0.736 | 28.659 | 0.144 | 28.392 | 0.891 |
| 257 | -0.82 | 8.933 | 1.03 | 2.204 | 0.885 | 0.843 | 32.091 | 0.15 | 30.04 | 0.882 |
| 258 | -0.245 | 7.758 | 0.68 | 2.126 | 1.006 | 0.455 | 20.372 | 0.148 | 23.114 | 0.741 |
| 259 | -0.136 | 5.922 | -0.161 | 2.373 | 0.658 | -0.045 | 18.933 | 0.186 | 21.435 | 0.699 |
| 260 | 0.28 | 7.521 | 0.149 | 3.182 | 0.864 | 0.399 | 32.336 | 0.16 | 32.65 | 0.802 |
| 261 | -0.137 | 7.329 | -0.105 | 1.745 | 0.892 | 0.198 | 21.995 | 0.164 | 25.101 | 0.693 |
| 262 | 0.397 | 4.649 | -0.472 | 1.759 | 0.845 | -1.066 | 22.697 | 0.165 | 23.201 | 1.046 |
| 263 | -0.073 | 9.1 | 0.789 | 1.413 | 1.697 | 0.175 | 29.819 | 0.162 | 21.764 | 1.184 |
| 264 | -0.292 | 9.751 | 1.606 | 2.512 | 0.896 | 0.868 | 62.72 | 0.144 | 38.612 | 1.187 |
| 265 | 0.071 | 4.26 | 0.292 | 1.442 | 1.168 | 0.164 | 17.617 | 0.156 | 24.591 | 0.802 |
| 266 | 0.474 | 7.153 | -0.587 | 1.501 | 1.119 | -0.148 | 15.814 | 0.171 | 15.962 | 0.918 |
| 267 | -0.251 | 6.667 | 0.484 | 2.097 | 0.768 | 0.343 | 19.896 | 0.162 | 22.802 | 0.963 |
| 268 | -0.604 | 9.596 | 2.485 | 2.045 | 0.957 | 1.069 | 35.771 | 0.14 | 32.284 | 0.764 |
| 269 | -0.323 | 5.753 | 0.396 | 1.323 | 0.833 | 0.393 | 25.36 | 0.178 | 20.422 | 1.186 |
| 270 | -0.432 | 7.049 | 0.58 | 1.26 | 0.855 | 0.398 | 18.896 | 0.168 | 19.148 | 1.269 |
| 271 | -0.525 | 8.411 | 0.284 | 1.429 | 0.849 | 0.369 | 26.557 | 0.169 | 23.402 | 0.965 |
| 272 | -0.227 | 5.349 | -0.037 | 0.831 | 1.103 | 0.095 | 19.208 | 0.162 | 31.522 | 1.156 |
| 273 | -0.776 | 7.631 | 0.33 | 1.093 | 1.488 | -0.099 | 25.534 | 0.161 | 23.415 | 1.21 |
| 274 | -0.987 | 8.507 | 0.873 | 1.649 | 1.414 | -0.122 | 29.174 | 0.144 | 26.964 | 1.339 |
| 275 | -0.476 | 6.601 | 0.236 | 0.846 | 1.477 | 0.033 | 25.373 | 0.159 | 22.539 | 1.211 |
| 276 | -0.777 | 8.259 | 1.067 | 1.009 | 1.321 | 0.015 | 27.109 | 0.161 | 27.654 | 1.304 |
| 277 | -0.507 | 8.549 | 0.846 | 1.275 | 0.784 | 0.116 | 25.807 | 0.152 | 26.879 | 0.879 |
| 278 | -0.349 | 8.54 | -0.379 | 2.229 | 0.977 | 0.693 | 23.499 | 0.15 | 34.95 | 1.1 |
| 279 | -0.474 | 9.052 | 0.503 | 1.989 | 1.188 | 0.878 | 25.29 | 0.166 | 35.804 | 1.176 |
| 280 | -0.1 | 12.247 | 2.176 | 3.221 | 1.093 | 0.474 | 42.179 | 0.157 | 46.821 | 1.12 |
| 281 | 0.675 | 10.467 | 0.118 | 2.583 | 0.971 | 0.207 | 54.176 | 0.151 | 39.954 | 1.078 |
| 282 | -0.24 | 6.211 | 1.423 | 1.596 | 1.23 | 0.379 | 23.274 | 0.162 | 22.779 | 0.733 |
| 283 | 0.197 | 6.21 | 0.714 | 2.123 | 0.907 | 0.364 | 24.242 | 0.177 | 24.512 | 0.984 |
| 284 | 0.549 | 9.054 | -0.229 | 3.046 | 1.121 | 0.436 | 24.282 | 0.153 | 31.597 | 1.173 |
| 285 | 0.91 | 10.306 | -0.24 | 3.479 | 1.063 | 0.789 | 22.651 | 0.153 | 33.105 | 1.416 |
| 286 | 0.662 | 8.841 | 0.478 | 2.111 | 1 | 0.745 | 23.248 | 0.17 | 30.564 | 1.241 |
| 287 | 0.538 | 8.685 | -0.193 | 3.139 | 1.003 | 0.063 | 25.242 | 0.145 | 30.684 | 1.041 |
| 288 | -0.156 | 7.586 | 0.317 | 1.081 | 1.268 | 0.046 | 41.124 | 0.167 | 27.427 | 1.296 |
| 289 | -0.372 | 5.344 | 0.195 | 0.832 | 1.103 | 0.074 | 19.194 | 0.205 | 17.404 | 1.156 |
| 290 | -0.273 | 6.774 | -0.034 | 1.247 | 1.67 | -0.098 | 20.225 | 0.18 | 16.846 | 1.249 |
